# Evaluation of Waning of SARS-CoV-2 Vaccine–Induced Immunity: A Systematic Review and Meta-analysis

**DOI:** 10.1001/jamanetworkopen.2023.10650

**Published:** 2023-05-03

**Authors:** Francesco Menegale, Mattia Manica, Agnese Zardini, Giorgio Guzzetta, Valentina Marziano, Valeria d'Andrea, Filippo Trentini, Marco Ajelli, Piero Poletti, Stefano Merler

**Affiliations:** 1 Center for Health Emergencies, Bruno Kessler Foundation, Trento, Italy; 2 Department of Mathematics, University of Trento, Trento, Italy; 3 Epilab-JRU, FEM-FBK Joint Research Unit, Trento, Italy; 4 Dondena Centre for Research on Social Dynamics and Public Policy, Bocconi University, Milan, Italy; 5 Laboratory for Computational Epidemiology and Public Health, Department of Epidemiology and Biostatistics, Indiana University School of Public Health, Bloomington

## Abstract

**Importance:**

Estimates of the rate of waning of vaccine effectiveness (VE) against COVID-19 are key to assess population levels of protection and future needs for booster doses to face the resurgence of epidemic waves.

**Objective:**

To quantify the progressive waning of VE associated with the Delta and Omicron variants of SARS-CoV-2 by number of received doses.

**Data Sources:**

PubMed and Web of Science were searched from the databases’ inception to October 19, 2022, as well as reference lists of eligible articles. Preprints were included.

**Study Selection:**

Selected studies for this systematic review and meta-analysis were original articles reporting estimates of VE over time against laboratory-confirmed SARS-CoV-2 infection and symptomatic disease.

**Data Extraction and Synthesis:**

Estimates of VE at different time points from vaccination were retrieved from original studies. A secondary data analysis was performed to project VE at any time from last dose administration, improving the comparability across different studies and between the 2 considered variants. Pooled estimates were obtained from random-effects meta-analysis.

**Main Outcomes and Measures:**

Outcomes were VE against laboratory-confirmed Omicron or Delta infection and symptomatic disease and half-life and waning rate associated with vaccine-induced protection.

**Results:**

A total of 799 original articles and 149 reviews published in peer-reviewed journals and 35 preprints were identified. Of these, 40 studies were included in the analysis. Pooled estimates of VE of a primary vaccination cycle against laboratory-confirmed Omicron infection and symptomatic disease were both lower than 20% at 6 months from last dose administration. Booster doses restored VE to levels comparable to those acquired soon after the administration of the primary cycle. However, 9 months after booster administration, VE against Omicron was lower than 30% against laboratory-confirmed infection and symptomatic disease. The half-life of VE against symptomatic infection was estimated to be 87 days (95% CI, 67-129 days) for Omicron compared with 316 days (95% CI, 240-470 days) for Delta. Similar waning rates of VE were found for different age segments of the population.

**Conclusions and Relevance:**

These findings suggest that the effectiveness of COVID-19 vaccines against laboratory-confirmed Omicron or Delta infection and symptomatic disease rapidly wanes over time after the primary vaccination cycle and booster dose. These results can inform the design of appropriate targets and timing for future vaccination programs.

## Key Points

**Question:**

How does the effectiveness of COVID-19 vaccines against laboratory-confirmed Omicron infection and symptomatic disease change at different times from last dose administration and number of doses, and how does this compare with previously circulating SARS-CoV-2 variants and subvariants?

**Findings:**

This systematic review and meta-analysis of secondary data from 40 studies found that the estimated vaccine effectiveness against both laboratory-confirmed Omicron infection and symptomatic disease was lower than 20% at 6 months from the administration of the primary vaccination cycle and less than 30% at 9 months from the administration of a booster dose. Compared with the Delta variant, a more prominent and quicker waning of protection was found.

**Meaning:**

These findings suggest that the effectiveness of COVID-19 vaccines against Omicron rapidly wanes over time.

## Teaser

This systematic review and meta-analysis of studies reporting vaccine effectiveness at different time points since vaccine administration estimates the waning of protection provided by a variety of COVID-19 vaccine products.

## Introduction

Extensive vaccination programs have been performed around the globe to mitigate the effects of COVID-19.^1,2^ However, the progressive waning of vaccine-induced protection^3,4,5^ and the rapid replacement of the SARS-CoV-2 Delta variant by the Omicron variant in late 2021 to early 2022 have been associated with a marked increase of breakthrough infections among vaccinated individuals.^6,7,8^ In particular, Omicron seems to be characterized by both a lower initial vaccine effectiveness (VE) and a faster waning of protection against infection. Several studies^9,10,11,12,13,14,15,16,17,18,19,20,21,22,23,24,25,26,27,28,29,30,31,32,33,34,35,36,37,38,39,40,41,42,43,44,45,46,47,48^ have quantified the waning of VE against SARS-CoV-2 infection and symptomatic disease, but the obtained estimates are hard to reconcile because of differences in study design and temporal end points. Putting together the bulk of available evidence on the waning of VE over time against COVID-19 variants has crucial implications for future interventions and vaccination programs. A solid mathematical description of temporal changes in VE may have extensive applications for epidemic models. Various decay functions have been proposed for the COVID-19 VE waning rate in modeling studies^49,50,51,52,53^ but not within a comprehensive framework comparing the available published evidence.

In this study, we performed a systematic literature review of studies that reported VE at different time points since vaccine administration to estimate the waning of protection provided by a variety of COVID-19 vaccine products. We then performed a meta-analysis of the collected data to provide a cohesive picture of the waning rate of VE against Omicron and Delta infection and symptomatic disease at any time from last dose administration, for different vaccine products, and numbers of received doses.

## Methods

### Search Strategy

The study followed the Preferred Reporting Items for Systematic Reviews and Meta-analyses (PRISMA) reporting guideline.^54^ We searched PubMed, Web of Science, and reference lists of identified studies for peer-reviewed articles and preprints providing evidence of COVID-19 VE over time, without restrictions on study design, language, place, or time of publication. The outcome of interest was VE at different time points. We did not search clinical trial registers because we were interested in population-level effectiveness. The following search terms were used: (“Efficacy” OR “Effectiveness”) AND (“Vaccine” OR “Vaccination”) AND (“SARS-CoV-2” OR “COVID-19”) AND (“infection*” OR “disease”) AND (“waning” OR “decreas*”). Titles and abstracts of manuscripts were screened from the databases’ inception until October 19, 2022, to identify articles providing estimates of VE against SARS-CoV-2 infection or symptomatic disease.

### Eligibility Criteria

After removing duplicates, we excluded studies not related to VE or providing results on antibody titer levels only. We scrutinized the full texts of the remaining manuscripts to identify relevant sources among articles cited therein, and we selected manuscripts fulfilling all of the following criteria: (1) studies that included data and estimates of VE expressed as a percentage from studies comparing vaccinated and unvaccinated individuals, and studies that analyzed vaccinated individuals only but considered the first 2 weeks after the first dose administration as a proxy for unvaccinated individuals; (2) studies that included data against Delta and/or Omicron variants; (3) studies that considered as end points laboratory-confirmed infection and/or symptomatic disease; (4) studies that considered the primary vaccination cycle (consisting of 1 or 2 doses depending on the schedule associated with different vaccine products) and/or the administration of a booster dose; (5) studies that provided VE estimates for at least 2 well-defined intervals (eg, from 3 to 4 weeks from vaccine administration); an open interval (eg, >6 months) was not considered well defined; and (6) studies that provided information on which variants were circulating during the VE assessment (either stating that Delta or Omicron were the dominant circulating variant in the study period or assessing effectiveness specifically against Delta or Omicron based on genomic sequencing).

### Data Selection

Two authors (F.M. and M.M.) assessed the eligibility of articles and extracted data from each study (eTables 1-4 in Supplement 1). Details are provided in eAppendix 1 in Supplement 1.

### Evaluation of Study Quality and Risk of Bias

We used the Newcastle-Ottawa quality assessment scale for observational studies^55^ to assess the methodologic quality and risk of bias of included studies. This scale assigns a maximum of 9 points to studies according to participant selection (4 points), study comparability (2 points), and study outcome of interest (3 points). We classified studies as having high (≤3 points), moderate (4-6 points), and low (≥7 points) risk of bias. Two authors (F.M. and M.M.) independently evaluated the study quality and assigned the quality points (eTables 5 and 6 in Supplement 1). Publication bias was not assessed because of the different intervals, variants, vaccine products, and end points associated with VE estimates retrieved from the selected articles.

### Statistical Analysis

To estimate the vaccine-induced protection at any time from the last dose administration, we modeled VE as an exponential decay function of time:

VE(*t*) = *Ae^−w t^*

where *t* represents the number of days from maximum protection (which is assumed to occur 14 days after the administration of any dose), *A* represents VE 14 days after the administration of the last dose, and *w* represents the waning rate associated with the vaccine-induced protection against the considered end point. Free model parameters (*A* and *w*) were estimated for each study via a Markov chain Monte Carlo approach. Model details are described in eAppendix 2 in Supplement 1. Once calibrated, the model was used to compare the estimated protection against Delta and Omicron variants as provided by different vaccine products and number of administered doses at 1, 3, 6, and 9 months from last dose administration. We estimated the mean half-life of vaccine-induced protection as log(2)/*w* + 14 days, representing the time taken for VE to decrease to half of the estimated value of *A*. This approach allowed us to compare the mean VE obtained from different studies at any time from the administration of the last dose and to project VE beyond the final observation in the original studies.

Modeled VE estimates at different time points (1, 3, 6, and 9 months since administration of the last dose) were pooled using the inverse variance method implemented in the R package meta, software version 4.1.2 (R Foundation for Statistical Computing). The Cochran *Q* test and *I^2^* statistic were reported as measures of heterogeneity: we considered *I^2^* values of 25%, 50%, and 75% as indicators of low, moderate, and high heterogeneity, respectively.^56^

To explore potential biases resulting from the selection of the analyzed data points, we conducted 2 sensitivity analyses. In the first one (SA1), to evaluate the potential impact of the ramp-up of vaccine protection, we included only data points from the original studies in which VE was estimated at least 30 days after the administration of the last dose or data points that include observations in a period of at least 60 days after the administration of the last dose. In the second sensitivity analysis (SA2), we excluded studies in which VE was estimated by assuming that individuals who have received a single dose not earlier than 14 days represent a proxy for unvaccinated individuals. Finally, an additional analysis was conducted to investigate potential differences in effectiveness between children or younger adults and older adults. In this additional analysis, we included studies providing VE estimates against Delta or Omicron laboratory-confirmed infection or symptomatic disease of any vaccine product for age groups entirely contained in the range of 0 to 25 years or in the 60 years or older range.

## Results

**Figure 1.**
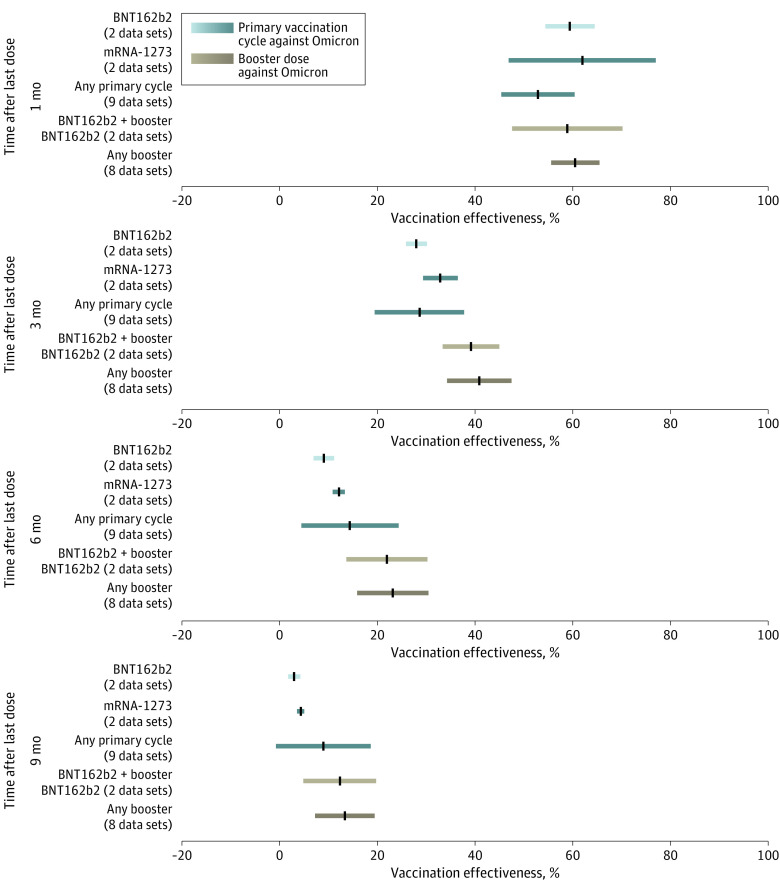
Effectiveness Over Time of Primary Vaccination Cycle and Booster Vaccination Against Omicron Symptomatic Disease Pooled estimates of vaccine effectiveness against symptomatic disease with Omicron across different vaccine products at 1, 3, 6, and 9 months from the administration of last dose. Vertical black lines indicate mean estimates; horizontal bars, 95% CIs.

**Figure 2.**
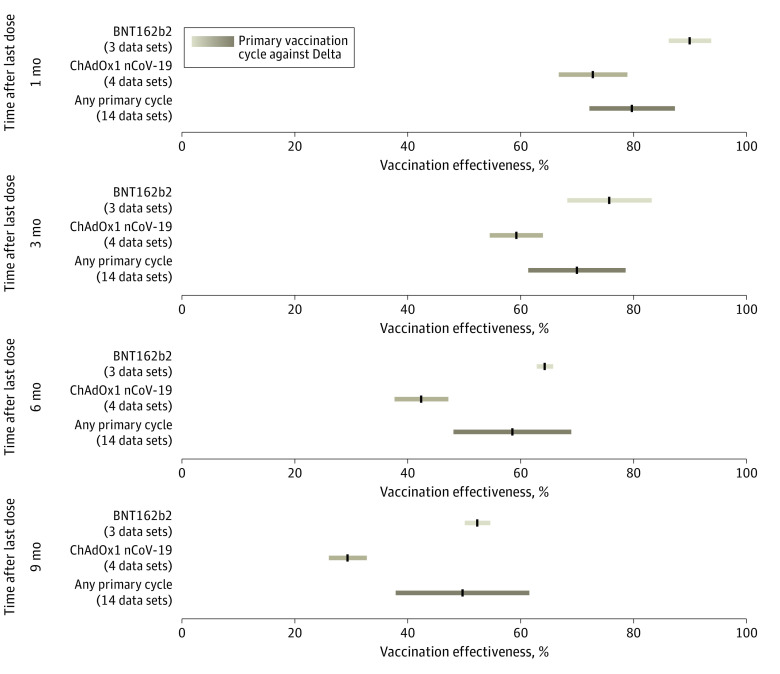
Effectiveness Over Time of Primary Vaccination Cycle Against Delta Symptomatic Disease Pooled estimates of vaccine effectiveness against symptomatic disease with Delta across different vaccine products at 1, 3, 6, and 9 months from the administration of primary vaccination cycle. Vertical black lines indicate mean estimates; horizontal bars, 95% CIs.

**Figure 3.**
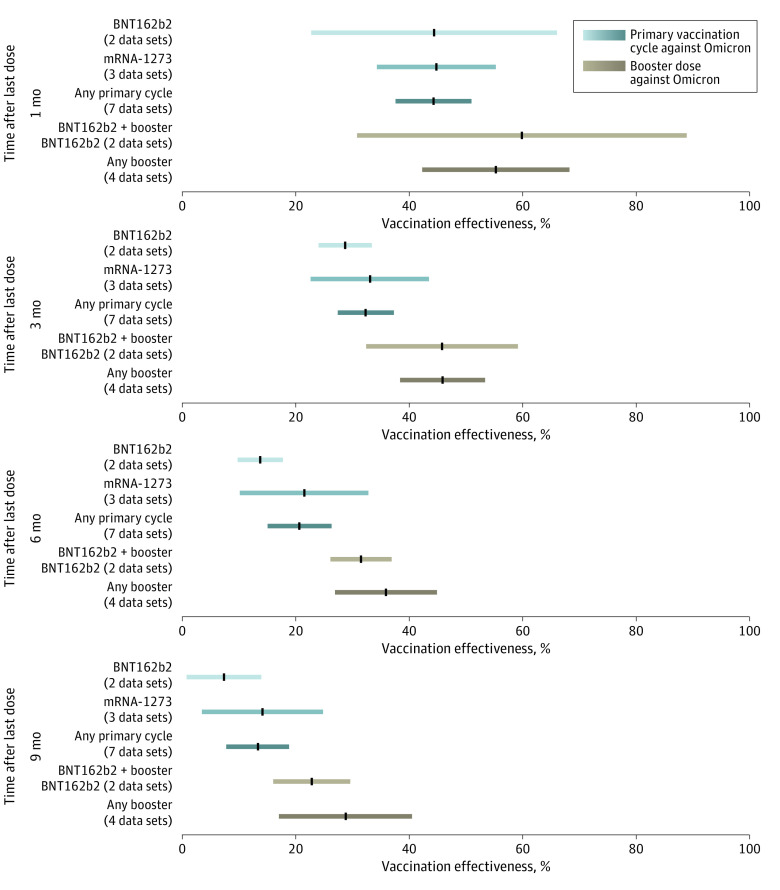
Effectiveness Over Time of Primary Vaccination Cycle and Booster Vaccination Against Any Omicron Laboratory-Confirmed Infection Pooled estimates of vaccine effectiveness against any laboratory-confirmed SARS-CoV-2 infection with Omicron across different vaccine products at 1, 3, 6, and 9 months from the administration of last dose. Vertical black lines indicate mean estimates; horizontal bars, 95% CIs.

**Figure 4.**
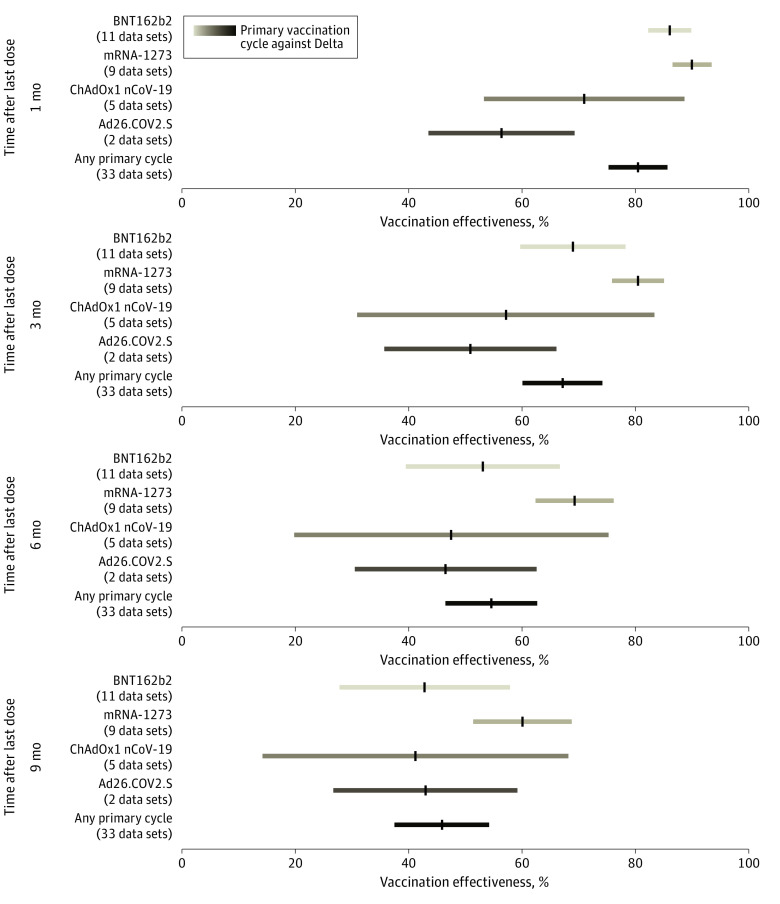
Effectiveness Over Time of Primary Vaccination Cycle Against Any Delta Laboratory-Confirmed Infection Pooled estimates of vaccine effectiveness against any laboratory-confirmed SARS-CoV-2 infection with Delta across different vaccine products at 1, 3, 6, and 9 months from the administration of primary vaccination cycle. Vertical black lines indicate mean estimates; horizontal bars, 95% CIs.

We identified 799 original articles and 149 reviews published in peer-reviewed journals, along with 35 preprints. Of these, we included 40 studies^9,10,11,12,13,14,15,16,17,18,19,20,21,22,23,24,25,26,27,28,29,30,31,32,33,34,35,36,37,38,39,40,41,42,43,44,45,46,47,48^ in our analysis (eFigure 1 in Supplement 1). Estimates of effectiveness over time are shown in Figure 1, Figure 2, Figure 3, and Figure 4 and eFigures 2 to 40 in Supplement 1.

Original estimates of VE reported in these articles were obtained as a result of test-negative case-control studies (n = 23),^9,10,11,12,13,14,15,32,33,34,35,36,37,38,39,40,41,42,43,44,46,47,48^ case-control studies (n = 1),^16^ or cohort studies (n = 16)^17,18,19,20,21,22,23,24,25,26,27,28,29,30,31,45^ assessing the difference in incidence of SARS-CoV-2 infection between vaccinated individuals and a certain reference group. A detailed description of different reference groups, end points, variants, and vaccine products is available in eAppendix 3 in Supplement 1. In sum, we considered 115 different time series of VE that include 454 data points for different vaccine products against Delta and Omicron variants over time. The study period, type of study, vaccine product, country, age group, number of doses, and the end point associated with the analyzed time series of VE for the 2 considered variants are summarized in eTables 1 and 2 in Supplement 1.

The adopted modeling approach well captured the temporal changes in the mean values of VE reported by the original studies (eFigures 2-5 in Supplement 1), allowing a comparison of VE associated with different vaccine products, variants, and number of administered doses over time and providing VE estimates beyond the final observation in the original studies (eg, at 9 months from vaccination). Model estimates of VE 14 days after the administration of the last dose (parameter *A*) and of the waning rate (*w*) are reported in eTables 7 and 8 in Supplement 1, along with the corresponding estimates obtained for the mean half-life of the vaccine-induced protection against the 2 considered end points.

### VE Against Symptomatic Disease

Pooled estimates of VE after any primary vaccination cycle against symptomatic disease after Omicron infection show a marked waning over time (Figure 1). We found that VE decreased from 52.8% (95% CI, 45.3%-60.3%) at 1 month after completion of any primary cycle to 14.3% (95% CI, 4.4%-24.3%) at 6 months to 8.9% (95% CI, −0.8% to 18.6%) at 9 months. Our estimates suggest that the initial VE could be different depending on the vaccine product, with higher VE found at 1 month from the second dose administration for mRNA-1273 (Moderna) (61.9%; 95% CI, 46.8%-76.9%) and BNT162b2 (Pfizer-BioNTech) (59.3%; 95% CI, 54.3%-64.4%) compared with ChAdOx1 nCoV-19 (AstraZeneca) (45.9%; 95% CI, 38.0%-54.1%) and CoronaVac (Sinovac) (32.4%; 95% CI, 23.7%-36.8%) (eFigure 6 in Supplement 1). We estimated the mean VE to be lower than 5% at 9 months after the administration of the second dose of BNT162b2, mRNA-1273, and ChAdOx1 nCoV-19 and 6 months after the second dose of CoronaVac. Our results suggest that the waning of VE against symptomatic disease after the primary vaccination cycle is remarkably higher for Omicron than for Delta (Figure 2 and eFigure 7 in Supplement 1). Pooled estimates show that VE against symptomatic disease with Delta was 79.6% (95% CI, 72.1%-87.2%) at 1 month after completion of the primary vaccination cycle, 58.5% (95% CI, 48.1%-68.9%) at 6 months, and 49.7% (95% CI, 37.9%-61.5%) at 9 months. This pattern is clearly shown by the estimated half-life of vaccine-induced immunity against symptomatic disease for the 2 variants, decreasing from 316 days (95% CI, 240-470 days) for Delta to 87 days (95% CI, 67-129 days) for Omicron.

The administration of a booster dose is associated with a restoration of VE against symptomatic disease after Omicron infection at levels comparable with those acquired right after the completion of the first cycle (Figure 1). At 3 months from the administration of the last dose, the VE of a booster dose against symptomatic disease was estimated to be 30.0% to 292.5% higher than the corresponding estimate for the primary cycle, with a high variability depending on the considered combination of vaccine products (eFigure 6 in Supplement 1). Nonetheless, pooled estimates show that the VE against symptomatic disease wanes at a rate comparable to that of the primary cycle, with VE decreasing from 60.4% (95% CI, 55.5%-65.4%) at 1 month after the administration of the booster dose to 13.3% (95% CI, 7.2%-19.4%) at 9 months. The estimated half-life of VE against Omicron symptomatic disease is 111 days (95% CI, 88-155 days). Consistent results were obtained when estimating VE by removing data points possibly affected by the initial ramp-up of vaccine protection (SA1) (eFigures 10, 12, and 16 in Supplement 1).

### VE Against Laboratory-Confirmed Infection

We estimated that the VE against laboratory-confirmed Omicron infection was 44.4% (95% CI, 37.7%-51.1%) at 1 month after completion of any primary vaccination cycle, 20.7% (95% CI, 15.1%-26.4%) at 6 months, and 13.4% (95% CI, 7.8%-18.9%) at 9 months (Figure 3 and eFigure 8 in Supplement 1). Similarly to what was found for symptomatic disease, a higher VE was found against laboratory-confirmed infection with Delta compared with Omicron (Figure 4 and eFigure 9 in Supplement 1). Pooled estimates of VE against laboratory-confirmed Delta infection after any primary cycle were 80.5% (95% CI, 75.3%-85.7%) at 1 month, 54.6% (95% CI, 46.5%-62.7%) at 6 months, and 45.9% (95% CI, 37.5%-54.2%) at 9 months. The estimated half-life of vaccine-induced immunity against laboratory-confirmed SARS-CoV-2 infection was 540 days (95% CI, 494-596 days) for Delta and 143 days (95% CI, 108-220 days) for Omicron.

The administration of a booster dose was associated with an initial VE against laboratory-confirmed infection with Omicron that was higher on average compared with the primary vaccination cycle (Figure 3) but with a marked variability at any time after booster administration. Pooled estimates of VE after the administration of a booster dose were 55.4% (95% CI, 42.4%-68.4%) at 1 month, 36.0% (95% CI, 27.0%-45.0%) at 6 months, and 28.9% (95% CI, 17.1%-40.6%) at 9 months. Consistent estimates were obtained in both sensitivity analyses (SA1 and SA2) (eFigures 11, 13, and 16 in Supplement 1).

### VE Against Laboratory-Confirmed Infection by Age Group

We found similar VE against laboratory-confirmed infection with Omicron in younger vs older age groups. In particular, if we compare individuals older than 60 years with individuals younger than 18 years, the obtained model estimates of VE are 39.2% (95% CI, 34.0%-44.4%) vs 38.7% (95% CI, 14.4%-63.1%) at 1 month, 13.6% (95% CI, 7.4%-20.8%) vs 13.1% (95% CI, 0.9%-25.3%) at 6 months, and 7.4% (95% CI, 2.7%-15.0%) vs 6.4% (95% CI, −0.5% to 13.2%) at 9 months (eFigure 14 in Supplement 1). No significant differences in pooled estimates of VE against laboratory-confirmed Delta infection were observed between the 2 age groups. A significantly lower VE was found for both age groups for Omicron compared with Delta.

## Discussion

In this study, we combined published evidence on the effectiveness of different vaccine products in preventing SARS-CoV-2 laboratory-confirmed infection and symptomatic disease to estimate the duration of vaccine-induced protection against these 2 end points for the Delta and Omicron variants. Results were used to quantify the level of vaccine-induced protection provided at any time from the administration of the last dose.

The performed analysis highlighted that the effectiveness of primary vaccination cycles against both symptomatic disease and laboratory-confirmed Omicron infection is initially lower and wanes more rapidly compared with what was observed for Delta. Consistent VE estimates were obtained for different age segments of the population. The administration of a booster dose was associated with VE levels comparable to those acquired right after the primary vaccination cycle. Our estimates are in line with the 125% increase of VE against Omicron, resulting from booster administration obtained by analyzing secondary attack rates in households.^57^ Nonetheless, our projections also showed that at 9 months from the administration of a booster, the mean VE against symptomatic disease and laboratory-confirmed Omicron infection might be lower than 20% and 30%, respectively.

Other systematic reviews and meta-analyses evaluated temporal changes in VE,^3,4,5^ providing evidence of the decrease over time of VE against laboratory-confirmed SARS-CoV-2 infection and symptomatic disease. Our results were consistent with those findings, corroborating the evidence of waning over time of VE against SARS-CoV-2 Delta infection and symptomatic disease and of a lower initial effectiveness and faster waning associated with Omicron with respect to previous variants.^4,5^ In particular, modeled VE estimates against Omicron laboratory-confirmed SARS-CoV-2 infection and symptomatic disease are in line with the results of the study by Wu et al^5^ against any Omicron SARS-CoV-2 infection. Compared with previous meta-analyses, the added value of our study is to provide better comparability of VE estimates coming from different studies. Indeed, the introduction of a functional form to model VE over time allows us to compare VE at any time point across different vaccine products, SARS-CoV-2 variants, number of doses, and epidemiologic end points and over relatively longer time horizons. We chose the exponential decay functional form because of its more widespread use^49,50^ and extensive applicability to epidemic models. Alternative functional forms proposed in the literature are the gamma distribution^52^ or a linear decay model.^53^ The exponential decay was able to effectively describe the temporal trend of VE of all selected studies (eFigures 2-5 in Supplement 1). Provided parameters for the initial VE and waning rate (eTables 7 and 8 in Supplement 1) can be readily used in transmission dynamic models in which the waning of immunity is usually assumed to follow an exponential distribution.

### Limitations

The presented results should be interpreted by considering the following limitations. Potential differences in VE across age groups were only partially assessed (eFigures 15 and 17-22 in Supplement 1). Original VE estimates against Omicron symptomatic disease^33,35,37,39,40,41,42,43,44,45,47^ and laboratory-confirmed infection^13,20,24,29,30,31,46^ refer to sublineages BA.1 and BA.2. Although a similar waning of vaccine immunity was found between these 2 sublineages,^58,59^ uncertainty remains on the effect of booster doses against more recent sublineages^60^ and the temporal patterns of VE associated with bivalent vaccines.^61^ In general, estimates of VE against laboratory-confirmed infection should also be cautiously interpreted. Laboratory-confirmed infections represent a mix of symptomatic and asymptomatic infections, where the latter usually have a different degree of underreporting due to preferential testing on symptomatic individuals. Estimates of VE against laboratory-confirmed infections in each study might depend on the relative contribution of asymptomatic ones. For this reason, we warn against drawing any conclusion about a differential duration of the protection against symptomatic disease and laboratory-confirmed infection. Different study designs were applied in original studies that were used to calibrate the developed statistical model of waning protection. This model includes the heterogeneous characteristics of the study population (eg, age, sex, and comorbidities) and the type of study (cohort vs case-control studies). Even with no explicit language restriction in the search, some published articles could have been ignored because of language issues, particularly with regard to CoronaVac and BBIBP-CorV vaccines, whose deployment was geographically more focalized. Our analysis does not investigate the possible differences in waning of vaccine protection for individuals who have never experienced a SARS-CoV-2 infection compared with previously infected individuals. Current evidence suggests that natural and hybrid immunity (vaccination followed by recovery from infection or recovery from infection followed by vaccination) might be more durable than vaccine-induced immunity.^62,63^ Given the large number of breakthrough infections caused by the emergence of the Omicron variant,^6,7,8^ comparing the duration of vaccine-induced and natural immunity remains an open issue. Finally, VE against severe disease, hospitalization, and mortality has been estimated to decrease more slowly compared with the end points considered in our analysis,^4,5^ granting a longer-lasting protection against severe outcomes.

## Conclusions

In this systematic review and meta-analysis, we estimated the duration of vaccine-induced protection against symptomatic and laboratory-confirmed infection against the Delta and Omicron variants. The estimates provided in this study can be instrumental to evaluate the susceptibility profile of populations with different levels of vaccinations, uptake by age, and vaccine products. This work could foster discussion on appropriate targets and timing for future vaccination programs. In principle, our results highlighted that a marked immune escape is associated with Omicron infection and symptomatic disease, with similar waning rates after the primary vaccination cycle and the booster dose. Boosters were found to be associated with a restoration of the vaccine protection against symptomatic disease to levels comparable to those estimated soon after completion of the primary cycle.

## Notes

Supplement 1. **eTable 1.** Study Period, Type of Study, Vaccine Product, Number of Doses, Age Group and Outcome Associated With the Analyzed Time Series of VE Against Omicron Variant **eTable 2.** Study Period, Type of Study, Vaccine Product, Number of Doses, Age Group and Outcome Associated With the Analyzed Time Series of VE Against Delta Variant **eTable 3.** Study Definitions for Symptomatic Disease **eTable 4.** Study Definitions for Laboratory-Confirmed Infection **eTable 5.** Quality Assessment for Case-Control Studies According to Newcastle-Ottawa Scale **eTable 6.** Quality Assessment for Cohort Studies According to Newcastle-Ottawa Scale **eTable 7.** Model Estimates of VE After the Ramp-Up (*A*), of the VE Waning Rate (*W*), and of the Half-Life of VE Against Symptomatic Disease With Delta and Omicron After Primary Vaccination Cycle and Booster Dose **eTable 8.** Model Estimates of VE After the Ramp-Up (*A*), of the VE Waning Rate (*W*), and of the Half-Life of VE Against Laboratory-Confirmed SARS-CoV-2 Infection With Delta and Omicron After Primary Vaccination Cycle and Booster Dose **eAppendix 1.** Data Extraction and Selection **eAppendix 2.** Model Details **eAppendix 3.** Characteristics of the Included Studies **eFigure 1.** Study Selection: Flowchart of the Selection of Studies Considered for the Performed Analysis **eFigure 2.** Effectiveness Over Time of Primary Vaccination Cycle and Booster Vaccination Against Omicron Symptomatic Disease **eFigure 3.** Effectiveness Over Time of Primary Vaccination Cycle and Booster Vaccination Against Any Omicron Laboratory-Confirmed Infection **eFigure 4.** Effectiveness Over Time of Primary Vaccination Cycle Against Delta Symptomatic Disease **eFigure 5.** Effectiveness Over Time of Primary Vaccination Cycle Against Any Delta Laboratory-Confirmed Infection **eFigure 6.** Effectiveness Over Time of Primary Vaccination Cycle and Booster Vaccination Against Omicron Symptomatic Disease for Single Time Series **eFigure 7.** Effectiveness Over Time of Primary Vaccination Cycle Against Delta Symptomatic Disease for Single Time Series **eFigure 8.** Effectiveness Over Time of Primary Vaccination Cycle and Booster Vaccination Against Any Omicron Laboratory-Confirmed Infection for Single Time Series **eFigure 9.** Effectiveness Over Time of Primary Vaccination Cycle Against Any Delta Laboratory-Confirmed Infection for Single Time Series **eFigure 10.** Effectiveness Over Time of Primary Vaccination Cycle and Booster Vaccination Against Omicron Symptomatic Disease According to Sensitivity Analysis SA1 **eFigure 11.** Effectiveness Over Time of Primary Vaccination Cycle and Booster Vaccination Against Any Omicron Laboratory-Confirmed Infection According to Sensitivity Analysis SA1 **eFigure 12.** Effectiveness Over Time of Primary Vaccination Cycle Against Delta Symptomatic Disease According to Sensitivity Analysis SA1 **eFigure 13.** Effectiveness Over Time of Primary Vaccination Cycle Against Any Delta Laboratory-Confirmed Infection According to Sensitivity Analysis SA1 **eFigure 14.** Effectiveness Over Time of Primary Vaccination Cycle Against Any Omicron and Delta Laboratory-Confirmed Infection for Young and Elderly Individuals **eFigure 15.** Effectiveness Over Time of Primary Vaccination Cycle Against Omicron and Delta Symptomatic Disease for Young and Elderly Individuals **eFigure 16.** Comparison of Vaccine Effectiveness Resulting From Main Analysis and Sensitivity Analyses **eFigure 17.** Effectiveness Over Time of Primary Vaccination Cycle Against Omicron Symptomatic Disease for Young Individuals **eFigure 18.** Effectiveness Over Time of Primary Vaccination Cycle Against Any Omicron Laboratory-Confirmed Infection for Young Individuals **eFigure 19.** Effectiveness Over Time of Primary Vaccination Cycle Against Delta Symptomatic Disease for Young Individuals **eFigure 20.** Effectiveness Over Time of Primary Vaccination Cycle Against Delta Symptomatic Disease for Elderly Individuals **eFigure 21.** Effectiveness Over Time of Primary Vaccination Cycle Against Any Delta Laboratory-Confirmed Infection for Young Individuals **eFigure 22.** Effectiveness Over Time of Primary Vaccination Cycle Against Any Delta Laboratory-Confirmed Infection for Elderly Individuals **eFigure 23.** Pooled Estimates of VE Against Symptomatic Disease With Omicron at 1, 3, 6, and 9 Months After Any Primary Vaccination Cycle **eFigure 24.** Pooled Estimates of VE Against Symptomatic Disease With Omicron at 1, 3, 6, and 9 Months After Two Doses of BNT162b2 **eFigure 25.** Pooled Estimates of VE Against Symptomatic Disease With Omicron at 1, 3, 6, and 9 Months After Two Doses of mRNA-1273 **eFigure 26.** Pooled Estimates of VE Against Any Laboratory-Confirmed Infection With Omicron at 1, 3, 6, and 9 Months After Any Primary Vaccination Cycle **eFigure 27.** Pooled Estimates of VE Against Any Laboratory-Confirmed Infection With Omicron at 1, 3, 6, and 9 Months After Two Doses of BNT162b2 **eFigure 28.** Pooled Estimates of VE Against Any Laboratory-Confirmed Infection With Omicron at 1, 3, 6, and 9 Months After Two Doses of mRNA- 1273 **eFigure 29.** Pooled Estimates of VE Against Symptomatic Disease With Omicron at 1, 3, 6, and 9 Months After Any Booster Dose **eFigure 30.** Pooled Estimates of VE Against Symptomatic Disease With Omicron at 1, 3, 6, and 9 Months After Three Doses of BNT162b2 **eFigure 31.** Pooled Estimates of VE Against Any Laboratory-Confirmed Infection With Omicron at 1, 3, 6, and 9 Months After Any Booster Dose **eFigure 32.** Pooled Estimates of VE Against Any Laboratory-Confirmed Infection With Omicron at 1, 3, 6, and 9 Months After Three Doses of BNT162b2 **eFigure 33.** Pooled Estimates of VE Against Symptomatic Disease With Delta at 1, 3, 6, and 9 Months After Any Primary Vaccination Cycle **eFigure 34.** Pooled Estimates of VE Against Symptomatic Disease With Delta at 1, 3, 6, and 9 Months After Two Doses of BNT162b2 **eFigure 35.** Pooled Estimates of VE Against Symptomatic Disease With Delta at 1, 3, 6, and 9 Months After Two Doses of ChAdOx1 nCoV-19 **eFigure 36.** Pooled Estimates of VE Against Any Laboratory-Confirmed Infection With Delta at 1, 3, 6, and 9 Months After Any Primary Vaccination Cycle **eFigure 37.** Pooled Estimates of VE Against Any Laboratory-Confirmed Infection With Delta at 1, 3, 6, and 9 Months After Two Doses of BNT162b2 **eFigure 38.** Pooled Estimates of VE Against Any Laboratory-Confirmed Infection With Delta at 1, 3, 6, and 9 Months After Two Doses of mRNA-1273 **eFigure 39.** Pooled Estimates of VE Against Any Laboratory-Confirmed Infection With Delta at 1, 3, 6, and 9 Months After Two Doses of ChAdOx1 nCoV-19 **eFigure 40.** Pooled Estimates of VE Against Any Laboratory-Confirmed Infection With Delta at 1, 3, 6, and 9 Months After One Dose of Ad26.COV2.S

Supplement 2.
Data Sharing Statement
