# Supplementary material for: Evaluation of Waning of SARS-CoV-2 Vaccine–Induced Immunity: A Systematic Review and Meta-analysis
Source: JAMA Netw Open. 2023 May 3;6(5):e2310650. doi: 10.1001/jamanetworkopen.2023.10650 (PMC10157431; doi:10.1001/jamanetworkopen.2023.10650)
Supplement: Supplement 1. — eTable 1. Study Period, Type of Study, Vaccine Product, Number of Doses, Age Group and Outcome Associated With the Analyzed Time Series of VE Against Omicron Variant eTable 2. Study Period, Type of Study, Vaccine Product, Number of Doses, Age Group and Outcome Associated With the Analyzed Time Series of VE Against Delta Variant eTable 3. Study Definitions for Symptomatic Disease eTable 4. Study Definitions for Laboratory-Confirmed Infection eTable 5. Quality Assessment for Case-Control Studies According to Newcastle-Ottawa Scale eTable 6. Quality Assessment for Cohort Studies According to Newcastle-Ottawa Scale eTable 7. Model Estimates of VE After the Ramp-Up (A), of the VE Waning Rate (W), and of the Half-Life of VE Against Symptomatic Disease With Delta and Omicron After Primary Vaccination Cycle and Booster Dose eTable 8. Model Estimates of VE After the Ramp-Up (A), of the VE Waning Rate (W), and of the Half-Life of VE Against Laboratory-Confirmed SARS-CoV-2 Infection With Delta and Omicron After Primary Vaccination Cycle and Booster Dose eAppendix 1. Data Extraction and Selection eAppendix 2. Model Details eAppendix 3. Characteristics of the Included Studies eFigure 1. Study Selection: Flowchart of the Selection of Studies Considered for the Performed Analysis eFigure 2. Effectiveness Over Time of Primary Vaccination Cycle and Booster Vaccination Against Omicron Symptomatic Disease eFigure 3. Effectiveness Over Time of Primary Vaccination Cycle and Booster Vaccination Against Any Omicron Laboratory-Confirmed Infection eFigure 4. Effectiveness Over Time of Primary Vaccination Cycle Against Delta Symptomatic Disease eFigure 5. Effectiveness Over Time of Primary Vaccination Cycle Against Any Delta Laboratory-Confirmed Infection eFigure 6. Effectiveness Over Time of Primary Vaccination Cycle and Booster Vaccination Against Omicron Symptomatic Disease for Single Time Series eFigure 7. Effectiveness Over Time of Primary Vaccination Cycle Against Delta Symptomatic Dis [file jamanetwopen-e2310650-s001.pdf]

Supplementary Online Content

Menegale F, Manica M, Zardini A, et al. Evaluation of waning of SARS-CoV-2 vaccine–induced immunity: a systematic review and meta-analysis. *JAMA Netw Open*. 2023;6(5):e2310650.
doi:10.1001/jamanetworkopen.2023.10650

eTable 1. Study Period, Type of Study, Vaccine Product, Number of Doses, Age Group and Outcome Associated With the Analyzed Time Series of VE Against Omicron Variant
eTable 2. Study Period, Type of Study, Vaccine Product, Number of Doses, Age Group and Outcome Associated With the Analyzed Time Series of VE Against Delta Variant
eTable 3. Study Definitions for Symptomatic Disease
eTable 4. Study Definitions for Laboratory-Confirmed Infection
eTable 5. Quality Assessment for Case-Control Studies According to Newcastle-Ottawa Scale
eTable 6. Quality Assessment for Cohort Studies According to Newcastle-Ottawa Scale
eTable 7. Model Estimates of VE After the Ramp-Up (A), of the VE Waning Rate (W), and of the Half-Life of VE Against Symptomatic Disease With Delta and Omicron After Primary Vaccination Cycle and Booster Dose
eTable 8. Model Estimates of VE After the Ramp-Up (A), of the VE Waning Rate (W), and of the Half-Life of VE Against Laboratory-Confirmed SARS-CoV-2 Infection With Delta and Omicron After Primary Vaccination Cycle and Booster Dose
eAppendix 1. Data Extraction and Selection
eAppendix 2. Model Details
eAppendix 3. Characteristics of the Included Studies
eFigure 1. Study Selection: Flowchart of the Selection of Studies Considered for the Performed Analysis
eFigure 2. Effectiveness Over Time of Primary Vaccination Cycle and Booster Vaccination Against Omicron Symptomatic Disease
eFigure 3. Effectiveness Over Time of Primary Vaccination Cycle and Booster Vaccination Against Any Omicron Laboratory-Confirmed Infection
eFigure 4. Effectiveness Over Time of Primary Vaccination Cycle Against Delta Symptomatic Disease
eFigure 5. Effectiveness Over Time of Primary Vaccination Cycle Against Any Delta Laboratory-Confirmed Infection
eFigure 6. Effectiveness Over Time of Primary Vaccination Cycle and Booster Vaccination Against Omicron Symptomatic Disease for Single Time Series
eFigure 7. Effectiveness Over Time of Primary Vaccination Cycle Against Delta Symptomatic Disease for Single Time Series
eFigure 8. Effectiveness Over Time of Primary Vaccination Cycle and Booster Vaccination Against Any Omicron Laboratory-Confirmed Infection for Single Time Series
eFigure 9. Effectiveness Over Time of Primary Vaccination Cycle Against Any Delta Laboratory-Confirmed Infection for Single Time Series
eFigure 10. Effectiveness Over Time of Primary Vaccination Cycle and Booster Vaccination Against Omicron Symptomatic Disease According to Sensitivity Analysis SA1
eFigure 11. Effectiveness Over Time of Primary Vaccination Cycle and Booster Vaccination Against Any Omicron Laboratory-Confirmed Infection According to Sensitivity Analysis SA1
eFigure 12. Effectiveness Over Time of Primary Vaccination Cycle Against Delta Symptomatic Disease According to Sensitivity Analysis SA1
eFigure 13. Effectiveness Over Time of Primary Vaccination Cycle Against Any Delta Laboratory-Confirmed Infection According to Sensitivity Analysis SA1
eFigure 14. Effectiveness Over Time of Primary Vaccination Cycle Against Any Omicron and Delta Laboratory-Confirmed Infection for Young and Elderly Individuals
eFigure 15. Effectiveness Over Time of Primary Vaccination Cycle Against Omicron and Delta Symptomatic Disease for Young and Elderly Individuals
eFigure 16. Comparison of Vaccine Effectiveness Resulting From Main Analysis and Sensitivity Analyses
eFigure 17. Effectiveness Over Time of Primary Vaccination Cycle Against Omicron Symptomatic Disease for Young Individuals

© 2023 Menegale F et al. *JAMA Network Open.*

eFigure 18. Effectiveness Over Time of Primary Vaccination Cycle Against Any Omicron Laboratory-Confirmed Infection for Young Individuals
eFigure 19. Effectiveness Over Time of Primary Vaccination Cycle Against Delta Symptomatic Disease for Young Individuals
eFigure 20. Effectiveness Over Time of Primary Vaccination Cycle Against Delta Symptomatic Disease for Elderly Individuals
eFigure 21. Effectiveness Over Time of Primary Vaccination Cycle Against Any Delta Laboratory-Confirmed Infection for Young Individuals
eFigure 22. Effectiveness Over Time of Primary Vaccination Cycle Against Any Delta Laboratory-Confirmed Infection for Elderly Individuals
eFigure 23. Pooled Estimates of VE Against Symptomatic Disease With Omicron at 1, 3, 6, and 9 Months After Any Primary Vaccination Cycle
eFigure 24. Pooled Estimates of VE Against Symptomatic Disease With Omicron at 1, 3, 6, and 9 Months After Two Doses of BNT162b2
eFigure 25. Pooled Estimates of VE Against Symptomatic Disease With Omicron at 1, 3, 6, and 9 Months After Two Doses of mRNA-1273
eFigure 26. Pooled Estimates of VE Against Any Laboratory-Confirmed Infection With Omicron at 1, 3, 6, and 9 Months After Any Primary Vaccination Cycle
eFigure 27. Pooled Estimates of VE Against Any Laboratory-Confirmed Infection With Omicron at 1, 3, 6, and 9 Months After Two Doses of BNT162b2
eFigure 28. Pooled Estimates of VE Against Any Laboratory-Confirmed Infection With Omicron at 1, 3, 6, and 9 Months After Two Doses of mRNA- 1273
eFigure 29. Pooled Estimates of VE Against Symptomatic Disease With Omicron at 1, 3, 6, and 9 Months After Any Booster Dose
eFigure 30. Pooled Estimates of VE Against Symptomatic Disease With Omicron at 1, 3, 6, and 9 Months After Three Doses of BNT162b2
eFigure 31. Pooled Estimates of VE Against Any Laboratory-Confirmed Infection With Omicron at 1, 3, 6, and 9 Months After Any Booster Dose
eFigure 32. Pooled Estimates of VE Against Any Laboratory-Confirmed Infection With Omicron at 1, 3, 6, and 9 Months After Three Doses of BNT162b2
eFigure 33. Pooled Estimates of VE Against Symptomatic Disease With Delta at 1, 3, 6, and 9 Months After Any Primary Vaccination Cycle
eFigure 34. Pooled Estimates of VE Against Symptomatic Disease With Delta at 1, 3, 6, and 9 Months After Two Doses of BNT162b2
eFigure 35. Pooled Estimates of VE Against Symptomatic Disease With Delta at 1, 3, 6, and 9 Months After Two Doses of ChAdOx1 nCoV-19
eFigure 36. Pooled Estimates of VE Against Any Laboratory-Confirmed Infection With Delta at 1, 3, 6, and 9 Months After Any Primary Vaccination Cycle
eFigure 37. Pooled Estimates of VE Against Any Laboratory-Confirmed Infection With Delta at 1, 3, 6, and 9 Months After Two Doses of BNT162b2
eFigure 38. Pooled Estimates of VE Against Any Laboratory-Confirmed Infection With Delta at 1, 3, 6, and 9 Months After Two Doses of mRNA-1273
eFigure 39. Pooled Estimates of VE Against Any Laboratory-Confirmed Infection With Delta at 1, 3, 6, and 9 Months After Two Doses of ChAdOx1 nCoV-19
eFigure 40. Pooled Estimates of VE Against Any Laboratory-Confirmed Infection With Delta at 1, 3, 6, and 9 Months After One Dose of Ad26.COV2.S

This supplementary material has been provided by the authors to give readers additional information about their work.

© 2023 Menegale F et al. *JAMA Network Open.*

eTable 1. Study Period, Type of Study, Vaccine Product, Number of Doses, Age Group and Outcome Associated With the Analyzed Time Series of VE Against Omicron Variant.

Type of vaccine	Considered period	Endpoint	Type of study	Identified reference group	N. of considered original VE estimates	Timespan of considered original VE estimates	Age groups	Country	Reference
BNT162b2 (2 doses)	Dec 7, 2021 - Feb 13, 2022	Laboratory-confirmed SARS-CoV-2 infection	Cohort study	Unvaccinated	2	14 - 135 days after 2nd dose	≥5 years	Czech Republic	Šmíd et al,[20] 2022
BNT162b2 (2 doses)	Dec 28, 2021 - Feb 15, 2022	Laboratory-confirmed SARS-CoV-2 infection	Cohort study	Unvaccinated	4	14 - 120 days after 2nd dose	≥12 years	Denmark	Hansen et al,[30] 2022
BNT162b2 (2 doses)	Jan 17, 2022 - Apr 13, 2022	Laboratory-confirmed SARS-CoV-2 infection	Cohort study	Unvaccinated	3	14 - 84 days after 2nd dose	5 - 11 years	Italy	Sacco et al,[31] 2022
BNT162b2 (2 doses)	Aug 25, 2021 - Jan 16, 2022	Laboratory-confirmed SARS-CoV-2 infection	Cohort study	Unvaccinated	2	7 - 63 days after 2nd dose	16 - 17 years	Norway	Veneti et al,[29] 2022
mRNA-1273 (2 doses)	Dec 7, 2021 - Feb 13, 2022	Laboratory-confirmed SARS-CoV-2 infection	Cohort study	Unvaccinated	2	14 - 135 days after 2nd dose	≥5 years	Czech Republic	Šmíd et al,[20] 2022
mRNA-1273 (2 doses)	Dec 6, 2021 - Dec 31, 2021	Laboratory-confirmed SARS-CoV-2 infection	Test-negative case-control	Unvaccinated	3	14 - 270 days after 2nd dose	≥18 years	US	Tseng et al,[13] 2022
mRNA-1273 (2 doses)	Dec 28, 2021 - Feb 15, 2022	Laboratory-confirmed SARS-CoV-2 infection	Cohort study	Unvaccinated	4	14 - 120 days after 2nd dose	≥12 years	Denmark	Hansen et al,[30] 2022
Ad26.COV2.S (1 dose)	Dec 7, 2021 - Feb 13, 2022	Laboratory-confirmed SARS-CoV-2 infection	Cohort study	Unvaccinated	2	14 - 135 days after 2nd dose	≥5 years	Czech Republic	Šmíd et al,[20] 2022
Unspecified/mixed mRNA products (BNT162b2 or mRNA-1273, 2 doses)	Dec 1, 2021 – Feb 25, 2022	Laboratory-confirmed SARS-CoV-2 infection	Cohort study	Unvaccinated	4	14 - 120 days after 2nd dose	12 - 59 years	Denmark	Gram et al,[24] 2022
Unspecified/mixed mRNA products (BNT162b2 or mRNA-1273, 2 doses)	Dec 1, 2021 – Feb 25, 2022	Laboratory-confirmed SARS-CoV-2 infection	Cohort study	Unvaccinated	4	14 – 120 days after 2nd dose	≥60 years	Denmark	Gram et al,[24] 2022
BNT162b2 (2 doses)	Nov 27, 2021 - Jan 12, 2022	Symptomatic disease	Test-negative case-control	Unvaccinated	5	2 - 24 weeks after 2nd dose	≥18 years	UK	Andrews et al[33], 2022
BNT162b2 (2 doses)	Dec 23, 2021 - Feb 2, 2022	Symptomatic disease	Test-negative case-control	Unvaccinated	7	0 - 210 days after 2nd dose	All ages	Qatar	Chemaitelly et al,[42] 2022
BNT162b2 (2 doses)	Dec 21, 2021 - Apr 19, 2022	Symptomatic disease	Test-negative case-control	Unvaccinated	6	14 - 98 days after 2nd dose	12 - 17 years	Scotland, UK	Florentino et al,[39] 2022
BNT162b2 (2 doses)	Jan 1, 2022 - Apr 19, 2022	Symptomatic disease	Test-negative case-control	Unvaccinated	6	14 - 98 days after 2nd dose	12 - 17 years	Brazil	Florentino et al,[39] 2022
BNT162b2 (2 doses)	Sep 13, 2021 - Jan 12, 2022	Symptomatic disease	Test-negative case-control	Unvaccinated	2	14 - 69 days after 2nd dose	12 - 18 years	UK	Powell et al,[40] 2022
BNT162b2 (2 doses)	Nov 22, 2021 - Mar 6, 2022	Symptomatic disease	Test-negative case-control	Unvaccinated	3	7 - 180 days after 2nd dose	16 - 17 years	Canada	Buchan et al,[41] 2022
BNT162b2 (2 doses)	Aug 6, 2021 - Mar 1, 2022	Symptomatic disease	Cohort study	Unvaccinated	3	2 - 13 weeks after 2nd dose	12 - 17 years	Scotland, UK	Rudan et al,[45] 2022
mRNA-1273 (2 doses)	Nov 27, 2021 - Jan 12, 2022	Symptomatic disease	Test-negative case-control	Unvaccinated	5	2 - 24 weeks after 2nd dose	≥18 years	UK	Andrews et al,[33] 2022

© 2023 Menegale F et al. *JAMA Network Open.*

Type of vaccine	Considered period	Endpoint	Type of study	Identified reference group	N. of considered original VE estimates	Timespan of considered original VE estimates	Age groups	Country	Reference
mRNA-1273 (2 doses)	Dec 23, 2021 - Feb 2, 2022	Symptomatic disease	Test-negative case-control	Unvaccinated	2	0 - 180 days after 2nd dose	All ages	Qatar	Chemaitelly et al,[42] 2022
ChAdOx1 nCoV-19 (2 doses)	Nov 27, 2021 - Jan 12, 2022	Symptomatic disease	Test-negative case-control	Unvaccinated	5	2 - 24 weeks after 2nd dose	≥18 years	UK	Andrews et al,[33] 2022
CoronaVac (2 doses)	Dec 25, 2021 – Apr 22, 2022	Symptomatic disease	Test-negative case-control	Unvaccinated	2	14 - 180 days after 2nd dose	≥18 years	Brazil	Ranzani et al,[35] 2022
Unspecified/mixed mRNA products (BNT162b2 or mRNA-1273, 2 doses)	Dec 6, 2021 - Dec 26, 2021	Symptomatic disease	Test-negative case-control	Unvaccinated	4	7 - 239 days after 2nd dose	≥18 years	Canada	Buchan et al,[37] 2022
Unspecified/mixed products (BBV152, ChAdOx1 nCoV-19, Gam-COVID-Vac, 2 doses)	Dec 1, 2021 - Feb 25, 2022	Symptomatic disease	Test-negative case-control	Unvaccinated	3	14 - 180 days after 2nd dose	≥18 years	India	Malhotra et al,[43] 2022
Unspecified/mixed products (BNT162b2, mRNA-1273, other products, 2 doses)	Jan 1, 2022 - Mar 31, 2022	Symptomatic disease	Test-negative case-control	Unvaccinated	2	14 - 180 days after 2nd dose	≥20 years	Japan	Arashiro et al,[44] 2022
BNT162b2 (2 doses) + BNT162b2 (booster)	Dec 20, 2021 - Apr 5, 2022	Laboratory-confirmed SARS-CoV-2 infection	Test-negative case-control	Unvaccinated	3	0 - 16 weeks after 3rd dose	≥18 years	US	Richterman et al,[46] 2022
	Dec 28, 2021 - Feb 15, 2022	Laboratory-confirmed SARS-CoV-2 infection	Cohort study	Unvaccinated	4	14 - 120 days after 2nd dose	≥12 years	Denmark	Hansen et al,[30] 2022
mRNA-1273 (2 doses) + mRNA-1273 (booster)	Dec 28, 2021 - Feb 15, 2022	Laboratory-confirmed SARS-CoV-2 infection	Cohort study	Unvaccinated	4	14 - 120 days after 2nd dose	≥12 years	Denmark	Hansen et al,[30] 2022
Unspecified/mixed mRNA products (Any mRNA vaccine, 3 doses)	Dec 1, 2021 – Feb 25, 2022	Laboratory-confirmed SARS-CoV-2 infection	Cohort study	Unvaccinated	4	14 - 120 days after 2nd dose	12 - 59 years	Denmark	Gram et al,[24] 2022
Unspecified/mixed mRNA products (Any mRNA vaccine, 3 doses)	Dec 1, 2021 – Feb 25, 2022	Laboratory-confirmed SARS-CoV-2 infection	Cohort study	Unvaccinated	4	14 - 120 days after 2nd dose	≥60 years	Denmark	Gram et al,[24] 2022
BNT162b2 (2 doses) + BNT162b2 (booster)	Nov 27, 2021 - Jan 12, 2022	Symptomatic disease	Test-negative case-control	Unvaccinated	2	2 - 9 weeks after 3rd dose	≥18 years	UK	Andrews et al,[33] 2022
BNT162b2 (2 doses) + BNT162b2 (booster)	Dec 23, 2021 - Feb 2, 2022	Symptomatic disease	Test-negative case-control	Unvaccinated	5	14 - 84 days after 2nd dose	All ages	Qatar	Chemaitelly et al,[42] 2022
BNT162b2 (2 doses) + mRNA-1273 (booster)	Nov 27, 2021 - Jan 12, 2022	Symptomatic disease	Test-negative case-control	Unvaccinated	2	2 - 9 weeks after 3rd dose	≥18 years	UK	Andrews et al,[33] 2022
mRNA-1273 (2 doses) + mRNA-1273 (booster)	Dec 23, 2021 - Feb 2, 2022	Symptomatic disease	Test-negative case-control	Unvaccinated	2	14 - 35 days after 2nd dose	All ages	Qatar	Chemaitelly et al,[42] 2022
ChAdOx1 nCoV-19 (2 doses) + BNT162b2 (booster)	Nov 27, 2021 - Jan 12, 2022	Symptomatic disease	Test-negative case-control	Unvaccinated	2	2 - 9 weeks after 3rd dose	≥18 years	UK	Andrews et al,[33] 2022
ChAdOx1 nCoV-19 (2 doses) + mRNA-1273 (booster)	Nov 27, 2021 - Jan 12, 2022	Symptomatic disease	Test-negative case-control	Unvaccinated	2	2 - 9 weeks after 3rd dose	≥18 years	UK	Andrews et al,[33] 2022
ChAdOx1 nCoV-19 (2 doses) + ChAdOx1 nCoV-19 (booster)	Nov 27, 2021 - Jan 12, 2022	Symptomatic disease	Test-negative case-control	Unvaccinated	2	2 - 9 weeks after 3rd dose	≥18 years	UK	Andrews et al,[33] 2022
CoronaVac (2 doses) +	Jan 01, 2022 - Apr 17, 2022	Symptomatic disease	Test-negative	Unvaccinated	4	14 - 120 days after 3rd dose	≥18 years	Brazil	Cerqueira-Silva et al,[47] 2022

© 2023 Menegale F et al. *JAMA Network Open.*

Type of vaccine	Considered period	Endpoint	Type of study	Identified reference group	N. of considered original VE estimates	Timespan of considered original VE estimates	Age groups	Country	Reference
BNT162b2 (booster)			case-control						
CoronaVac (2 doses) + BNT162b2 (booster)	Jan 01, 2022 - Apr 17, 2022	Symptomatic disease	Test-negative case-control	Unvaccinated	4	14 - 120 days after 3rd dose	≥80 years	Brazil	Cerqueira-Silva et al,[47] 2022

eTable 2. Study Period, Type of Study, Vaccine Product, Number of Doses, Age Group and Outcome Associated With the Analyzed Time Series of VE Against Delta Variant.

Type of vaccine	Considered period	Endpoint	Type of study	Identified reference group	N. of considered original VE estimates	Timespan of considered original VE estimates	Age groups	Country	Reference
BNT162b2 (2 doses)	Dec 14, 2020 - Aug 8, 2021	Laboratory-confirmed SARS-CoV-2 infection	Cohort study	Unvaccinated	3	14 - 126 days after 2nd dose	≥12 years	US	Tartof et al,[17] 2021
BNT162b2 (2 doses)	Jan 1, 2021-Sep 5, 2021	Laboratory-confirmed SARS-CoV-2 infection	Test-negative case-control	Unvaccinated	5	1 - 6 months after 2nd dose	≥12 years	Qatar	Chemaitelly et al,[9] 2021
BNT162b2 (2 doses)	May 30, 2021- Nov 27, 2021	Laboratory-confirmed SARS-CoV-2 infection	Test-negative case-control	Unvaccinated	10	14 - 279 days after 2nd dose	≥18 years	British Columbia, Canada	Skowronski et al,[10] 2022
BNT162b2 (2 doses)	May 30, 2021- Nov 27, 2021	Laboratory-confirmed SARS-CoV-2 infection	Test-negative case-control	Unvaccinated	8	14 - 223 days after 2nd dose	≥18 years	Quebec, Canada	Skowronski et al,[10] 2022
BNT162b2 (2 doses)	May 23, 2021 - Nov 23, 2021	Laboratory-confirmed SARS-CoV-2 infection	Cohort study	Unvaccinated	8	1 - 8 months after 2nd dose	≥15 years	UK	Menni et al,[18] 2022
BNT162b2 (2 doses)	Mar 1, 2021 - Oct 31, 2021	Laboratory-confirmed SARS-CoV-2 infection	Test-negative case-control	Unvaccinated	7	14 - 111 days after 2nd dose	≥18 years	Malaysia	Lim et al,[11] 2022
BNT162b2 (2 doses)	Jul 15, 2021 – Nov 30, 2021	Laboratory-confirmed SARS-CoV-2 infection	Cohort study	Unvaccinated	4	2 – 33 weeks after 2nd dose	≥18 years	Norway	Starrfelt et al,[19] 2022
BNT162b2 (2 doses)	Dec 7,2021 - Feb 13, 2022	Laboratory-confirmed SARS-CoV-2 infection	Cohort study	Unvaccinated	2	14 - 135 days after 2nd dose	≥5 years	Czech Republic	Šmíd et al,[20] 2022
BNT162b2 (2 doses)	Sep 13, 2021 - Dec 31, 2021	Laboratory-confirmed SARS-CoV-2 infection	Cohort study	Unvaccinated	3	14 - 120 days after 2nd dose	18-64 years	Hungary	Vokó et al,[25] 2022
BNT162b2 (2 doses)	Sep 13, 2021 - Dec 31, 2021	Laboratory-confirmed SARS-CoV-2 infection	Cohort study	Unvaccinated	3	14 - 120 days after 2nd dose	65-100 years	Hungary	Vokó et al,[25] 2022
BNT162b2 (2 doses)	Jul 23, 2021 - Dec 15, 2021	Laboratory-confirmed SARS-CoV-2 infection	Cohort study	Unvaccinated	3	3 – 14 weeks after 2nd dose	18-39 years	UK	Horne et al,[26] 2022
BNT162b2 (2 doses)	May 18, 2021 - Dec 15, 2021	Laboratory-confirmed SARS-CoV-2 infection	Cohort study	Unvaccinated	4	3 – 18 weeks after 2nd dose	40-64 years	UK	Horne et al,[26] 2022
BNT162b2 (2 doses)	Mar 15, 2021 - Nov 30, 2021	Laboratory-confirmed SARS-CoV-2 infection	Cohort study	Unvaccinated	4	11 – 26 weeks after 2nd dose	≥65 years	UK	Horne et al,[26] 2022
BNT162b2 (2 doses)	Jul 11, 2021 – Jul 31, 2021	Laboratory-confirmed SARS-CoV-2 infection	Cohort study	Unvaccinated	7	41 - 196 days after 2nd dose	16-39 years	Israel	Goldberg et al,[27] 2021
BNT162b2 (2 doses)	Jul 11, 2021 – Jul 31, 2021	Laboratory-confirmed SARS-CoV-2 infection	Cohort study	Unvaccinated	7	41 - 196 days after 2nd dose	40-59 years	Israel	Goldberg et al,[27] 2021

© 2023 Menegale F et al. *JAMA Network Open.*

Type of vaccine	Considered period	Endpoint	Type of study	Identified reference group	N. of considered original VE estimates	Timespan of considered original VE estimates	Age groups	Country	Reference
BNT162b2 (2 doses)	Jul 11, 2021 – Jul 31, 2021	Laboratory-confirmed SARS-CoV-2 infection	Cohort study	Unvaccinated	7	41 - 196 days after 2nd dose	≥60 years	Israel	Goldberg et al,[27] 2021
BNT162b2 (2 doses)	Sep 1, 2021 - Dec 31, 2021	Laboratory-confirmed SARS-CoV-2 infection	Test-negative case-control	Unvaccinated	2	1 - 91 days after 2nd dose	12 - 17 years	Malaysia	Husin et al,[15] 2022
BNT162b2 (2 doses)	Jun 15, 2021 – Dec 8, 2021	Laboratory-confirmed SARS-CoV-2 infection	Case-control study	Unvaccinated	3	14 - 180 days after 2nd dose	12 - 16 years	Israel	Prunas et al,[16] 2022
BNT162b2 (2 doses)	Aug 8, 2021 - Dec 4, 2021	Laboratory-confirmed SARS-CoV-2 infection	Cohort study	Unvaccinated	6	1 - 6 months after 2nd dose	18 - 24 years	US	Rennert et al,[28] 2022
BNT162b2 (2 doses)	Aug 25, 2021 - Jan 16, 2022	Laboratory-confirmed SARS-CoV-2 infection	Cohort study	Unvaccinated	2	7 - 63 days after 2nd dose	16 - 17 years	Norway	Veneti et al,[29] 2022
mRNA-1273 (2 doses)	May 30, 2021- Nov 27, 2021	Laboratory-confirmed SARS-CoV-2 infection	Test-negative case-control	Unvaccinated	10	14 - 279 days after 2nd dose	≥18 years	British Columbia, Canada	Skowronski et al,[10] 2022
mRNA-1273 (2 doses)	May 30, 2021- Nov 27, 2021	Laboratory-confirmed SARS-CoV-2 infection	Test-negative case-control	Unvaccinated	8	14 - 223 days after 2nd dose	≥18 years	Quebec, Canada	Skowronski et al,[10] 2022
mRNA-1273 (2 doses)	May 23, 2021 - Nov 23, 2021	Laboratory-confirmed SARS-CoV-2 infection	Cohort study	Unvaccinated	5	1 - 5 months after 2nd dose	≥15 years	UK	Menni et al,[18] 2022
mRNA-1273 (2 doses)	Jul 15, 2021 – Nov 30, 2021	Laboratory-confirmed SARS-CoV-2 infection	Cohort study	Unvaccinated	4	2 – 33 weeks after 2nd dose	≥18 years	Norway	Starrfelt et al,[19] 2022
mRNA-1273 (2 doses)	Dec 7, 2021 - Feb 13, 2022	Laboratory-confirmed SARS-CoV-2 infection	Cohort study	Unvaccinated	2	14 - 135 days after 2nd dose	≥5 years	Czech Republic	Šmíd et al,[20] 2022
mRNA-1273 (2 doses)	Sep 13, 2021 - Dec 31, 2021	Laboratory-confirmed SARS-CoV-2 infection	Prospective cohort study	Unvaccinated	3	14 - 120 days after 2nd dose	18 - 64 years	Hungary	Vokó et al,[25] 2022
mRNA-1273 (2 doses)	Sep 13, 2021 - Dec 31, 2021	Laboratory-confirmed SARS-CoV-2 infection	Prospective cohort study	Unvaccinated	3	14 - 120 days after 2nd dose	65 - 100 years	Hungary	Vokó et al,[25] 2022
mRNA-1273 (2 doses)	Aug 8, 2021 - Dec 4, 2021	Laboratory-confirmed SARS-CoV-2 infection	Cohort study	Unvaccinated	6	1 - 6 months after 2nd dose	18 - 24 years	US	Rennert et al[28], 2022
mRNA-1273 (2 doses)	Mar 1, 2021 - Jul 27, 2021	Laboratory-confirmed SARS-CoV-2 infection	Test-negative case-control	Unvaccinated	5	14 - 180 days after 2nd dose	≥18 years	US	Bruxvoort et al,[12] 2021
mRNA-1273 (2 doses)	Jan 1, 2021 - Sep 30, 2021	Laboratory-confirmed SARS-CoV-2 infection	Cohort study	Unvaccinated	4	0 - 8 months after 2nd dose	≥18 years	US	Florea et al,[21] 2022
mRNA-1273 (2 doses)	Jan 1, 2021 - Sep 30, 2021	Laboratory-confirmed SARS-CoV-2 infection	Cohort study	Unvaccinated	4	0 - 8 months after 2nd dose	≥65 years	US	Florea et al,[21] 2022
mRNA-1273 (2 doses)	Dec 6, 2021 - Dec 31, 2021	Laboratory-confirmed SARS-CoV-2 infection	Test-negative case-control	Unvaccinated	3	14 - 270 days after 2nd dose	≥18 years	US	Tseng et al,[13] 2022
ChAdOx1 nCoV-19 (2 doses)	May 30, 2021- Nov 27, 2021	Laboratory-confirmed SARS-CoV-2 infection	Test-negative case-control	Unvaccinated	6	28 - 195 days after 2nd dose	≥18 years	British Columbia, Canada	Skowronski et al,[10] 2022
ChAdOx1 nCoV-19 (2 doses)	May 30, 2021- Nov 27, 2021	Laboratory-confirmed SARS-CoV-2 infection	Test-negative case-control	Unvaccinated	5	56 - 195 days after 2nd dose	≥18 years	Quebec, Canada	Skowronski et al,[10] 2022

© 2023 Menegale F et al. *JAMA Network Open.*

Type of vaccine	Considered period	Endpoint	Type of study	Identified reference group	N. of considered original VE estimates	Timespan of considered original VE estimates	Age groups	Country	Reference
ChAdOx1 nCoV-19 (2 doses)	May 23, 2021 - Nov 23, 2021	Laboratory-confirmed SARS-CoV-2 infection	Cohort study	Unvaccinated	6	1 - 6 months after 2nd dose	≥15 years	UK	Menni et al,[18] 2022
ChAdOx1 nCoV-19 (2 doses)	Sep 13, 2021 - Dec 31, 2021	Laboratory-confirmed SARS-CoV-2 infection	Cohort study	Unvaccinated	2	14 - 120 days after 2nd dose	18 - 64 years	Hungary	Vokó et al,[25] 2022
ChAdOx1 nCoV-19 (2 doses)	Sep 13, 2021 - Dec 31, 2021	Laboratory-confirmed SARS-CoV-2 infection	Cohort study	Unvaccinated	3	14 - 120 days after 2nd dose	65 - 100 years	Hungary	Vokó et al,[25] 2022
ChAdOx1 nCoV-19 (2 doses)	May 18, 2021 - Dec 15, 2021	Laboratory-confirmed SARS-CoV-2 infection	Cohort study	Unvaccinated	2	3 – 18 weeks after 2nd dose	40-64 years	UK	Horne et al,[26] 2022
ChAdOx1 nCoV-19 (2 doses)	Mar 15, 2021 - Nov 30, 2021	Laboratory-confirmed SARS-CoV-2 infection	Cohort study	Unvaccinated	2	11 – 26 weeks after 2nd dose	≥65 years	UK	Horne et al,[26] 2022
Ad26.COV2.S (1 dose)	Dec 7, 2021 - Feb 13, 2022	Laboratory-confirmed SARS-CoV-2 infection	Cohort study	Unvaccinated	2	14 - 135 days after 2nd dose	≥5 years	Czech Republic	Šmíd et al,[20] 2022
Ad26.COV2.S (1 dose)	Sep 13, 2021 - Dec 31, 2021	Laboratory-confirmed SARS-CoV-2 infection	Cohort study	Unvaccinated	3	14 - 120 days after 2nd dose	18 - 64 years	Hungary	Vokó et al,[25] 2022
BBIBP-CorV (2 doses)	Sep 13, 2021 - Dec 31, 2021	Laboratory-confirmed SARS-CoV-2 infection	Cohort study	Unvaccinated	2	14 - 120 days after 2nd dose	18-64 years	Hungary	Vokó et al,[25] 2022
BBIBP-CorV (2 doses)	Sep 13, 2021 - Dec 31, 2021	Laboratory-confirmed SARS-CoV-2 infection	Cohort study	Unvaccinated	3	14 - 120 days after 2nd dose	65-100 years	Hungary	Vokó et al,[25] 2022
Gam-COVID-Vac (2 doses)	Sep 13, 2021 - Dec 31, 2021	Laboratory-confirmed SARS-CoV-2 infection	Cohort study	Unvaccinated	3	14 - 120 days after 2nd dose	18-64 years	Hungary	Vokó et al,[25] 2022
Gam-COVID-Vac (2 doses)	Sep 13, 2021 - Dec 31, 2021	Laboratory-confirmed SARS-CoV-2 infection	Cohort study	Unvaccinated	2	14 - 120 days after 2nd dose	65-100 years	Hungary	Vokó et al,[25] 2022
Unspecified/mixed mRNA products (BNT162b2 or mRNA-1273, 2 doses)	Jul 15, 2021 – Nov 30, 2021	Laboratory-confirmed SARS-CoV-2 infection	Cohort study	Unvaccinated	4	2 – 33 weeks after 2nd dose	≥18 years	Norway	Starrfelt et al,[19] 2022
Unspecified/mixed products (BNT162b2, mRNA-1273, ChAdOx1 nCov-19, 2 doses)	Jul 15, 2021 – Nov 30, 2021	Laboratory-confirmed SARS-CoV-2 infection	Cohort study	Unvaccinated	4	2 – 33 weeks after 2nd dose	≥18 years	Norway	Starrfelt et al,[19] 2022
Unspecified/mixed products (BNT162b2, mRNA-1273, ChAdOx1 nCov-19, 2 doses)	Jul 15, 2021 – Nov 30, 2021	Laboratory-confirmed SARS-CoV-2 infection	Cohort study	Unvaccinated	4	2 – 33 weeks after 2nd dose	≥65 years	Norway	Starrfelt et al,[19] 2022
Unspecified/mixed mRNA products (BNT162b2 or mRNA-1273, 2 doses)	Jul 19, 2021 - Nov 7, 2021	Laboratory-confirmed SARS-CoV-2 infection	Cohort study	Partially vaccinated from less than 14 days since 1st dose	9	3 - 42 weeks after 2nd dose	≥16 years	Italy	Fabiani et al,[22] 2022
Unspecified/mixed mRNA products (BNT162b2 or mRNA-1273, 2 doses)	Jul 19, 2021 - Nov 7, 2021	Laboratory-confirmed SARS-CoV-2 infection	Cohort study	Partially vaccinated from less than 14 days since 1st dose	9	3 - 42 weeks after 2nd dose	≥80 years	Italy	Fabiani et al,[22] 2022
Unspecified/mixed products (BNT162b2, mRNA-1273, ChAdOx1 nCoV-19, Ad26.COV2.S, 2 doses)	Jul 19, 2021 - Dec 12, 2021	Laboratory-confirmed SARS-CoV-2 infection	Cohort study	Partially vaccinated from 4-10 days since 1st dose	3	3 - 26 weeks after 2nd dose	≥16 years	Italy	Fabiani et al,[23] 2022

© 2023 Menegale F et al. *JAMA Network Open.*

Type of vaccine	Considered period	Endpoint	Type of study	Identified reference group	N. of considered original VE estimates	Timespan of considered original VE estimates	Age groups	Country	Reference
Unspecified/mixed products (BNT162b2, mRNA-1273, ChAdOx1 nCoV-19, Ad26.COV2.S, 2 doses)	Jul 19, 2021 - Dec 12, 2021	Laboratory-confirmed SARS-CoV-2 infection	Cohort study	Partially vaccinated from 4-10 days since 1st dose	3	3 - 26 weeks after 2nd dose	≥80 years	Italy	Fabiani et al,[23] 2022
Unspecified/mixed mRNA products (BNT162b2 or mRNA-1273, 2 doses)	Jun 28, 2021 - Nov 21, 2021	Laboratory-confirmed SARS-CoV-2 infection	Test-negative case-control	Unvaccinated	4	7 – 240 days after 2nd dose	≥16 years	Canada	Chung et al,[14] 2022
Unspecified/mixed products (any ChAdOx1 nCov-19-containing schedule, including ChAdOx1 nCov-19/ChAdOx1 nCov-19, ChAdOx1 nCov-19/BNT162b2, and ChAdOx1 nCov-19/mRNA-1273, 2 doses)	Jun 28, 2021 - Nov 21, 2021	Laboratory-confirmed SARS-CoV-2 infection	Test-negative case-control	Unvaccinated	3	7 – 240 days after 2nd dose	≥16 years	Canada	Chung et al,[14] 2022
Unspecified/mixed mRNA products (BNT162b2 or mRNA-1273, 2 doses)	Jul 4, 2021 - Nov 20, 2021	Laboratory-confirmed SARS-CoV-2 infection	Cohort study	Unvaccinated	4	14 – 120 days after 2nd dose	12 - 59 years	Denmark	Gram et al,[24] 2022
Unspecified/mixed mRNA products (BNT162b2 or mRNA-1273, 2 doses)	Jul 4, 2021 - Nov 20, 2021	Laboratory-confirmed SARS-CoV-2 infection	Cohort study	Unvaccinated	4	14 – 120 days after 2nd dose	≥60 years	Denmark	Gram et al,[24] 2022
BNT162b2 (2 doses)	Apr 12, 2021 - Oct 1, 2021	Symptomatic disease	Test-negative case-control	Unvaccinated	3	2 - 19 weeks after 2nd dose	≥16 years	UK	Andrews et al,[32] 2022
BNT162b2 (2 doses)	Apr 12, 2021 - Oct 1, 2021	Symptomatic disease	Test-negative case-control	Unvaccinated	3	2 - 19 weeks after 2nd dose	≥65 years	UK	Andrews et al,[32] 2022
BNT162b2 (2 doses)	Nov 27, 2021 - Jan 12, 2022	Symptomatic disease	Test-negative case-control	Unvaccinated	5	2 - 24 weeks after 2nd dose	≥18 years	UK	Andrews et al,[33] 2022
BNT162b2 (2 doses)	Jul 01, 2021 - Aug 31, 2021	Symptomatic disease	Test-negative case-control	Unvaccinated	3	14 - 90 days after 2nd dose	30 - 59 years	Europe	Kissling et al,[38] 2022
BNT162b2 (2 doses)	Jul 01, 2021 - Aug 31, 2021	Symptomatic disease	Test-negative case-control	Unvaccinated	2	14 - 90 days after 2nd dose	≥60 years	Europe	Kissling et al,[38] 2022
BNT162b2 (2 doses)	Jun 15, 2021 – Dec 8, 2021	Symptomatic disease	Case-control study	Unvaccinated	3	14 - 180 days after 2nd dose	12 - 16 years	Israel	Prunas et al,[16] 2022
BNT162b2 (2 doses)	Sep 2, 2021 - Dec 31, 2021	Symptomatic disease	Test-negative case-control	Unvaccinated	4	14 - 69 days after 2nd dose	12 - 17 years	Brazil	Florentino et al,[39] 2022
BNT162b2 (2 doses)	Sep 13, 2021 - Jan 12, 2022	Symptomatic disease	Test-negative case-control	Unvaccinated	2	14 - 69 days after 2nd dose	12 - 18 years	UK	Powell et al,[40] 2022
BNT162b2 (2 doses)	Nov 22, 2021 - Mar 6, 2022	Symptomatic disease	Test-negative case-control	Unvaccinated	3	7 - 180 days after 2nd dose	16 - 17 years	Canada	Buchan et al,[41] 2022
mRNA-1273 (2 doses)	Nov 27, 2021 - Jan 12, 2022	Symptomatic disease	Test-negative case-control	Unvaccinated	5	2 - 24 weeks after 2nd dose	≥18 years	UK	Andrews et al,[33] 2022
ChAdOx1 nCoV-19 (2 doses)	Apr 12, 2021 - Oct 1, 2021	Symptomatic disease	Test-negative case-control	Unvaccinated	3	2 - 19 weeks after 2nd dose	≥16 years	UK	Andrews et al,[32] 2022

© 2023 Menegale F et al. *JAMA Network Open.*

Type of vaccine	Considered period	Endpoint	Type of study	Identified reference group	N. of considered original VE estimates	Timespan of considered original VE estimates	Age groups	Country	Reference
ChAdOx1 nCoV-19 (2 doses)	Apr 12, 2021 - Oct 1, 2021	Symptomatic disease	Test-negative case-control	Unvaccinated	3	2 - 19 weeks after 2nd dose	≥65 years	UK	Andrews et al,[32] 2022
ChAdOx1 nCoV-19 (2 doses)	Nov 27, 2021 - Jan 12, 2022	Symptomatic disease	Test-negative case-control	Unvaccinated	5	2 - 24 weeks after 2nd dose	≥18 years	UK	Andrews et al,[33] 2022
ChAdOx1 nCoV-19 (2 doses)	May 19, 2021 - Oct 25, 2021	Symptomatic disease	Test-negative case-control	Unvaccinated	10	2 - 21 weeks after 2nd dose	≥18 years	Scotland, UK	Katikireddi et al,[34] 2022
ChAdOx1 nCoV-19 (2 doses)	Jul 01, 2021 - Aug 31, 2021	Symptomatic disease	Test-negative case-control	Unvaccinated	3	14 - 90 days after 2nd dose	30 - 59 years	Europe	Kissling et al,[38] 2022
Ad26.COV2.S (1 dose)	Jul 01, 2021 - Aug 31, 2021	Symptomatic disease	Test-negative case-control	Unvaccinated	2	14 – 90 days after 2nd dose	30 - 59 years	Europe	Kissling et al,[38] 2022
CoronaVac (2 doses)	Sep 06, 2021 - Dec 14, 2021	Symptomatic disease	Test-negative case-control	Unvaccinated	2	14 - 180 days after 2nd dose	≥18 years	Brazil	Ranzani et al,[35] 2022
Unspecified/mixed mRNA products (BNT162b2 or mRNA-1273, 2 doses)	Jun 28, 2021 - Nov 21, 2021	Symptomatic disease	Test-negative case-control	Unvaccinated	4	7 – 240 days after 2nd dose	≥16 years	Canada	Chung et al,[14] 2022
Unspecified/mixed products (any ChAdOx1 nCov-19-containing schedule, including ChAdOx1 nCov-19/ChAdOx1 nCov-19, ChAdOx1 nCov-19/BNT162b2, and ChAdOx1 nCov-19/mRNA-1273, 2 doses)	Jun 28, 2021 - Nov 21, 2021	Symptomatic disease	Test-negative case-control	Unvaccinated	3	7 – 240 days after 2nd dose	≥16 years	Canada	Chung et al,[14] 2022
Unspecified/mixed products (BNT162b2, mRNA-1273, ChAdOx1nCoV-19, 2 doses)	Jan 1, 2021 -Dec 12, 2021	Symptomatic disease	Test-negative case-control	Unvaccinated	6	15 - 180 days after 2nd dose	≥50 years	France	Suarez Castillo et al,[36] 2022
Unspecified/mixed mRNA products (BNT162b2 or mRNA-1273, 2 doses)	Dec 6, 2021 - Dec 26, 2021	Symptomatic disease	Test-negative case-control	Unvaccinated	4	7 - 239 days after 2nd dose	≥18 years	Canada	Buchan et al,[37] 2022

eTable 3. Study Definitions for Symptomatic Disease.

Study	Symptomatic disease definition
Prunas et al,[16] 2022	RT-PCR positive test and exhibition of COVID-19-related symptoms.
Andrews et al,[32] 2022	PCR-confirmed SARS-CoV-2 infection with symptoms consistent with COVID-19.
Andrews et al,[33] 2022	PCR-confirmed SARS-CoV-2 infection with symptoms consistent with COVID-19.
Katikireddi et al,[34] 2022	RT-PCR positive test with symptoms consistent with COVID-19.
Ranzani et al,[35] 2022	Positive SARS-CoV-2 RT-PCR or rapid antigen testing associated with symptomatic illness.
Suarez Castillo et al,[36] 2022	Symptomatic positive individuals.
Buchan et al,[37] 2022	Positive RT-PCR test with at least one COVID-19–related symptom (self-reported or measured)
Kissling et al,[38] 2022	Positive RT-PCR test or rapid antigen test (RAT) associated with COVID-19 symptoms.
Florentino et al,[39] 2022	Symptomatic infection, confirmed by rapid antigen testing or RT-PCR in Brazil and only by RT-PCR in Scotland.
Powell et al,[40] 2022	PCR-confirmed SARS-CoV-2 infection with symptoms consistent with COVID-19.
Buchan et al,[41] 2022	Positive RT-PCR test with COVID-19–related symptoms.
Chemaitelly et al,[42] 2022	PCR positive swab collected because of clinical suspicion due to presence of symptoms compatible with a respiratory tract infection.
Malhotra et al,[43] 2022	SARS COV-2 confirmed by RT-PCR and CBNAAT laboratory test with the presence of any of the following symptoms- fever, rhinorrhea, sore throat, cough, chest pain, wheezing, difficulty in breathing, shortness of breath, anosmia, dysgeusia, fatigue, myalgia/ body aches, headache, abdominal pain, nausea/ vomiting, and diarrhea.

© 2023 Menegale F et al. *JAMA Network Open.*

Arashiro et al,[44] 2022	PCR positive test with any of the following symptom: fever ≥37.5°C, malaise, chills, joint pain, headache, runny nose, cough, sore throat, shortness of breath, gastrointestinal symptoms (vomiting, diarrhea, stomachache), and loss of taste/smell.
Rudan et al,[45] 2022	Symptomatic COVID-19 disease with RT-PCR test positivity for SARS-CoV-2 infection.
Cerqueira-Silva et al,[47] 2022	RT-PCR/ Lateral-flow test positive on individuals presenting COVID-19-like symptoms.

eTable 4. Study Definitions for Laboratory-Confirmed Infection.

**Study**	**Laboratory-confirmed infection**
Chemaitelly et al,[9] 2021	Reverse transcription polymerase chain reaction (RT-PCR) positive test.
Skowronski et al,[10] 2022	SARS-CoV-2 nucleic acid amplification test (NAAT) positive test.
Lim et al,[11] 2022	RT-PCR confirmation of infection with SARS-CoV-2 irrespective of clinical signs or symptoms.
Bruxvoort et al,[12] 2021	Positive test for SARS-CoV-2 with or without symptoms.
Tseng et al,[13] 2022	Positive test for SARS-CoV-2.
Chung et al,[14] 2022	Reverse transcription polymerase chain reaction (RT-PCR) positive test.
Husin et al,[15] 2022	Reverse transcriptase-PCR (RT-PCR) and antigen rapid (RTK-Ag) positive test.
Prunas et al,[16] 2022	Positive RT-PCR test.
Tartof et al,[17] 2021	PCR positive test from any sample (ie, bronchial lavage, nasopharyngeal or nasal swab, oropharyngeal swab, throat swab, saliva, sputum, or tracheal aspirate) in any clinical setting regardless of the presence of symptoms.
Menni et al,[18] 2022	Lateral flow or PCR test positivity.
Starrfelt et al,[19] 2022	Positive SARS-CoV-2 PCR test.
Šmíd et al,[20] 2022	PCR-confirmed positive test of any type of sample regardless of the presence of symptoms.
Florea et al,[21] 2022	Positive molecular test or a COVID-19 diagnosis code for both symptomatic and asymptomatic infections.
Fabiani et al,[22] 2022	Positive test for SARS-CoV-2 infection in Italy, confirmed in a laboratory by polymerase chain reaction (PCR) or, from 15 January 2021, also by antigen test.
Fabiani et al,[23] 2022	Positive test for SARS-CoV-2 infection in Italy, confirmed in a laboratory by polymerase chain reaction (PCR) or, from 15 January 2021, also by antigen test.
Gram et al,[24] 2022	Positive PCR test for SARS-CoV-3 infection (both asymptomatic and symptomatic).
Vokó et al,[25] 2022	Registered SARS-CoV-2 infection.
Horne et al,[26] 2022	Positive SARS-CoV-2 test. Both polymerase chain reaction and lateral flow tests were included, without differentiation between symptomatic and asymptomatic infection.
Goldberg et al,[27] 2021	Positive SARS-CoV-2 PCR test.
Rennert et al,[28] 2022	Positive saliva polymerase-chain-reaction (PCR) tests.
Veneti et al,[29] 2022	Positive SARS-CoV-2 PCR test (both symptomatic and asymptomatic reported cases).
Hansen et al,[30] 2022	SARS-CoV-2 infections confirmed by reverse transcription PCR.
Sacco et al,[31] 2022	Notified SARS-CoV-2 infection (asymptomatic or symptomatic).
Richterman et al,[46] 2022	PCR positive test for SARS-CoV-2 infection.

eTable 5. Quality Assessment for Case-Control Studies According to Newcastle-Ottawa Scale.

	**Selection**				**Comparability**	**Exposure**				
**Author**	**Is the Case Definition Adequate?**	**Representativeness of the Cases**	**Selection of Controls**	**Definition of Controls**	**Comparability of Cases and Controls on the Basis of the Design or Analysis (max 2 points)**	**Ascertain-ment of exposure**	**Same method of ascertain-ment for cases and controls**	**Non-Response rate**	**Total score**	**Risk of bias**
Chemaitelly et al,[9] 2021	1	1	1	1	2	1	1	0	**8**	**Low**
Skowronski et al,[10] 2022	1	1	1	0	2	1	1	0	**7**	**Low**
Lim et al,[11] 2022	1	1	1	1	2	1	1	0	**8**	**Low**
Bruxvoort et al,[12] 2021	1	1	1	1	2	1	1	0	**8**	**Low**
Tseng et al,[13] 2022	1	1	1	1	2	1	1	0	**8**	**Low**
Chung et al,[14] 2022	1	1	1	1	2	1	1	0	**8**	**Low**
Husin et al,[15] 2022	1	1	1	0	2	1	1	0	**7**	**Low**
Prunas et al,[16] 2022	1	1	1	1	1	1	1	0	**7**	**Low**
Andrews et al,[32] 2022	1	1	1	1	2	1	1	0	**8**	**Low**
Andrews et al,[33] 2022	1	1	1	1	2	1	1	0	**8**	**Low**
Katikireddi et al,[34] 2022	1	1	1	1	2	1	1	0	**8**	**Low**
Ranzani et al,[35] 2022	1	1	1	1	2	1	1	0	**8**	**Low**
Suarez Castillo et al,[36] 2022	1	1	1	1	1	1	1	0	**7**	**Low**

© 2023 Menegale F et al. *JAMA Network Open.*

Buchan et al,[37] 2022	1	1	1	1	2	2	1	0	**7**	**Low**
Kissling et al,[38] 2022	1	0	1	1	2	1	1	0	**7**	**Low**
Florentino et al,[39] 2022	1	1	1	1	2	1	1	0	**8**	**Low**
Powell et al,[40] 2022	1	1	1	1	2	1	1	0	**8**	**Low**
Buchan et al,[41] 2022	1	1	1	1	2	1	1	0	**8**	**Low**
Chemaitelly et al,[42] 2022	1	1	1	1	2	1	1	0	**8**	**Low**
Malhotra et al,[43] 2022	1	1	1	1	2	1	1	0	**8**	**Low**
Arashiro et al,[44] 2022	1	1	1	1	2	1	1	0	**8**	**Low**
Richterman et al,[46] 2022	1	1	1	1	2	1	1	0	**8**	**Low**
Cerqueira-Silva et al,[47] 2022	1	1	1	1	2	1	1	0	**8**	**Low**
Andrews et al,[48] 2022	1	1	1	1	2	1	1	0	**8**	**Low**

eTable 6. Quality Assessment for Cohort Studies According to Newcastle-Ottawa Scale.

	**Selection**				**Comparability**	**Outcome**				
**Author**	**Representative-ness of the Exposed Cohort**	**Selection of the non exposed cohort**	**Ascertain-ment of exposure**	**Demonstra-tion that outcome of interest was not present at start of study**	**Comparability of cohorts on the basis of the design or analysis**	**Assess-ment of outcome**	**Was follow-up long enough for outcomes to occur**	**Adequacy of follow up of cohorts**	**Total score**	**Risk of bias**
Tartof et al,[17] 2021	1	1	1	1	2	1	1	0	**8**	**Low**
Menni et al,[18] 2022	0	1	0	1	2	0	1	1	**6**	**Moderate**
Starrfelt et al,[19] 2022	1	1	1	1	2	1	1	0	**8**	**Low**
Šmíd et al,[20] 2022	1	1	1	0	1	1	1	0	**7**	**Low**
Florea et al,[21] 2022	1	1	1	0	2	1	1	0	**7**	**Low**
Fabiani et al,[22] 2022	1	1	1	1	2	1	1	0	**8**	**Low**
Fabiani et al,[23] 2022	1	1	1	1	2	1	1	0	**8**	**Low**
Gram et al,[24] 2022	1	1	1	1	2	1	1	0	**8**	**Low**
Vokó et al,[25] 2022	1	1	1	1	2	1	1	0	**8**	**Low**
Horne et al,[26] 2022	1	1	1	1	2	1	1	0	**8**	**Low**
Goldberg et al,[27] 2021	1	1	1	1	2	1	1	0	**8**	**Low**
Rennert et al,[28] 2022	0	1	1	1	2	1	1	0	**7**	**Low**
Veneti et al,[29] 2022	1	1	1	1	2	1	1	0	**8**	**Low**
Hansen et al,[30] 2022	1	1	1	1	2	1	1	0	**8**	**Low**
Sacco et al,[31] 2022	1	1	1	1	2	1	1	0	**8**	**Low**

© 2023 Menegale F et al. *JAMA Network Open.*

Rudan et al,[45] 2022	1	1	1	0	2	1	1	0	**7**	**Low**

eTable 7. Model Estimates of VE After the Ramp-Up (*A*), of the VE Waning Rate (*W*), and of the Half-Life of VE Against Symptomatic Disease With Delta and Omicron After Primary Vaccination Cycle and Booster Dose.

	**VE against symptomatic disease with Delta**			**VE against symptomatic disease with Omicron**			**Reference**
	**VE at 14 days from last dose administration (%) [95%CI]**	**Waning rate (month^{-1}) [95%CI]**	**Half-life (days)[a] [95%CI]**	**VE at 14 days from last dose administration (%) [95%CI]**	**Waning rate (month^{-1}) [95%CI]**	**Half-life (days)[a] [95%CI]**	
**BNT162b2**							
**2 doses**							
	96.2 [92.5 - 99.2]	0.0761 [0.0602 - 0.0903]	287.2 [244.3 - 359.1]	NA	NA	NA	Andrews et al,[32] 2022
	93.0 [91.4 - 94.6]	0.0659 [0.0590 - 0.0723]	329.6 [301.4 - 366.2]	76.3 [70.6 - 82.4]	0.4025 [0.3507 - 0.4636]	65.7 [58.9 - 73.3]	Andrews et al,[33] 2022
	83.9 [65.4 - 94.3]	0.0938 [0.0024 - 0.1821]	235.7 [128.2 - 8595.9]	NA	NA	NA	Kissling et al,[38] 2022
	NA	NA	NA	67.5 [59.3 - 75.3]	0.3367 [0.2639 - 0.4146]	75.8 [64.2 - 92.8]	Chemaitelly et al,[42] 2022
**Booster**							
**2 doses of BNT162b2 + BNT162b2**	NA	NA	NA	72.4 [66.7 - 80.3]	0.2238 [0.1328 - 0.3533]	106.9 [72.9 - 170.6]	Andrews et al,[33] 2022
	NA	NA	NA	58.2 [49.8 - 69.4]	0.1833 [0.0510 - 0.3251]	127.4 [78.0 - 422.1]	Chemaitelly et al,[42] 2022
**2 doses of BNT162b2 + mRNA-1273**	NA	NA	NA	76.5 [72.5 - 81.5]	0.1536 [0.0847 - 0.2477]	149.3 [98.0 - 259.6]	Andrews et al,[33] 2022
**mRNA-1273**							
**2 doses**							
	96.2 [93.1 - 99.0]	0.0471 [0.0356 - 0.0575]	455.6 [375.7 - 598.9]	83.6 [80.4 - 86.8]	0.3518 [0.3291 - 0.3775]	73.1 [69.1 - 77.2]	Andrews et al,[33] 2022
	NA	NA	NA	62.9 [53.9 - 71.2]	0.2856 [0.2198 - 0.3798]	86.8 [68.8 - 108.6]	Chemaitelly et al,[42] 2022
**Booster**							
**2 doses of mRNA-1273 + mRNA-1723**	NA	NA	NA	56.3 [52.1 - 70.3]	0.0034 [0.0000 - 0.0191]	219.5 [50.2 - 15512.8]	Chemaitelly et al,[42] 2022
**ChAdOx1 nCoV-19**							
**2 doses**							
	74.6 [71.3 - 78.2]	0.1075 [0.0872 - 0.1278	207.4 [176.7 - 252.4]	NA	NA	NA	Andrews et al,[32] 2022
	88.5 [82.5 - 94.7]	0.1267 [0.0977 - 0.1520]	178.2 [150.8 - 226.7]	56.1 [44.4 - 71.1]	0.3740 [0.2588 - 0.5802]	69.6 [49.8 - 94.4]	Andrews et al,[33] 2022
	73.1 [69.7 - 76.6]	0.1196 [0.0987 - 0.1401]	187.9 [162.5 - 224.7]	NA	NA	NA	Katikireddi et al,[34] 2022
	72.4 [68.3 - 76.5]	0.0586 [0.0175 - 0.1020]	369.1 [217.9 - 1204.4]	NA	NA	NA	Kissling et al,[38] 2022
**Booster**							
**2 doses of ChAdOx1 nCoV-19 + BNT162b2**	NA	NA	NA	66.3 [61.4 – 72.0]	0.1822 [0.0916 - 0.2728]	128.1 [90.2 – 241.0]	Andrews et al,[33] 2022
**2 doses of ChAdOx1 nCoV-19 + mRNA-1273**	NA	NA	NA	73.8 [68.8 - 79]	0.1540 [0.0702 - 0.2335]	149.0 [103.1 - 310.4]	Andrews et al,[33] 2022

© 2023 Menegale F et al. *JAMA Network Open.*

	VE against symptomatic disease with Delta			VE against symptomatic disease with Omicron			Reference
2 doses of ChAdOx1 nCoV-19 + ChAdOx1 nCoV-19	NA	NA	NA	59.6 [54.0 - 66.7]	0.1951 [0.1011 - 0.3329]	120.6 [76.5 - 219.8]	Andrews et al,[33] 2022
**CoronaVac**							
**2 doses**							
	57.1 [52.6 - 63.8]	0.1161 [0.0735 - 0.1776]	193.0 [131.1 - 296.7]	53.0 [38.3 - 90.7]	0.8958 [0.5038 - 2.1720]	37.2 [23.6 - 55.3]	Ranzani et al,[35] 2022
**Booster**							
**2 doses of CoronaVac + BNT162b2**	NA	NA	NA	71.3 [68.3 - 74.3]	0.3939 [0.3569 - 0.4358]	66.8 [61.7 - 72.3]	Cerqueira-Silva et al,[47] 2022
**Ad26.COV2.S**							
**1 dose**							
	54.9 [48.5 - 77.8]	0.0566 [0.0001 - 0.3003]	381.1 [83.2 - 19832.4]	NA	NA	NA	Kissling et al,[38] 2022
**Unspecified/Mixed products**							
**Primary cycle**							
	96.1 [93.3 - 98.6]	0.0174 [0.0106 - 0.0241]	1211.2 [877.4 - 1975.3]				Chung et al,[14] 2022[b]
	96.1 [93.9 - 98.5]	0.0150 [0.0065 - 0.0219]	1399.5 [964.4 - 3210.9]				Chung et al,[14] 2022[c]
	80.8 [78.6 - 83.0]	0.0818 [0.0722 - 0.0929]	268.1 [237.9 - 302.2]				Suarez Castillo et al,[36] 2022
	90.2 [88.1 - 92.5]	0.0078 [0.0016 - 0.0130]	2674.4 [1618.6 - 13030.4]	54.3 [28.3 - 94.8]	0.7918 [0.2272 - 3.7306]	40.3 [19.6 - 105.5]	Buchan et al,[37] 2022
	NA	NA	NA	59.5 [48.7 - 75.6]	0.1813 [0.0992 - 0.3156]	128.7 [79.9 - 223.6]	Malhotra et al,[43] 2022
	NA	NA	NA	56.8 [53.7 - 64.8]	0.0158 [0.0006 - 0.0632]	1328.9 [342.8 - 36693.4]	Arashiro et al,[44] 2022

[a] log(2)/w + 14 days
[b] Any 2-dose mRNA schedule, including BNT162b2/BNT162b2, mRNA-1273/mRNA-1273, and BNT162b2/mRNA-1273
[c] Any ChAdOx1 nCov-19-containing schedule, including ChAdOx1 nCov-19/ChAdOx1 nCov-19, ChAdOx1 nCov-19/BNT162b2, and ChAdOx1 nCov-19/mRNA-1273

eTable 8. Model Estimates of VE After the Ramp-Up (*A*), of the VE Waning Rate (*W*), and of the Half-Life of VE Against Laboratory-Confirmed SARS-Cov-2 Infection With Delta and Omicron After Primary Vaccination Cycle and Booster Dose.

	VE against laboratory-confirmed infection with Delta			VE against laboratory-confirmed infection with Omicron			Reference
	**VE at 14 days from last dose administration (%) [95%CI]**	**Waning rate (month^{-1}) [95%CI]**	**Half-life (days)[a] [95%CI]**	**VE at 14 days from last dose administration (%) [95%CI]**	**Waning rate (month^{-1}) [95%CI]**	**Half-life (days)[a] [95%CI]**	
**BNT162b2**							
**2 doses**							
	94.2 [80.7 - 99.7]	0.2990 [0.2140 - 0.3778]	83.5 [69.0 - 111.2]	NA	NA	NA	Chemaitelly et al,[9] 2021
	93.7 [91.8 - 95.4]	0.0233 [0.0187 - 0.0266]	905.0 [795.7 - 1128.9]	NA	NA	NA	Skowronski et al,[10] 2022[b]
	92.3 [90.5 - 94.2]	0.0232 [0.0181 - 0.0282]	909.1 [751.5 - 1163.9]	NA	NA	NA	Skowronski et al,[10] 2022[c]
	77.9 [60.0 - 96.8]	0.1208 [0.0117 - 0.2697]	186.1 [91.1 - 1794.5]	NA	NA	NA	Lim et al,[11] 2022
	94.3 [79.9 - 99.8]	0.1120 [0.0269 - 0.1599]	199.7 [144.0 - 786.0]	NA	NA	NA	Tartof et al,[17] 2021

© 2023 Menegale F et al. *JAMA Network Open.*

	VE against laboratory-confirmed infection with Delta			VE against laboratory-confirmed infection with Omicron			Reference
	91.7 [90.6 - 93.0]	0.0260 [0.0232 - 0.0294]	814.9 [721.6 - 910.1]	NA	NA	NA	Menni et al,[18] 2022
	89.0 [86.1 - 92.1]	0.1480 [0.1378 - 0.1593]	154.5 [144.5 – 165.0]	NA	NA	NA	Starrfelt et al,[19] 2022
	88.3 [83.0 - 93.8]	0.0784 [0.0502 - 0.1086]	279.4 [205.4 - 427.8]	65.1 [58.1 - 73.5]	0.2969 [0.2275 - 0.3896]	84.1 [67.4 - 105.4]	Šmíd et al,[20] 2022
	76.5 [65.2 - 87.6]	0.0712 [0.0370 - 0.1048]	306.0 [212.4 - 576.3]	NA	NA	NA	Vokó et al,[25] 2022
	90.5 [63.8 - 99.3]	0.3177 [0.1482 - 0.5808]	79.4 [49.8 - 154.3]	NA	NA	NA	Horne et al,[26] 2022
	97.4 [91.2 - 99.9]	0.0950 [0.0733 - 0.1124]	232.9 [199.0 - 297.7]	NA	NA	NA	Goldberg et al,[27] 2021
	NA	NA	NA	35.9 [29.1 - 44.8]	0.1299 [0.0236 - 0.2764]	174.1 [89.2 - 894.3]	Hansen et al,[30] 2022
**Booster**							
**2 doses of BNT162b2 + BNT162b2**	NA	NA	NA	47.1 [43.0 - 51.5]	0.0727 [0.0267 - 0.1313]	300.0 [172.4 - 793.5]	Hansen et al,[30] 2022
	NA	NA	NA	82.4 [71.5 - 91.6]	0.1754 [0.1002 - 0.2444]	132.5 [99.1 - 221.5]	Richterman et al,[46] 2022
**mRNA-1273**							
**2 doses**							
	97.9 [93.5 - 99.9]	0.0492 [0.0383 - 0.0597]	436.6 [362.6 - 556.9]	NA	NA	NA	Skowronski et al,[10] 2022[b]
	96.2 [88.6 - 99.9]	0.0518 [0.0258 - 0.0741]	415.4 [294.4 - 819.1]	NA	NA	NA	Skowronski et al,[10] 2022[c]
	96.3 [91.6 - 99.8]	0.0427 [0.0281 - 0.0549]	500.8 [393.0 - 754.1]	NA	NA	NA	Bruxvoort et al,[12] 2021
	83.7 [78.3 - 90.2]	0.0417 [0.0238 - 0.0588]	512.5 [367.6 - 888.1]	56.5 [51.3 - 62.0]	0.2129 [0.1803 - 0.2536]	111.7 [96.0 - 129.3]	Tseng et al,[13] 2022
	94.8 [93.5 - 96.0]	0.0276 [0.0220 - 0.0335]	767.8 [635.3 - 959.6]	NA	NA	NA	Menni et al,[18] 2022
	97.0 [91.4 - 99.7]	0.1184 [0.1034 - 0.1293]	189.7 [174.8 - 215.1]	NA	NA	NA	Starrfelt et al,[19] 2022
	75.9 [65.8 - 93.7]	0.0302 [0.0003 - 0.1303]	703.4 [173.6 - 75627.7]	52.3 [47.5 - 58.3]	0.0825 [0.0386 - 0.1336]	266.1 [169.7 - 553.3]	Šmíd et al,[20] 2022
	90.0 [86.5 - 93.7]	0.0288 [0.0192 - 0.0376]	735.3 [566.6 - 1095.8]	NA	NA	NA	Florea et al,[21] 2022
	89.3 [78.3 - 97.9]	0.0804 [0.0323 - 0.1091]	272.5 [204.6 - 657.4]	NA	NA	NA	Vokó et al,[25] 2022
	NA	NA	NA	37.5 [30.7 - 45.1]	0.1832 [0.0665 - 0.3152]	127.5 [80.0 - 326.8]	Hansen et al,[30] 2022
**Booster**							
**2 doses of mRNA-1273 + mRNA-1723**	NA	NA	NA	49.5 [46.4 - 52.8]	0.0849 [0.0450 - 0.1254]	258.9 [179.8 - 476.6]	Hansen et al,[30] 2022
**ChAdOx1 nCoV-19**							
**2 doses**							
	78.0 [74.1 - 82.3]	0.0206 [0.0071 - 0.0364]	1022.8 [585.4 – 2945.0]	NA	NA	NA	Skowronski et al,[10] 2022[b]
	96.4 [88.1 - 99.9]	0.0686 [0.0425 - 0.0850]	317.0 [258.6 - 502.8]	NA	NA	NA	Skowronski et al,[10] 2022[c]
	81.6 [79.0 - 84.0]	0.0194 [0.0086 - 0.0293]	1085.8 [723.8 - 2443.4]	NA	NA	NA	Menni et al,[18] 2022

© 2023 Menegale F et al. *JAMA Network Open.*

	VE against laboratory-confirmed infection with Delta			VE against laboratory-confirmed infection with Omicron			Reference
	54.1 [44.2 - 77.8]	0.1495 [0.0897 - 0.2309]	153.1 [104.0 - 245.9]	NA	NA	NA	Vokó et al,[25] 2022
	63.9 [37.7 - 99.3]	0.9968 [0.2973 - 2.1279]	34.9 [23.8 - 83.9]	NA	NA	NA	Horne et al,[26] 2022
**Ad26.COV2.S**							
**1 dose**							
	61.5 [58.4 - 68.1]	0.0253 [0.0037 - 0.0643]	836.6 [337.4 - 5649.3]	53.2 [48.2 - 60.7]	0.1226 [0.0739 - 0.1985]	183.6 [118.8 - 295.4]	Šmíd et al,[20] 2022
	48.0 [36.2 - 77.0]	0.0698 [0.0020 - 0.1986]	311.8 [118.7 - 10436.1]	NA	NA	NA	Vokó et al,[25] 2022
**BBIBP-CorV**							
**2 doses**							
	39.7 [10.6 - 97.0]	2.1048 [0.0869 - 12.1975]	23.9 [15.7 - 253.2]	NA	NA	NA	Vokó et al,[25] 2022
**Gam-COVID-Vac**							
**2 doses**							
	59.5 [41.2 - 86.2]	0.2232 [0.0899 - 0.3627]	107.2 [71.3 - 245.4]	NA	NA	NA	Vokó et al,[25] 2022
**Unspecified/Mixed products**							
**Primary cycle**							
	93.1 [90.3 - 95.7]	0.0279 [0.0209 - 0.0347]	759.6 [612.4 - 1007.4]	NA	NA	NA	Chung et al,[14] 2022[d]
	93.1 [89.2 - 97.2]	0.0435 [0.0279 - 0.0583]	491.9 [370.9 - 758.2]	NA	NA	NA	Chung et al,[14] 2022[e]
	94.1 [88.5 - 98.6]	0.1537 [0.1349 - 0.1704]	149.3 [136 - 168.1]	NA	NA	NA	Starrfelt et al,[19] 2022[f]
	92.7 [71.8 - 99.8]	0.1526 [0.0707 - 0.2130]	150.2 [111.6 - 308.2]	NA	NA	NA	Starrfelt et al,[19] 2022[g]
	91.6 [84.9 - 97.7]	0.1496 [0.1250 - 0.1728]	153.0 [134.4 - 180.4]	NA	NA	NA	Fabiani et al,[22] 2022
	87.8 [61.1 - 99.4]	0.1987 [0.1090 - 0.2642]	118.7 [92.7 - 204.7]	NA	NA	NA	Fabiani et al,[23] 2022
	92.9 [79.0 - 97.7]	0.0795 [0.0269 - 0.1035]	275.6 [215.0 - 785.8]	39.1 [33.9 - 46.6]	0.0956 [0.0162 - 0.2207]	231.4 [108.2 - 1300.5]	Gram et al,[24] 2022
**Booster**							
**3 doses of any mRNA vaccine**	NA	NA	NA	55.5 [52.3 - 59.5]	0.0204 [0.0012 - 0.0542]	1032.3 [397.7 - 17712.2]	Gram et al,[24] 2022

[a] log(2)/w + 14 days
[b] Data from British Columbia
[c] Data from Quebec
[d] Any 2-dose mRNA schedule, including BNT162b2/BNT162b2, mRNA-1273/mRNA-1273, and BNT162b2/mRNA-1273
[e] Any ChAdOx1 nCov-19-containing schedule, including ChAdOx1 nCov-19/ChAdOx1 nCov-19, ChAdOx1 nCov-19/BNT162b2, and ChAdOx1 nCov-19/mRNA-1273
[f] Any mRNA vaccine product
[g] Any vaccine among BNT162b2, mRNA-1273 and ChAdOx1 nCov-19

© 2023 Menegale F et al. *JAMA Network Open.*

eAppendix 1. Data Extraction and Selection.
Data has been extracted independently by two reviewers. Percentage estimates from the original studies were reported in a spreadsheet. Results were compared and potential discrepancies re-assessed and resolved. Estimates of VE against Delta or Omicron infection and/or symptomatic disease for any vaccine product or combination of products, at different times from the administration of the last dose, were extracted from the original studies retrieved from the search to inform a simple statistical model to estimate the progressive waning of immunity. We considered both VE estimates associated with primary vaccination cycle (1 dose for Ad26.COV2.S and 2 doses for the other vaccine products) and primary vaccination cycle followed by a booster dose. Descriptions of the considered endpoints presented in the eligible articles are summarized in eTables 5-6. Data were complete, with no missing information for COVID-19 vaccine products, considered endpoints, or population characteristics. To minimize potential biases led by the initial ramp-up of vaccine-induced protection, we excluded data points associated with VE measured during the first 14 days following the administration of the considered dose. Data points associated with less than 20 infections observed in the vaccinated group were excluded from the analysis.

eAppendix 2. Model Details.
VE is modeled as an exponential decay function of time described as:

$$\mathrm{VE}(t) = Ae^{-w \cdot t} \quad (1)$$

where:
- *t* is the number of days from maximum protection, which is assumed to occur after 14 days from the administration of any dose;
- *A* is the VE after 14 days from the administration of the last dose;
- *w* is the waning rate associated with the vaccine-induced protection against the considered endpoint.

Free model parameters *A* and *w* were estimated for each study via a Markov chain Monte Carlo (MCMC) approach with Metropolis-within-Gibbs sampling algorithm applied to the normal likelihood of observing the average values of VE estimated in the original study at different time intervals from vaccination.

The model was informed with mean VE estimates at different time intervals retrieved from the articles included after the systematic review. Such estimates were associated with a specific time interval (expressed in days) and were interpreted as the mean VE(t) in that time interval: for example, if we extracted from one study a mean VE estimate of 70% evaluated between 30 and 60 days after the administration of last dose, we considered it as the mean VE(t) in that interval of time. According to the proposed exponential decay function, the corresponding mean modeled VE in a specific interval of time $[t_1, t_2]$ (expressed in days) can be computed as follows:

$$\overline{VE}\,(t_1, t_2) = \frac{\sum_{t=t_1}^{t_2} VE(t)}{t_2 - t_1 + 1} = \frac{\sum_{t=t_1}^{t_2} Ae^{-w \cdot t}}{t_2 - t_1 + 1} \quad (2)$$

where the time step of the sum is 1 day. Let us consider a VE estimate for a specific interval of time $[t_1^i, t_2^i]$ extracted from a selected article (with *i* = 1… *n*, where *n* is the number of VE estimates extracted from that article) and denote it with $VE_{obs}(t_1^i, t_2^i)$. We assumed that $VE_{obs}(t_1^i, t_2^i)$ is distributed according to a normal distribution, with mean equal to the modeled estimate for the same time interval computed according to (2) and variance σ^2, i.e.

$$VE_{obs}(t_1^i, t_2^i) \sim \mathcal{N}(\overline{VE}(t_1^i, t_2^i), \sigma^2) \quad (3)$$

© 2023 Menegale F et al. *JAMA Network Open.*

We applied the Gibbs sampling to likelihood (3), using Metropolis-Hastings random walk update for parameters A and w and Gibbs update for σ^2.
Regarding the prior distributions, we chose $A \sim U(0{,}100\%)$ and $w \sim U(0{,}1\ days^{-1})$ (where $U(a,b)$ denotes the uniform distribution between a and b). As for the variance, we decided to use the precision $\tau = 1/\sigma^2$ instead and assumed $\tau \sim Gamma(1,1)$. This choice was made to exploit the fact that the fully conditioned posterior distribution of is again a Gamma distribution from which is possible to sample to perform the Gibbs update.

Our main analysis focused on providing estimates of VE at the population level. The main analysis relied on studies reporting VE estimates for a sufficiently wide age range (i.e., covering ages for at least 30 years, and including individuals aged 25-60 years). For studies reporting estimates for specific age groups only, a separate fit for each age group was performed and the VE at population level was estimated by combining the posterior distributions associated with each age group in a mixture distribution weighted by the proportion of individuals in each age group included in the original study.

eAppendix 3. Characteristics of the Included Studies.

Reference groups

In 38 studies[9–21,24–48], estimates were obtained by using unvaccinated individuals as a reference group. Individuals who have received a single dose not earlier than 14 days were assumed as proxy for unvaccinated individuals in Fabiani et al, 2022[22]. Similarly, the reference group was defined by subjects who have received a single dose from at least 4 days and not more than 10 days in Fabiani et al, 2022[23]. According to Fabiani et al[22,23], the rationale for this assumption was that unvaccinated individuals might undergo a higher number of tests and have their social habits altered by restrictions (e.g., EU Digital COVID certificate), thus leading to biased VE estimates when considering them as a reference group.

Vaccine products

The articles included in this analysis provide estimates of VE for BNT162b2 (Pfizer BioNTech COVID-19 vaccine) (n=26), mRNA-1273 (Moderna COVID-19 vaccine) (n=12), ChAdOx1 nCoV-19 (Oxford-AstraZeneca COVID-19 vaccine) (n=8), Ad26.COV2.S (Janssen COVID-19 vaccine) (n=3), CoronaVac (Sinovac COVID-19 vaccine) (n=3), BBIBP-CorV (Sinopharm COVID-19 vaccine) (n=1), Gam-COVID-Vac (Sputnik V COVID-19 vaccine) (n=1) either against Delta (n=32) or Omicron (n=17) variants[9–48]. Eight studies provide estimates of VE over time for unspecified vaccine products[14,22–24,36,37,43,44], although mRNA vaccines were mainly adopted in the analyzed populations in 7 out of these 8 studies.

Endpoints and variants

None of the analyzed studies found a clear temporal waning of VE of a booster dose against Delta[24,33,48], possibly due to the short follow-up associated with the available records, given that Omicron suppressed Delta circulation soon after the start of boosting campaigns. From the selected papers, we extracted: 1) twenty-one[9–29] studies providing VE estimates for primary vaccination against any SARS-CoV-2 laboratory-confirmed infection (asymptomatic or symptomatic) for Delta and six[13,20,24,29–31] for Omicron; 2) twelve[14,16,32–41] studies providing VE estimates for primary vaccination cycle against symptomatic disease for Delta and nine[33,35,37,39–41,43–45] Omicron; 3) three[24,30,46] studies providing VE estimates for primary vaccination cycle followed by a booster dose against any Omicron SARS-CoV-2 laboratory-confirmed infection (asymptomatic or symptomatic); 4) three[33,42,47] studies providing VE estimates for primary vaccination cycle followed by a booster dose against Omicron symptomatic disease.

Age structure

© 2023 Menegale F et al. *JAMA Network Open.*

Twenty-four studies provided VE estimates at population level[9–14,17–23,30,32–35,37,42–44,46,47]; fifteen studies provided VE estimates by age group[15,16,24–29,31,36,38–41,45]; eight studies provided VE both at population level and for specific age groups[12,18,19,21–23,32,47].

© 2023 Menegale F et al. *JAMA Network Open.*

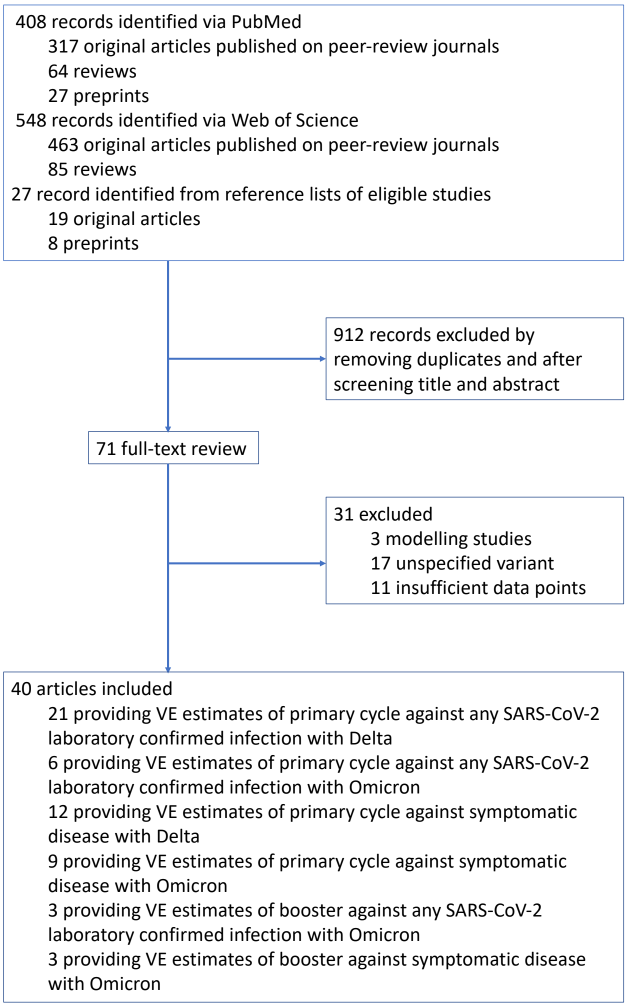

eFigure 1. Study Selection. Flowchart of the Selection of Studies Considered for the Performed Analysis.

© 2023 Menegale F et al. *JAMA Network Open.*

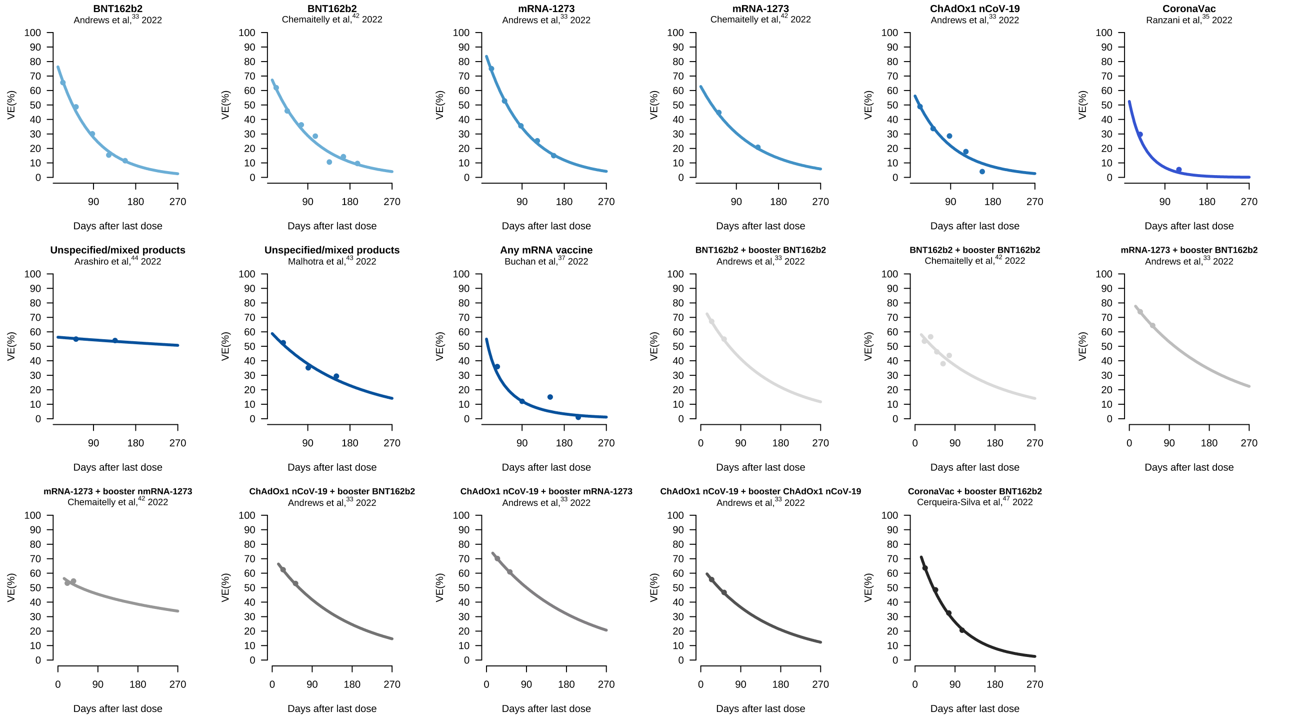

eFigure 2. Effectiveness Over Time of Primary Vaccination Cycle and Booster Vaccination Against Omicron Symptomatic Disease. Estimated vaccine effectiveness (VE) over time against symptomatic disease with Omicron across different vaccine products. Lines: mean estimates; shaded areas: 95% CIs; points: original VE estimates from published articles[33,35,37,42-45,47] (placed at the midpoint of the time interval for which the estimate was obtained).

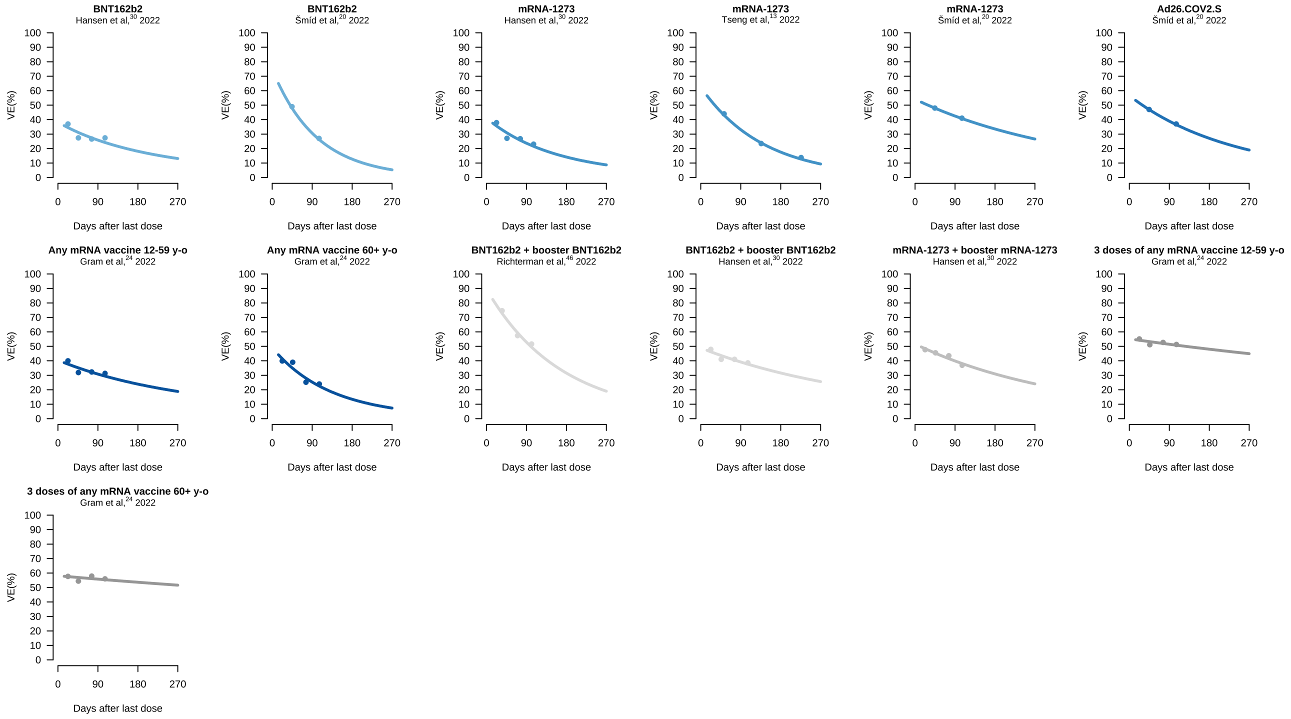

eFigure 3. Effectiveness Over Time of Primary Vaccination Cycle and Booster Vaccination Against Any Omicron Laboratory-Confirmed Infection. Estimated vaccine effectiveness (VE) over time against any laboratory-confirmed SARS-CoV-2 infection with Omicron across different vaccine products. Lines: mean estimates; shaded areas: 95% CIs; points: original VE estimates from published articles[13,20,24,30,46] (placed at the midpoint of the time interval for which the estimate was obtained).

© 2023 Menegale F et al. *JAMA Network Open.*

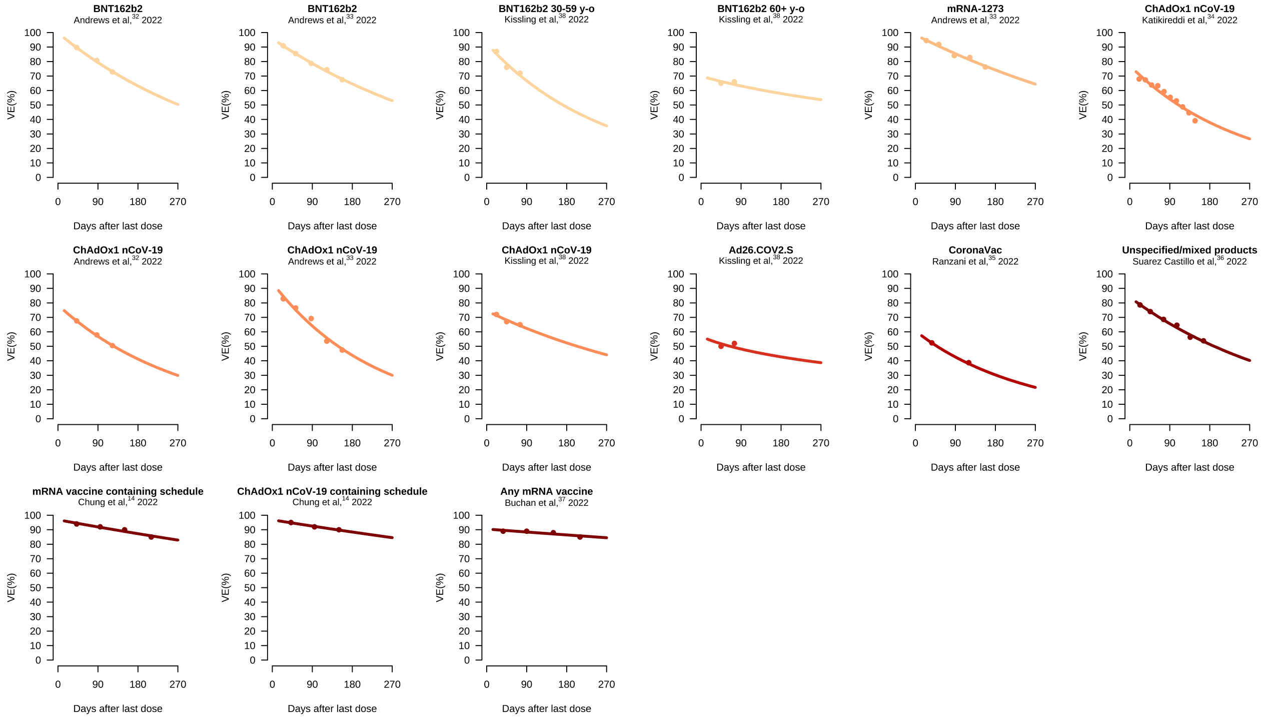

eFigure 4. Effectiveness Over Time of Primary Vaccination Cycle Against Delta Symptomatic Disease. Estimated vaccine effectiveness (VE) over time against symptomatic disease with Delta across different vaccine products. Lines: mean estimates; shaded areas: 95% CIs; points: original VE estimates from published articles[14,32-38] (placed at the midpoint of the time interval for which the estimate was obtained).

© 2023 Menegale F et al. *JAMA Network Open.*

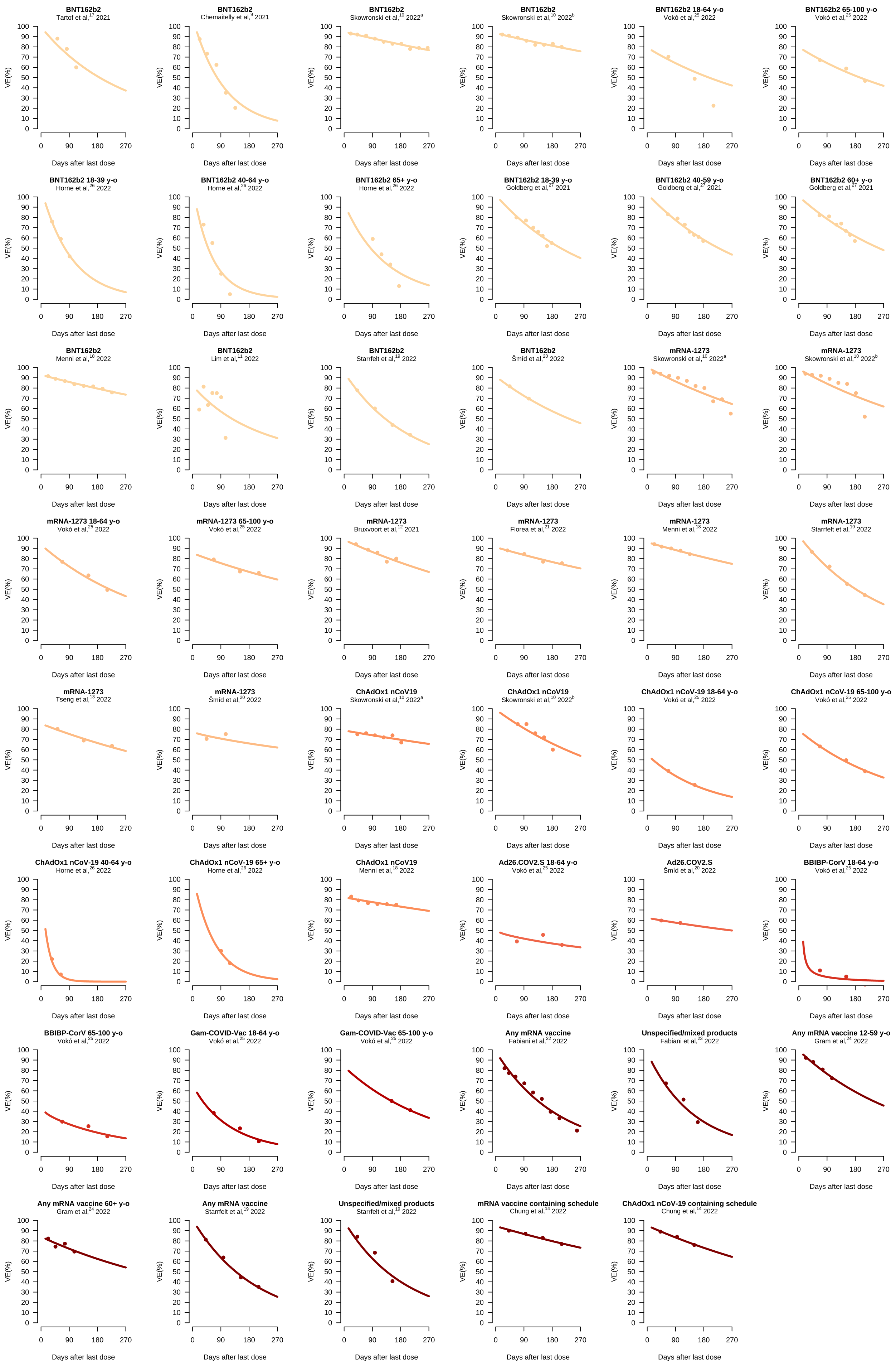

© 2023 Menegale F et al. *JAMA Network Open.*

eFigure 5. Effectiveness Over Time of Primary Vaccination Cycle Against Any Delta Laboratory-Confirmed Infection. Estimated vaccine effectiveness (VE) over time against any laboratory-confirmed SARS-CoV-2 infection with Delta across different vaccine products. Lines: mean estimates; shaded areas: 95% CIs; points: original VE estimates from published articles[9-14,17-27] (placed at the midpoint of the time interval for which the estimate was obtained). [a] Data from British Columbia; [b] Data from Quebec.

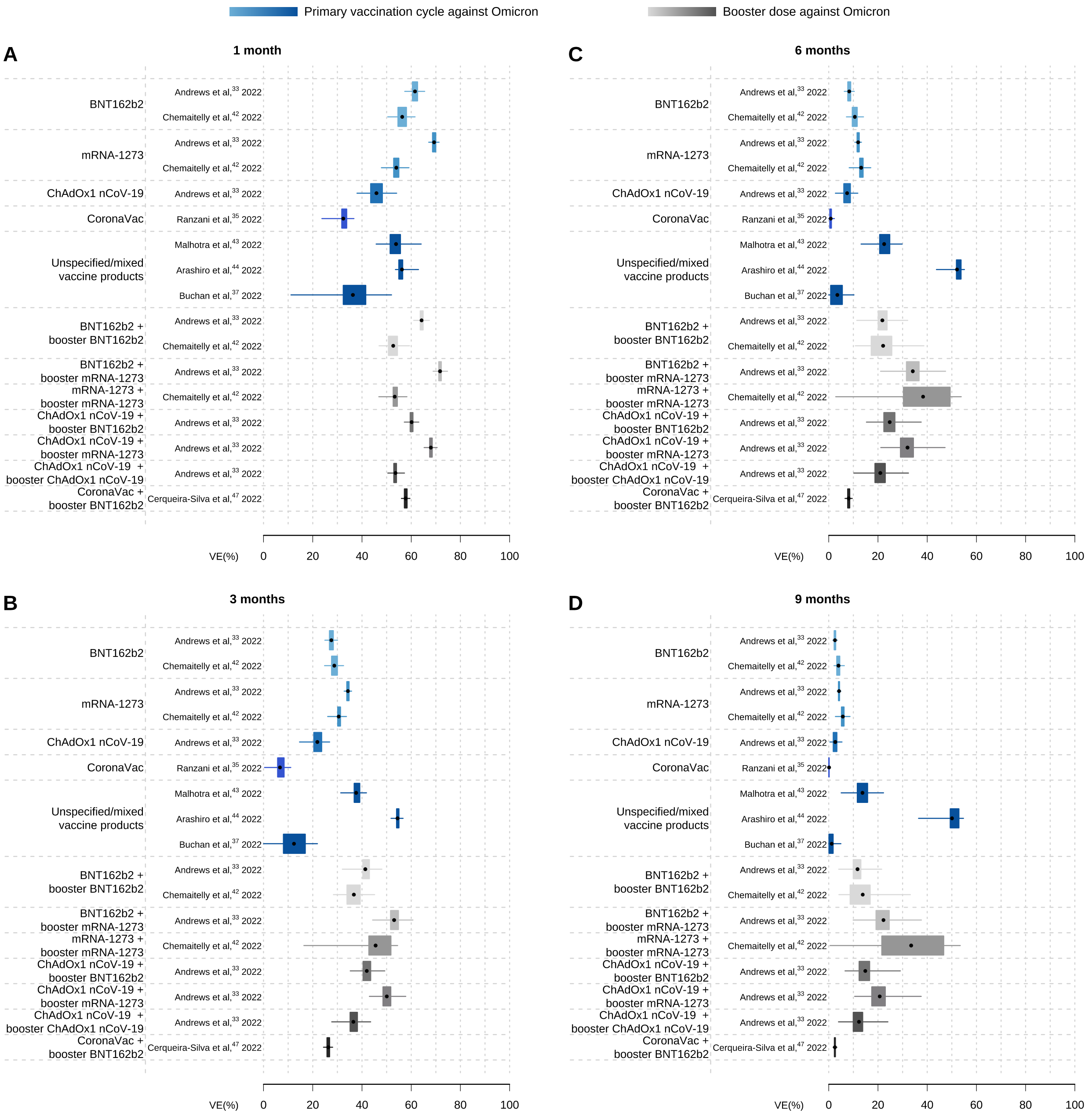

eFigure 6. Effectiveness Over Time of Primary Vaccination Cycle and Booster Vaccination Against Omicron Symptomatic Disease for Single Time Series. Comparison of VE against symptomatic disease with Omicron across different vaccine products at 1 (**A**), 3 (**B**), 6 (**C**), and 9 (**D**) months from the administration of primary vaccination cycle for single time series. Points: mean estimates; boxes: interquartile ranges; whiskers: 95% CIs.

© 2023 Menegale F et al. *JAMA Network Open.*

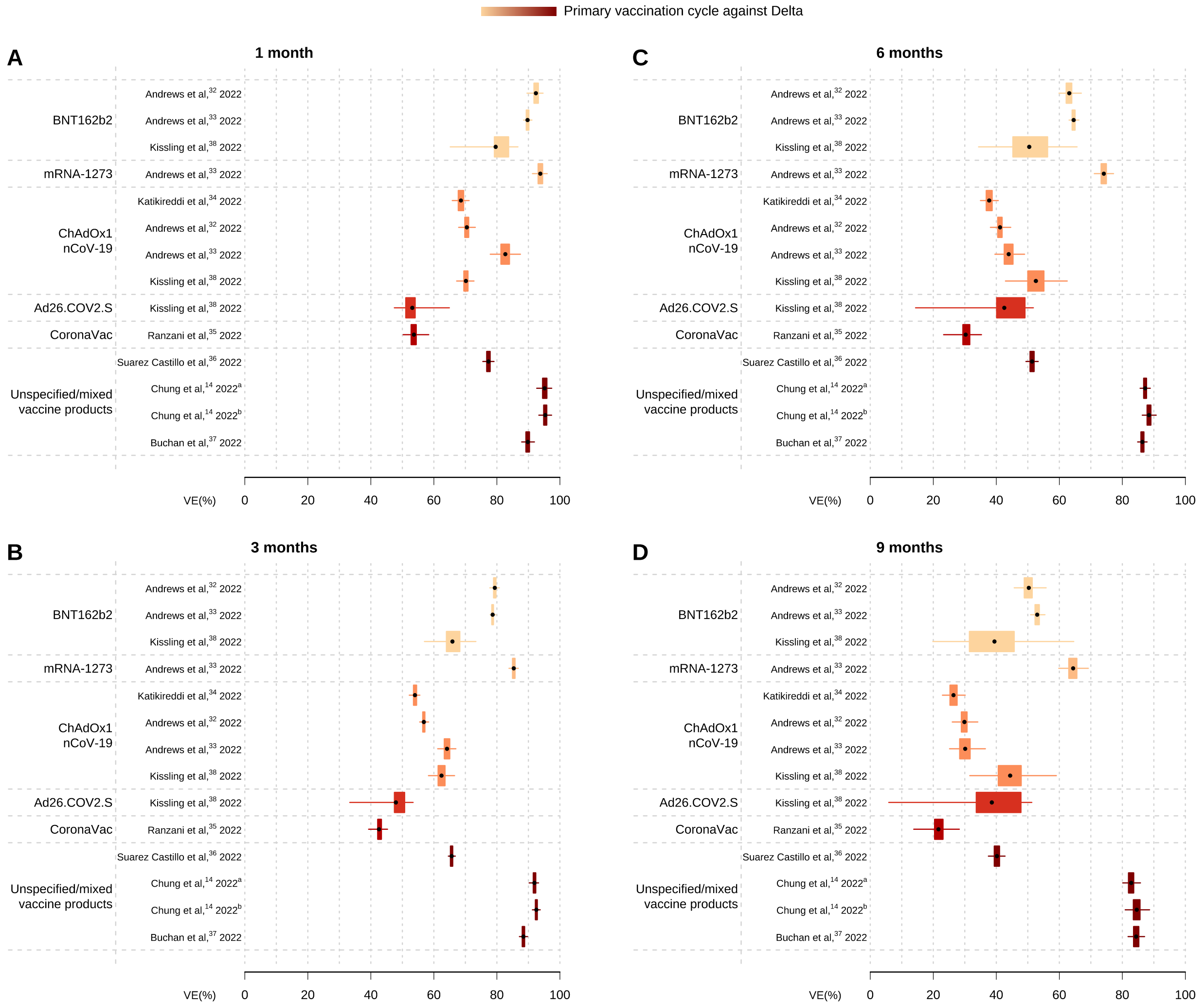

eFigure 7. Effectiveness Over Time of Primary Vaccination Cycle Against Delta Symptomatic Disease for Single Time Series. Comparison of VE against symptomatic disease with Delta across different vaccine products at 1 (**A**), 3 (**B**), 6 (**C**), and 9 (**D**) months from the administration of primary vaccination cycle for single time series. Points: mean estimates; boxes: interquartile ranges; whiskers: 95% CIs. [a]Any 2-dose mRNA schedule, including BNT162b2/BNT162b2, mRNA-1273/mRNA-1273, and BNT162b2/mRNA-1273; [b]Any ChAdOx1 nCov-19-containing schedule, including ChAdOx1 nCov-19/ChAdOx1 nCov-19, ChAdOx1 nCov-19/BNT162b2, and ChAdOx1 nCov-19/mRNA-1273.

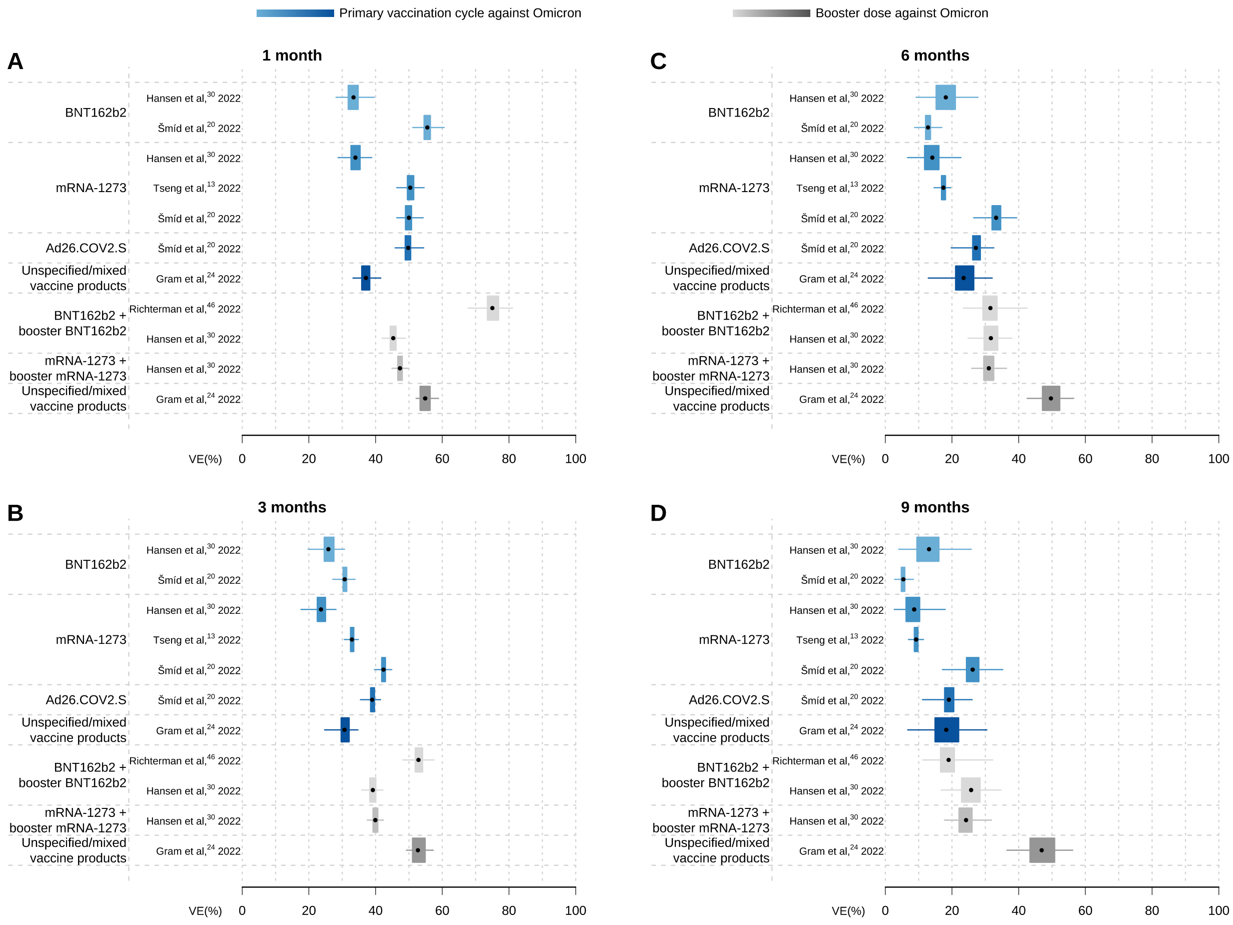

eFigure 8. Effectiveness Over Time of Primary Vaccination Cycle and Booster Vaccination Against Any Omicron Laboratory-Confirmed Infection for Single Time Series. Comparison of VE against any laboratory-confirmed SARS-CoV-2 infection with Omicron across different vaccine products at 1 (**A**), 3 (**B**), 6 (**C**), and 9 (**D**) months from the administration of primary vaccination cycle for single time series. Points: mean estimates; boxes: interquartile ranges; whiskers: 95% CIs.

© 2023 Menegale F et al. *JAMA Network Open.*

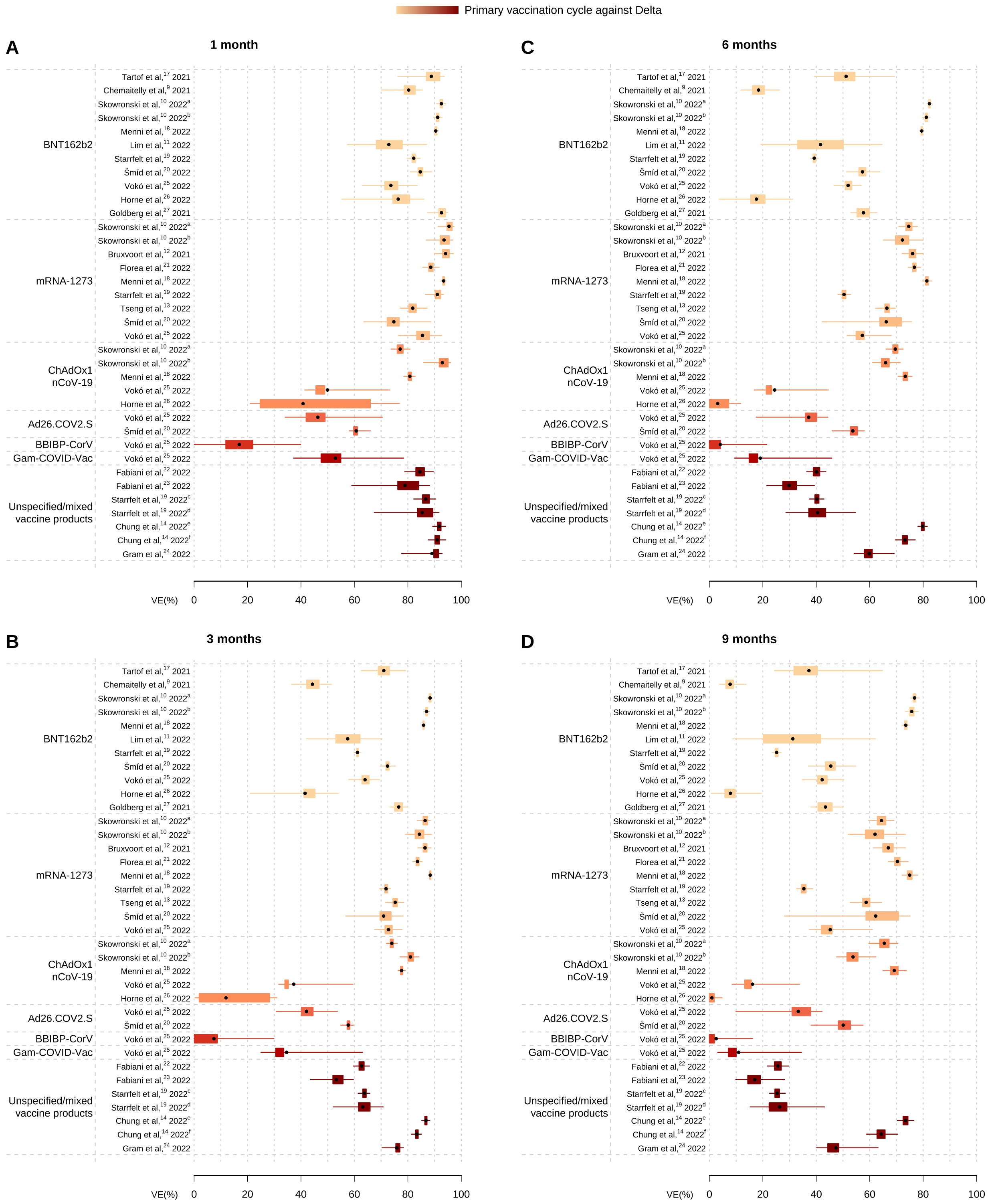

eFigure 9. Effectiveness Over Time of Primary Vaccination Cycle Against Any Delta Laboratory-Confirmed Infection for Single Time Series. Comparison of VE against any laboratory-confirmed SARS-CoV-2 infection with Delta across different vaccine products at 1 (**A**), 3 (**B**), 6 (**C**), and 9 (**D**) months from the administration of primary vaccination cycle for single time series. Points: mean estimates; boxes: interquartile ranges; whiskers: 95% CIs. [a]Data from British Columbia; [b]Data from Quebec; [c]any mRNA vaccine product; [d]any vaccine among BNT162b2, mRNA-1273 and ChAdOx1 nCov-19; [e]any 2-dose mRNA schedule, including BNT162b2/BNT162b2, mRNA-1273/mRNA-1273, and BNT162b2/mRNA-1273; [f]any ChAdOx1 nCov-19-containing schedule, including ChAdOx1 nCov-19/ChAdOx1 nCov-19, ChAdOx1 nCov-19/BNT162b2, and ChAdOx1 nCov-19/mRNA-1273.

© 2023 Menegale F et al. *JAMA Network Open.*

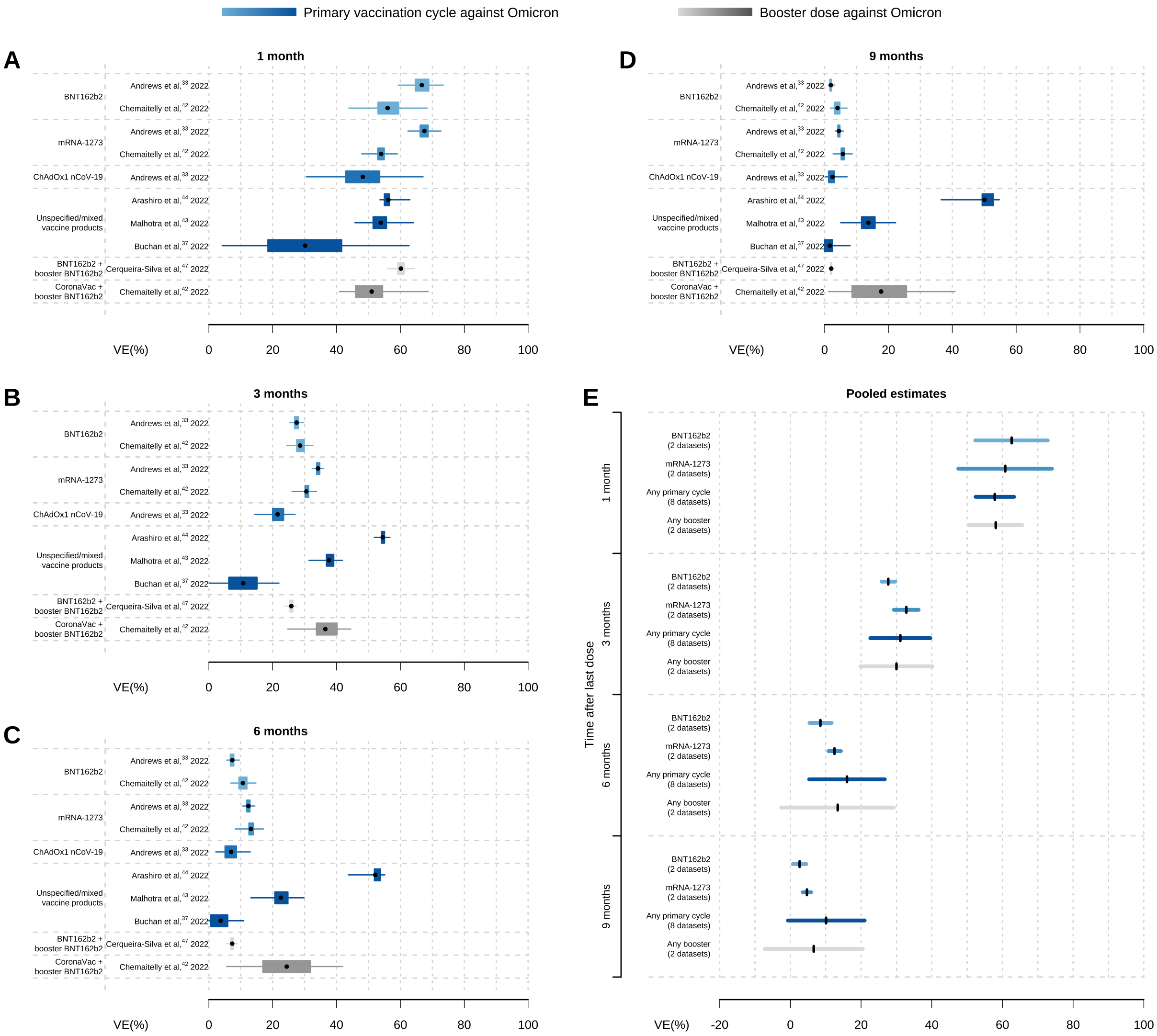

eFigure 10. Effectiveness Over Time of Primary Vaccination Cycle and Booster Vaccination Against Omicron Symptomatic Disease According to Sensitivity Analysis SA1. Comparison of VE against symptomatic disease with Omicron across different vaccine products at 1, 3, 6, and 9 months from the administration of last dose for single time series (**A-D**) and pooled estimates (**E**). Points (single time series) or vertical black lines (pooled estimates): mean estimates; boxes (single time series): interquartile ranges; whiskers: 95% CIs.

© 2023 Menegale F et al. *JAMA Network Open.*

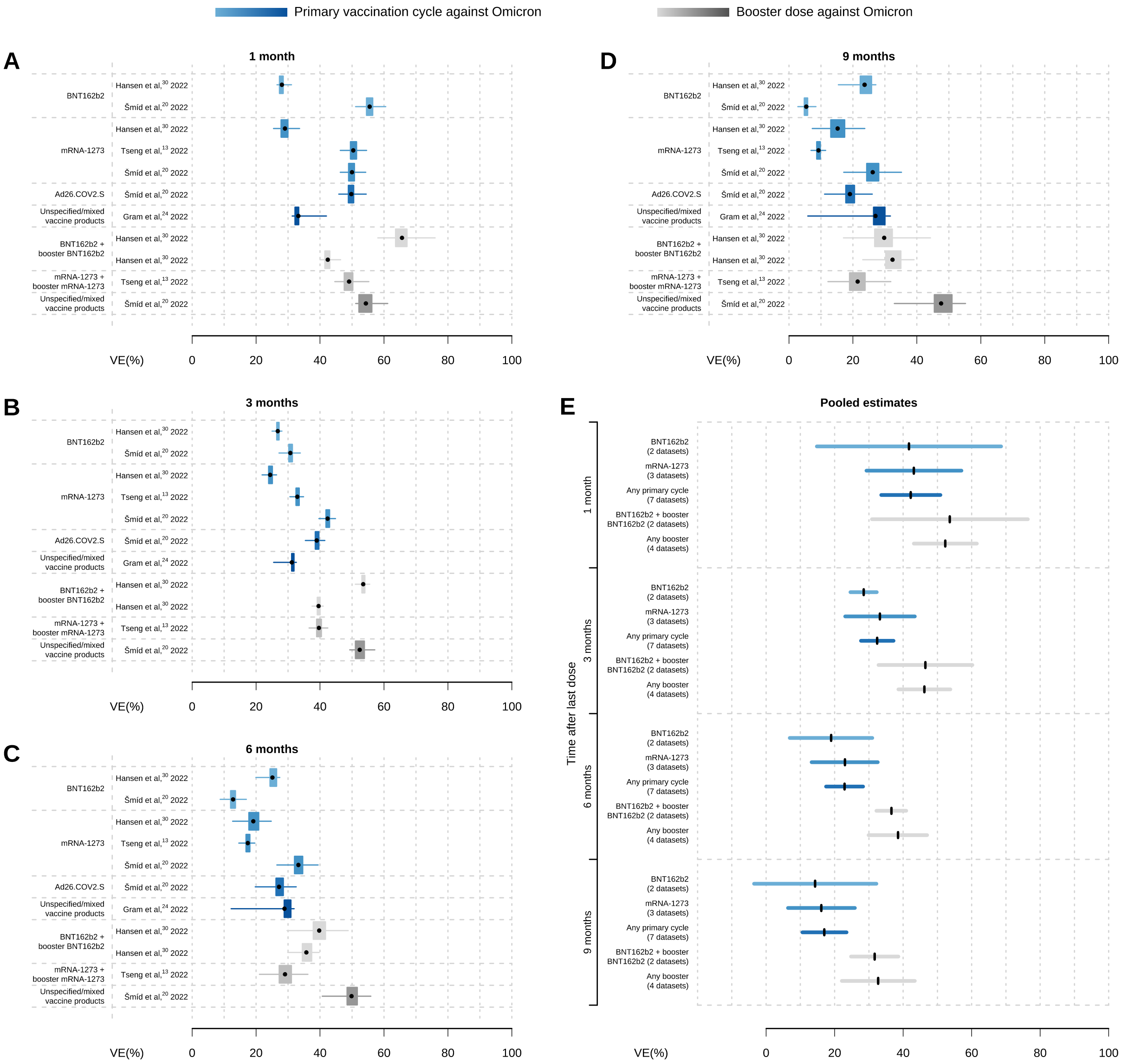

eFigure 11. Effectiveness Over Time of Primary Vaccination Cycle and Booster Vaccination Against Any Omicron Laboratory-Confirmed Infection According to Sensitivity Analysis SA1. Comparison of VE against any laboratory-confirmed SARS-CoV-2 infection with Omicron across different vaccine products at 1, 3, 6, and 9 months from the administration of last dose for single time series (**A-D**) and pooled estimates (**E**). Points (single time series) or vertical black lines (pooled estimates): mean estimates; boxes (single time series): interquartile ranges; whiskers: 95% CIs.

© 2023 Menegale F et al. *JAMA Network Open.*

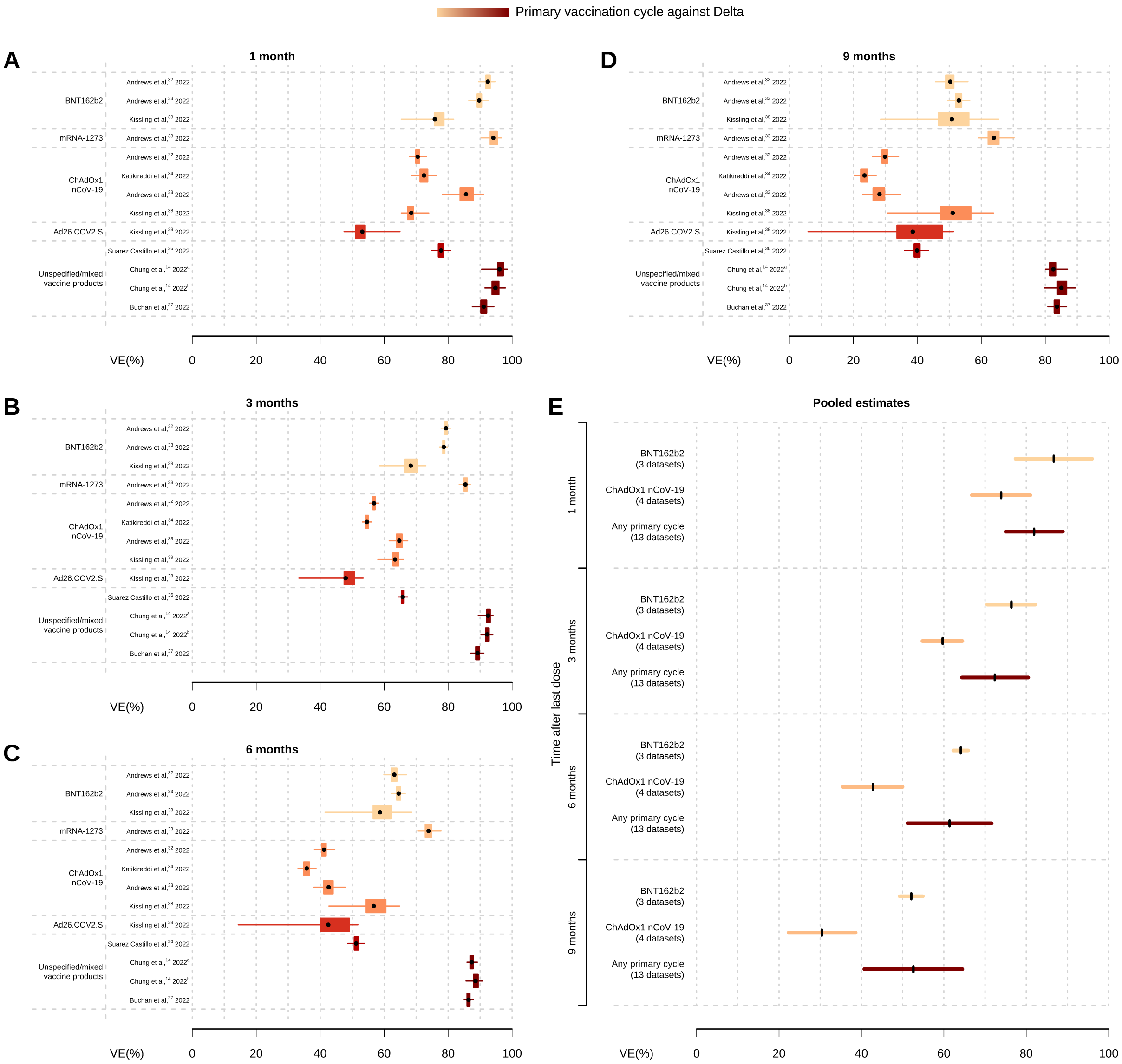

eFigure 12. Effectiveness Over Time of Primary Vaccination Cycle Against Delta Symptomatic Disease According to Sensitivity Analysis SA1. Comparison of VE against symptomatic disease with Delta across different vaccine products at 1, 3, 6, and 9 months from the administration of primary vaccination cycle for single time series (**A-D**) and pooled estimates (**E**). Points (single time series) or vertical black lines (pooled estimates): mean estimates; boxes (single time series): interquartile ranges; whiskers: 95% CIs. [a] Any 2-dose mRNA schedule, including BNT162b2/BNT162b2, mRNA-1273/mRNA-1273, and BNT162b2/mRNA-1273; [b] Any ChAdOx1 nCov-19-containing schedule, including ChAdOx1 nCov-19/ChAdOx1 nCov-19, ChAdOx1 nCov-19/BNT162b2, and ChAdOx1 nCov-19/mRNA-1273.

© 2023 Menegale F et al. *JAMA Network Open.*

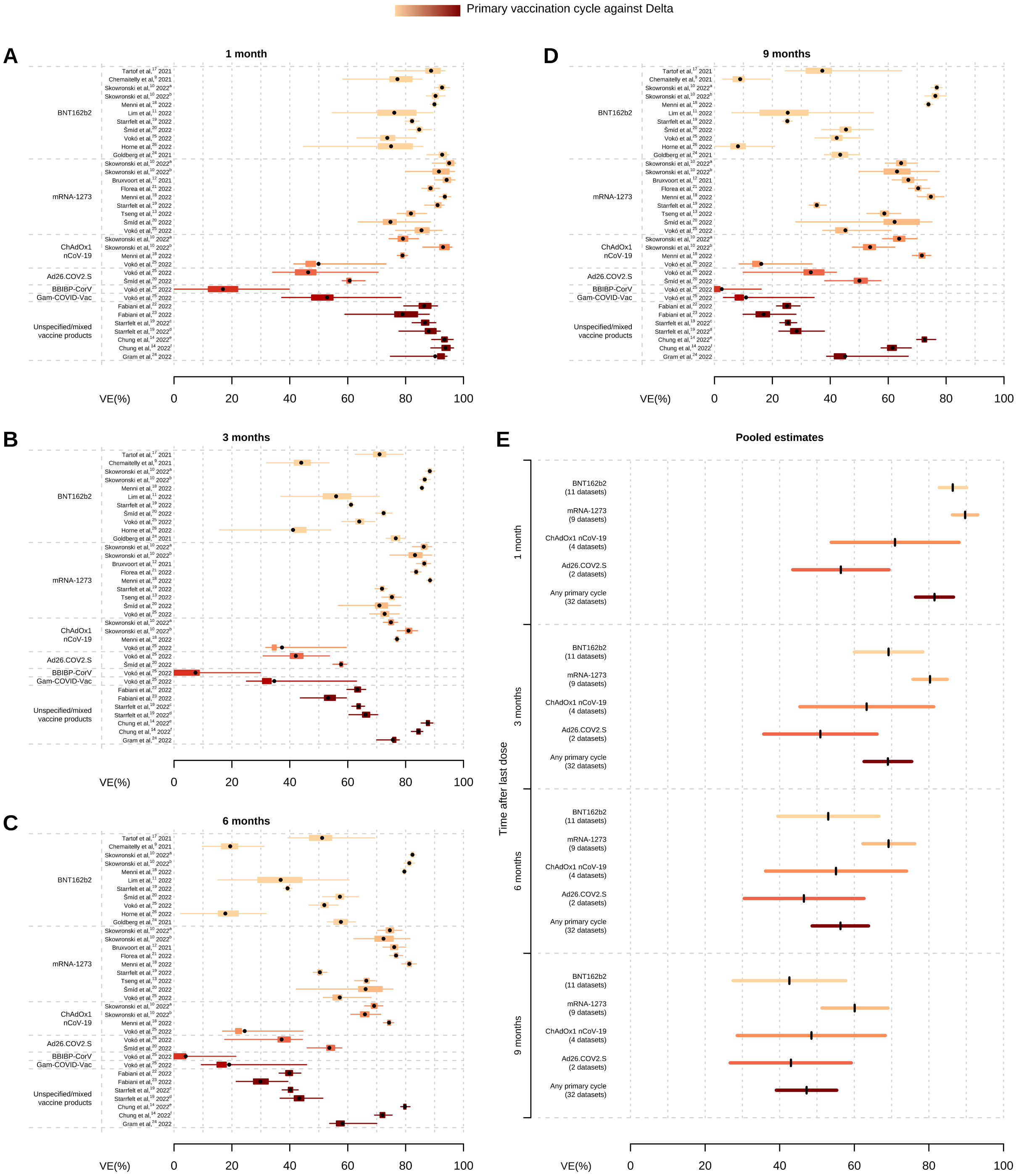

eFigure 13. Effectiveness Over Time of Primary Vaccination Cycle Against Any Delta Laboratory-Confirmed Infection According to Sensitivity Analysis SA1. Comparison of VE against any laboratory-confirmed SARS-CoV-2 infection with Delta across different vaccine products at 1, 3, 6, and 9 months from the administration of primary vaccination cycle for single time series (**A-D**) and pooled estimates (**E**). Points (single time series) or vertical black lines (pooled estimates): mean estimates; boxes (single time series): interquartile ranges; whiskers: 95% CIs. [a] Data from British Columbia; [b] Data from Quebec; [c] any mRNA vaccine product; [d] any vaccine among BNT162b2, mRNA-1273 and ChAdOx1 nCov-19; [e] any 2-dose mRNA schedule, including BNT162b2/BNT162b2, mRNA-1273/mRNA-1273, and BNT162b2/mRNA-1273; [f] any ChAdOx1 nCov-19-containing schedule, including ChAdOx1 nCov-19/ChAdOx1 nCov-19, ChAdOx1 nCov-19/BNT162b2, and ChAdOx1 nCov-19/mRNA-1273.

© 2023 Menegale F et al. *JAMA Network Open.*

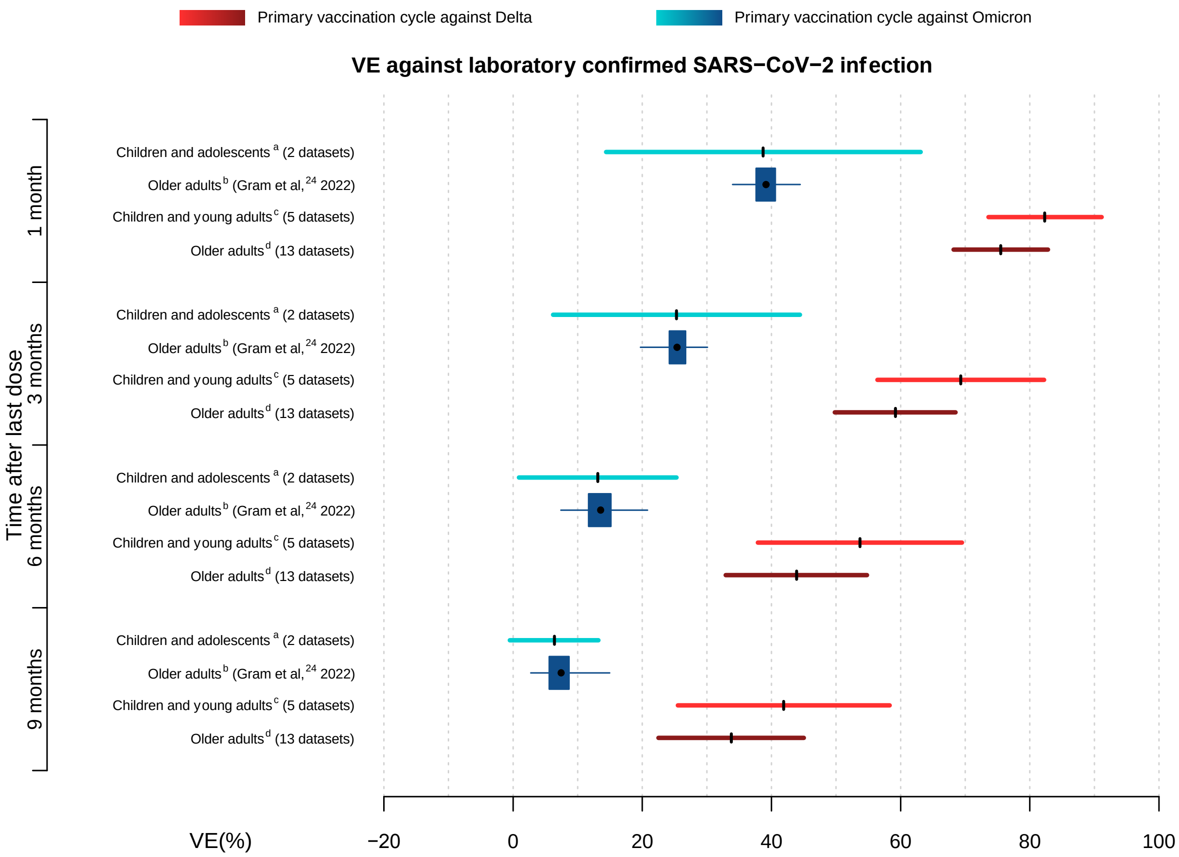

eFigure 14. Effectiveness Over Time of Primary Vaccination Cycle Against Any Omicron and Delta Laboratory-Confirmed Infection for Young and Elderly Individuals. Comparison of VE against any laboratory-confirmed SARS-CoV-2 infection with Omicron and Delta across different vaccine products at 1, 3, 6, and 9 months from the administration of primary vaccination cycle for young and elderly individuals. Points (single time series) or vertical black lines (pooled estimates): mean estimates; boxes (single time series): interquartile ranges; whiskers: 95% CIs. [a] <18 years-old, vaccinated with BNT162b2; [b] >60 years-old, vaccinated with any mRNA vaccine; [c] <25 years-old, vaccinated with different vaccine products (BNT162b2, mRNA-1273); [d] >60 years-old, vaccinated with different vaccine products (BNT162b2, mRNA-1273, ChAdOx1 nCoV-19, Ad26.COV2.S, BBIBP-CorV, Gam-COVID-Vac, unspecified/mixed products).

© 2023 Menegale F et al. *JAMA Network Open.*

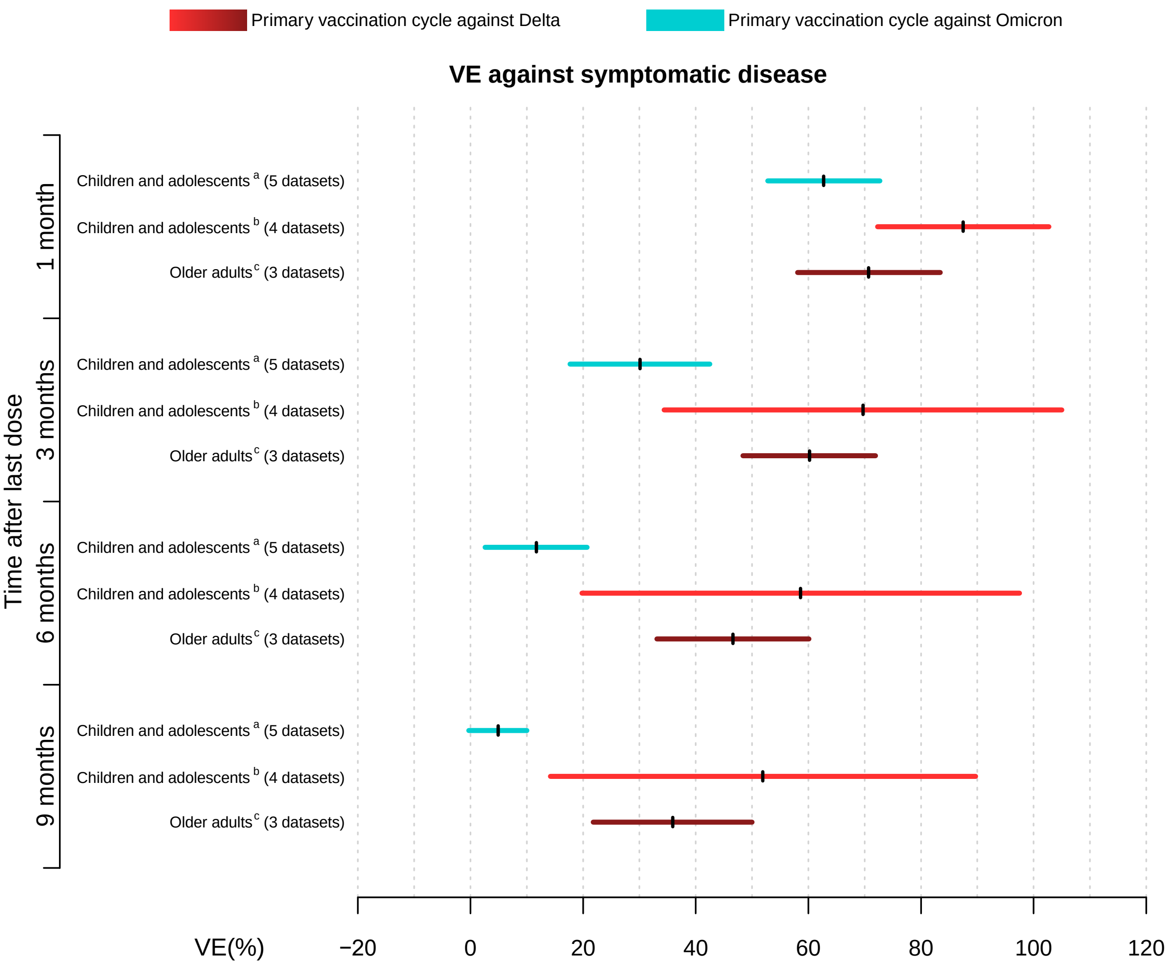

eFigure 15. Effectiveness Over Time of Primary Vaccination Cycle Against Omicron and Delta Symptomatic Disease for Young and Elderly Individuals. Comparison of VE against symptomatic disease with Omicron and Delta across different vaccine products at 1, 3, 6, and 9 months from the administration of primary vaccination cycle for young and elderly individuals. Vertical black lines: mean estimates; whiskers: 95% CIs. [a] <18 years-old, vaccinated with BNT162b2; [b] <25 years-old, vaccinated with BNT162b2; [c] >60 years-old, vaccinated with different vaccine products (BNT162b2, ChAdOx1 nCoV-19).

© 2023 Menegale F et al. *JAMA Network Open.*

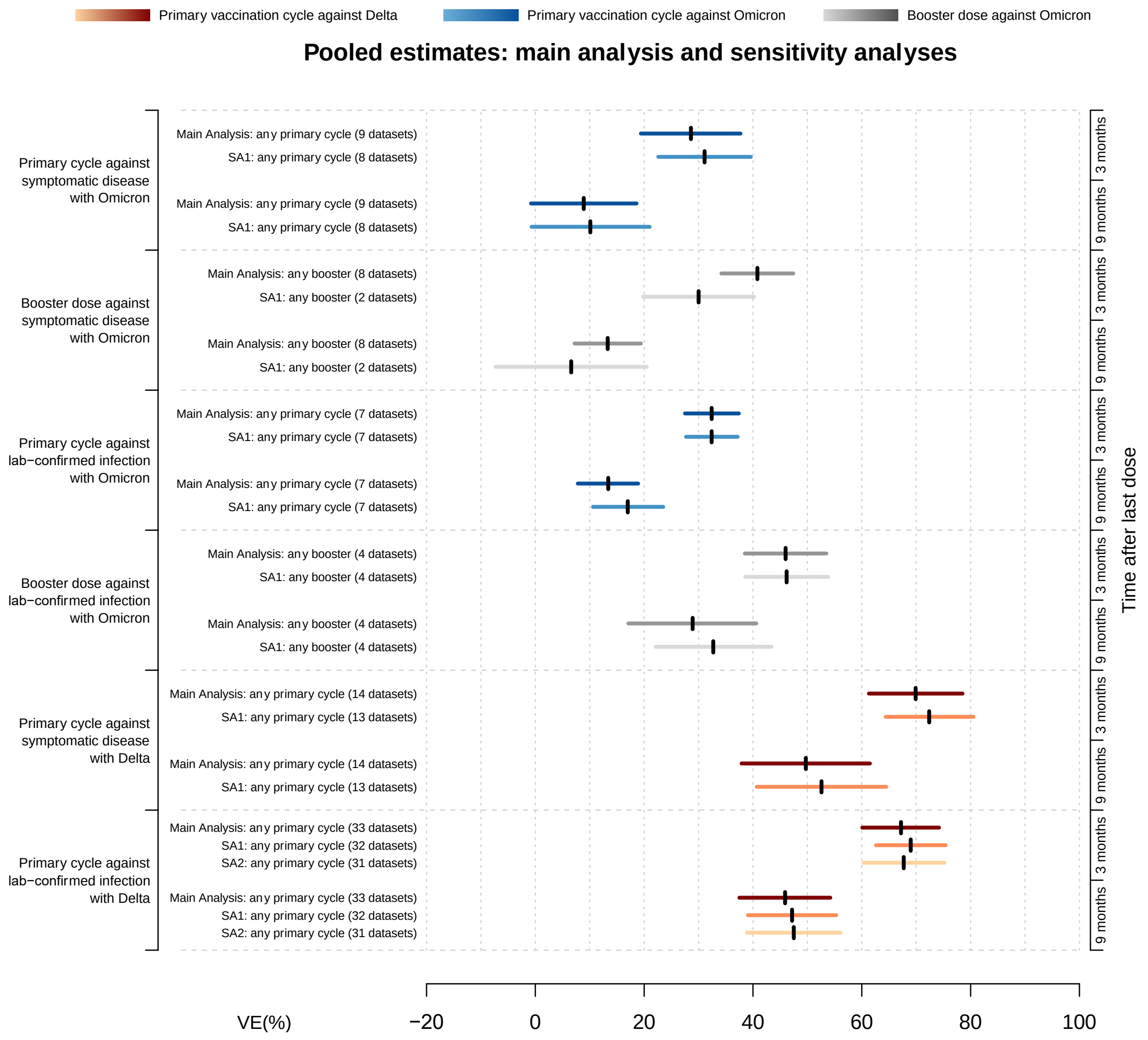

eFigure 16. Comparison of Vaccine Effectiveness Resulting From Main Analysis and Sensitivity Analyses. Comparison of VE against symptomatic disease and laboratory-confirmed SARS-CoV-2 infection with Omicron and Delta at 1, 3, 6, and 9 months from last dose administration according to main analysis and sensitivity analyses SA1 (estimating VE from data points from the original studies where VE was estimated at least 30 days after the administration of the last dose or data points that include observations in a period of at least 60 days after the administration of the last dose) and SA2 (excluding studies where VE was estimated by assuming that individuals who have received a single dose not earlier than 14 days represent a proxy for unvaccinated individuals). Points (single time series) or vertical black lines (pooled estimates): mean estimates; boxes (single time series): interquartile ranges; whiskers: 95% CIs.

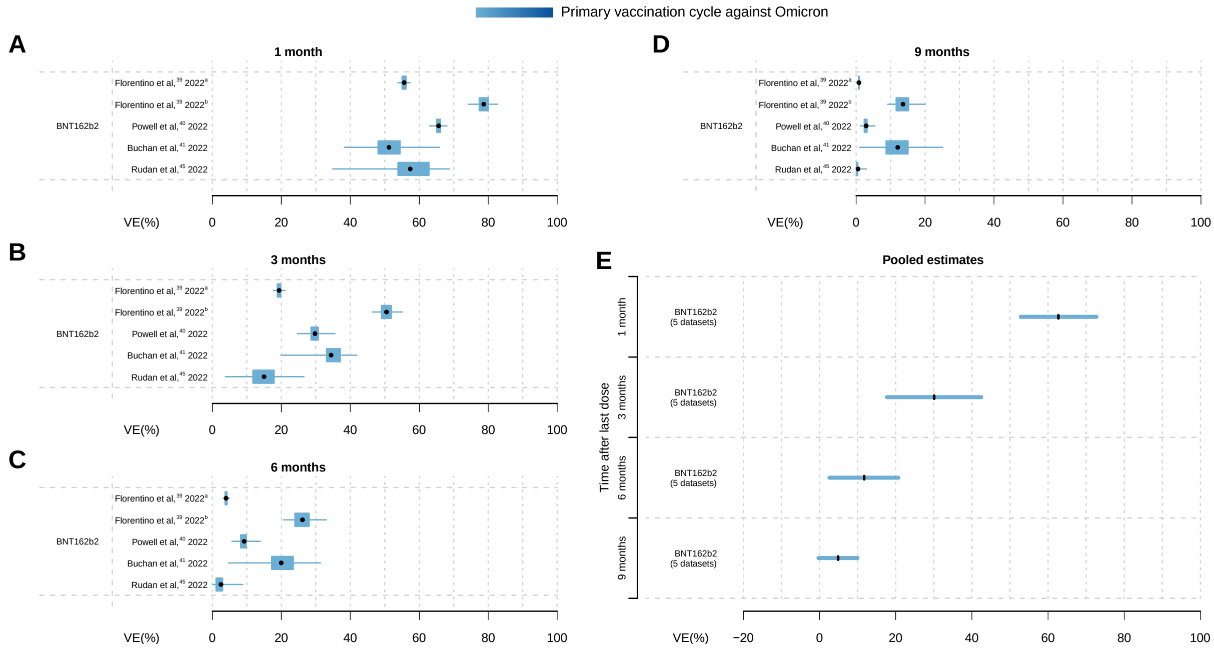

© 2023 Menegale F et al. *JAMA Network Open.*

eFigure 17. Effectiveness Over Time of Primary Vaccination Cycle Against Omicron Symptomatic Disease for Young Individuals. Comparison of VE against symptomatic disease with Omicron for young (<25 y-o) individuals across different vaccine products at 1, 3, 6, and 9 months from the administration of last dose for single time series (**A-D**) and pooled estimates (**E**). Points (single time series) or vertical black lines (pooled estimates): mean estimates; boxes (single time series): interquartile ranges; whiskers: 95% CIs. [a] Data from Brazil; [b] Data from Scotland.

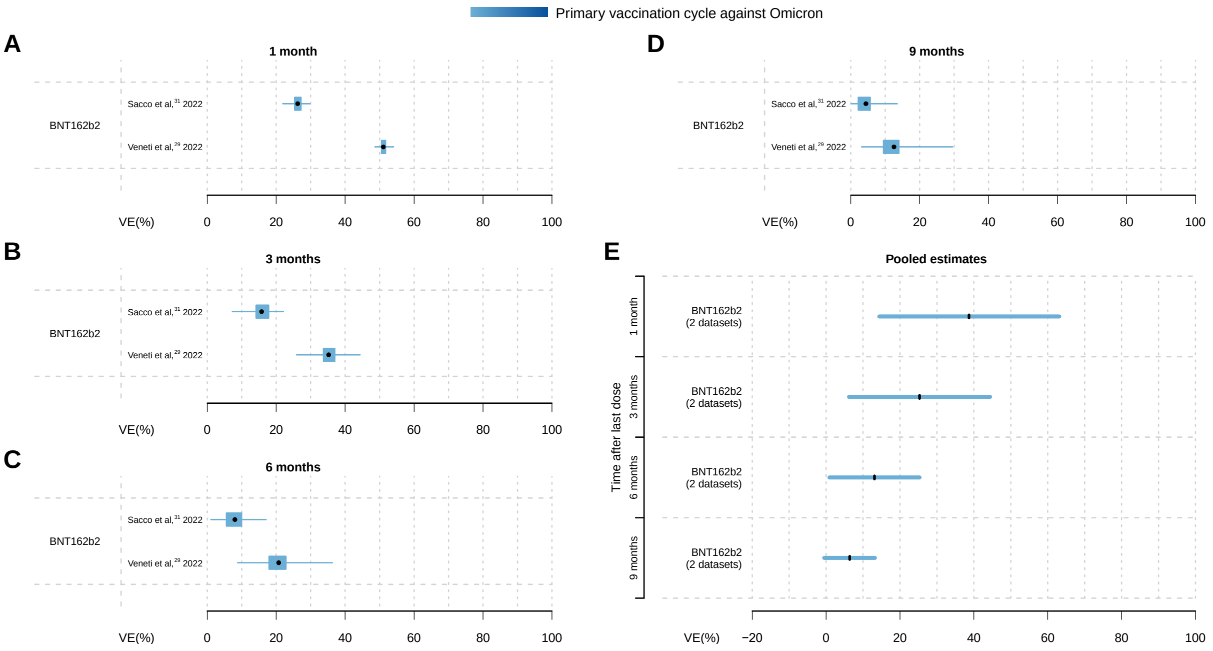

eFigure 18. Effectiveness Over Time of Primary Vaccination Cycle Against Any Omicron Laboratory-Confirmed Infection for Young Individuals. Comparison of VE against any laboratory-confirmed SARS-CoV-2 infection with Omicron for young (<25 y-o) individuals across different vaccine products at 1, 3, 6, and 9 months from the administration of last dose for single time series (**A-D**) and pooled estimates (**E**). Points (single time series) or vertical black lines (pooled estimates): mean estimates; boxes (single time series): interquartile ranges; whiskers: 95% CIs.

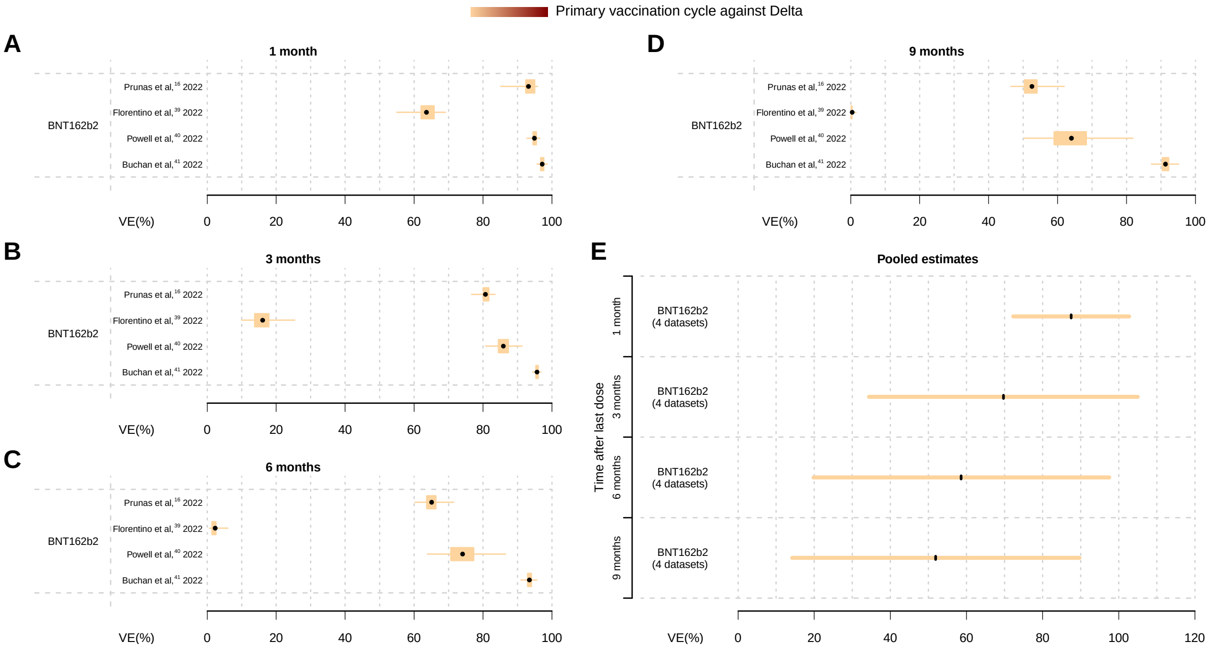

eFigure 19. Effectiveness Over Time of Primary Vaccination Cycle Against Delta Symptomatic Disease for Young Individuals. Comparison of VE against symptomatic disease with Delta for young (<25 y-o) individuals across different vaccine products at 1, 3, 6, and 9 months from the administration of last dose for single time series (**A-D**) and pooled estimates (**E**). Points (single time series) or vertical black lines (pooled estimates): mean estimates; boxes (single time series): interquartile ranges; whiskers: 95% CIs.

© 2023 Menegale F et al. *JAMA Network Open.*

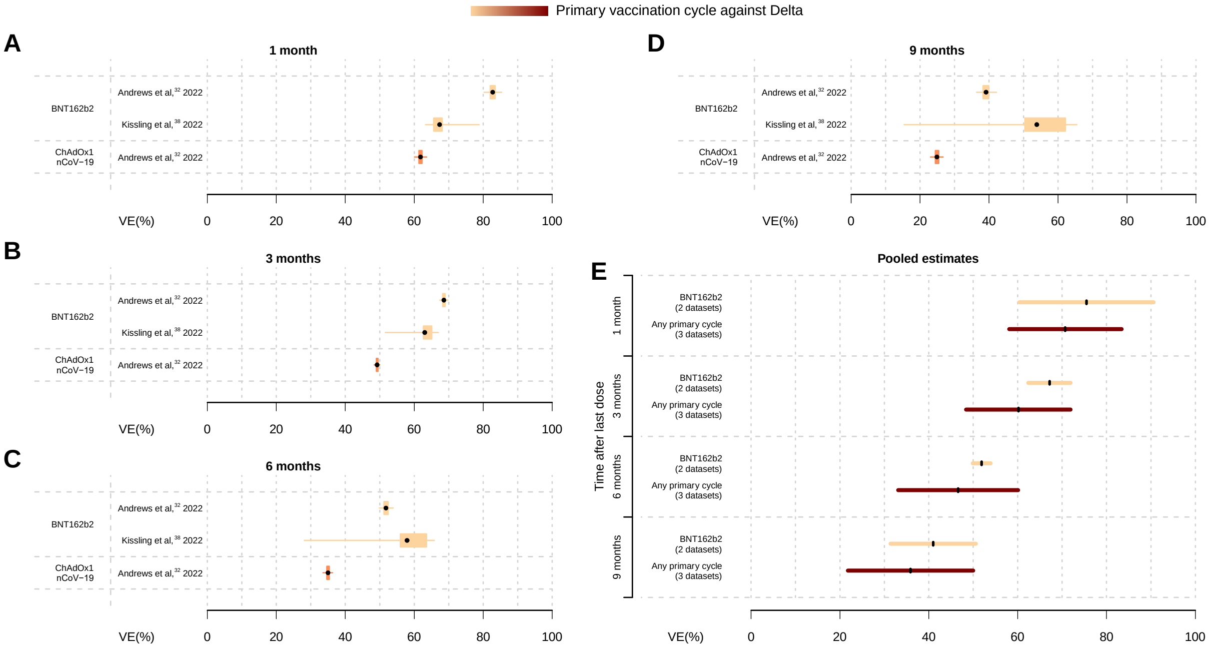

eFigure 20. Effectiveness Over Time of Primary Vaccination Cycle Against Delta Symptomatic Disease for Elderly Individuals. Comparison of VE against symptomatic disease with Delta for elderly (>60 y-o) individuals across different vaccine products at 1, 3, 6, and 9 months from the administration of last dose for single time series (**A-D**) and pooled estimates (**E**). Points (single time series) or vertical black lines (pooled estimates): mean estimates; boxes (single time series): interquartile ranges; whiskers: 95% CIs.

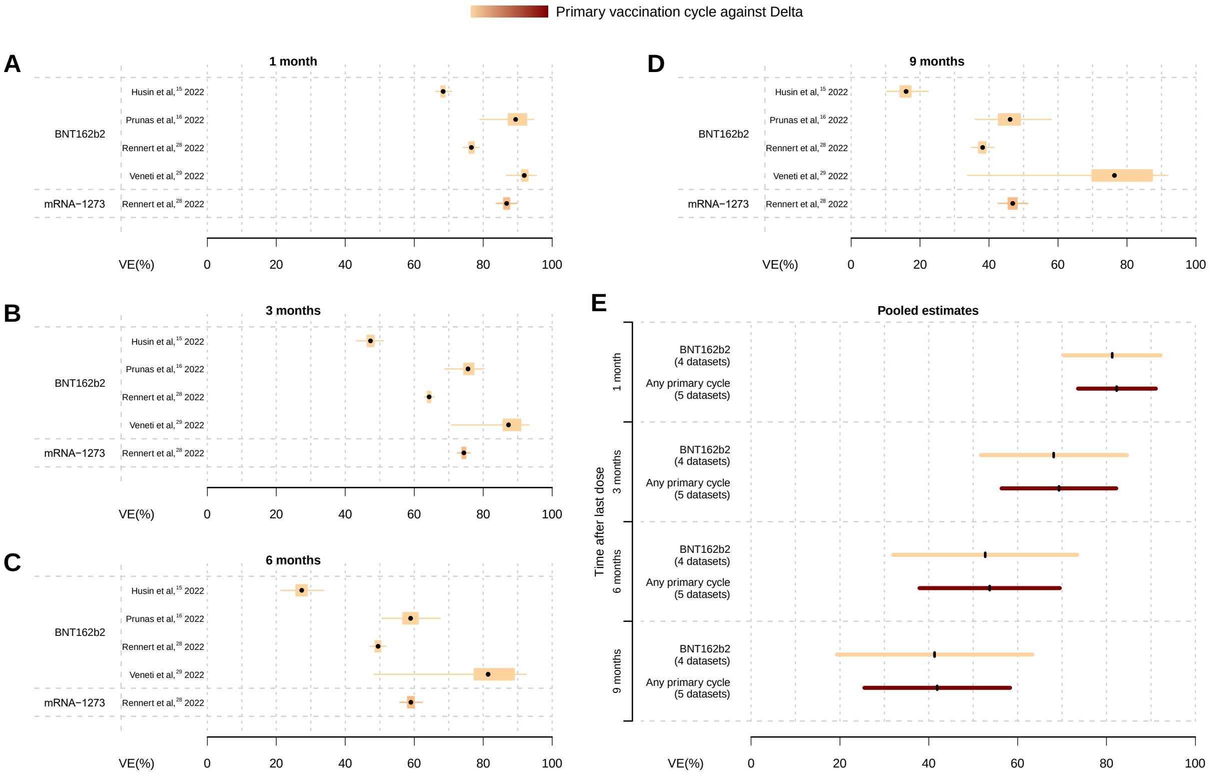

eFigure 21. Effectiveness Over Time of Primary Vaccination Cycle Against Any Delta Laboratory-Confirmed Infection for Young Individuals. Comparison of VE against any laboratory-confirmed SARS-CoV-2 infection with Delta for young (<25 y-o) individuals across different vaccine products at 1, 3, 6, and 9 months from the administration of last dose for single time series (**A-D**) and pooled estimates (**E**). Points (single time series) or vertical black lines (pooled estimates): mean estimates; boxes (single time series): interquartile ranges; whiskers: 95% CIs.

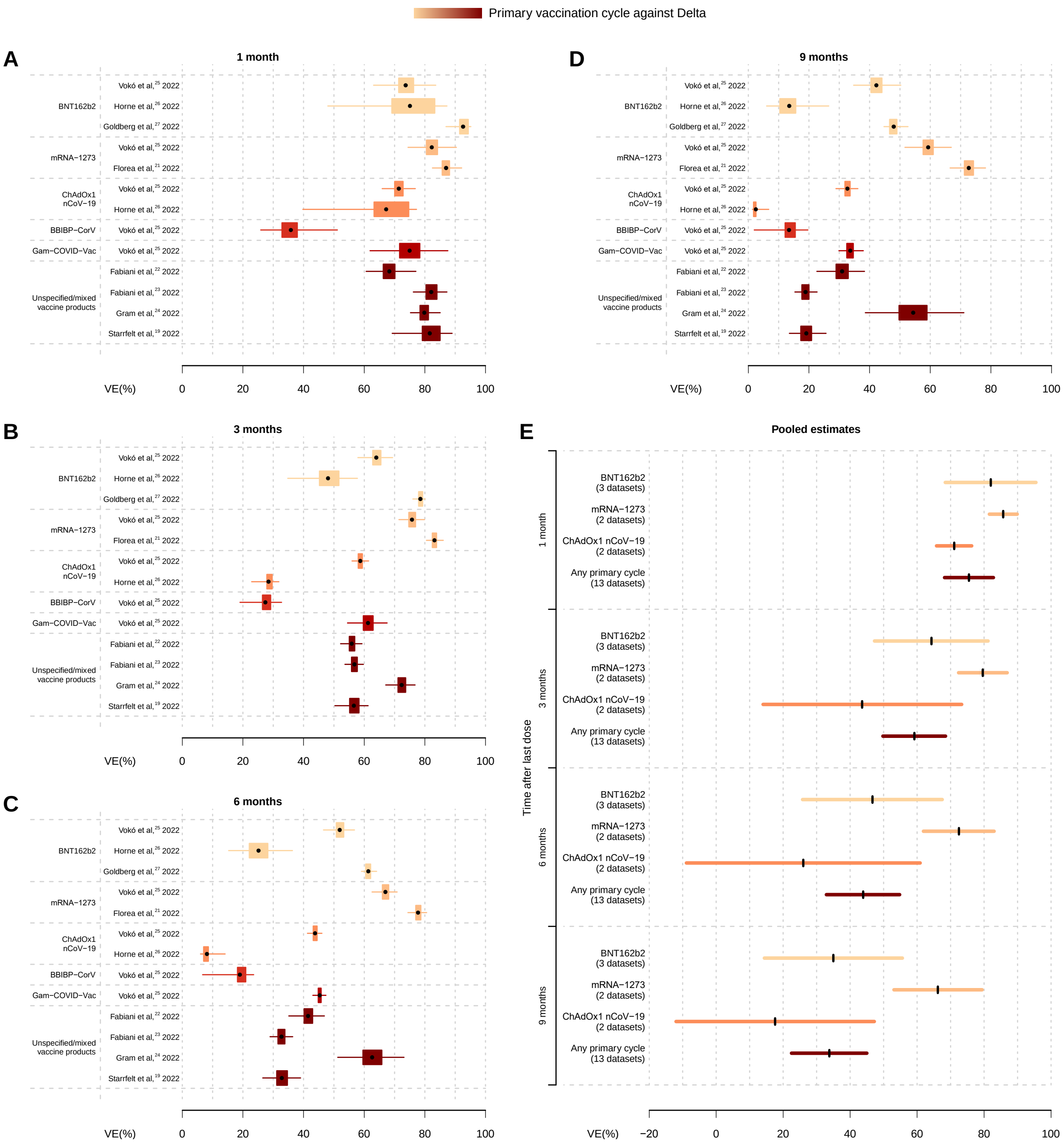

eFigure 22. Effectiveness Over Time of Primary Vaccination Cycle Against Any Delta Laboratory-Confirmed Infection for Elderly Individuals. Comparison of VE against any laboratory-confirmed SARS-CoV-2 infection with Delta for elderly (>60 y-o) individuals across different vaccine products at 1, 3, 6, and 9 months from the administration of last dose for single time series (**A-D**) and pooled estimates (**E**). Points (single time series) or vertical black lines (pooled estimates): mean estimates; boxes (single time series): interquartile ranges; whiskers: 95% CIs.

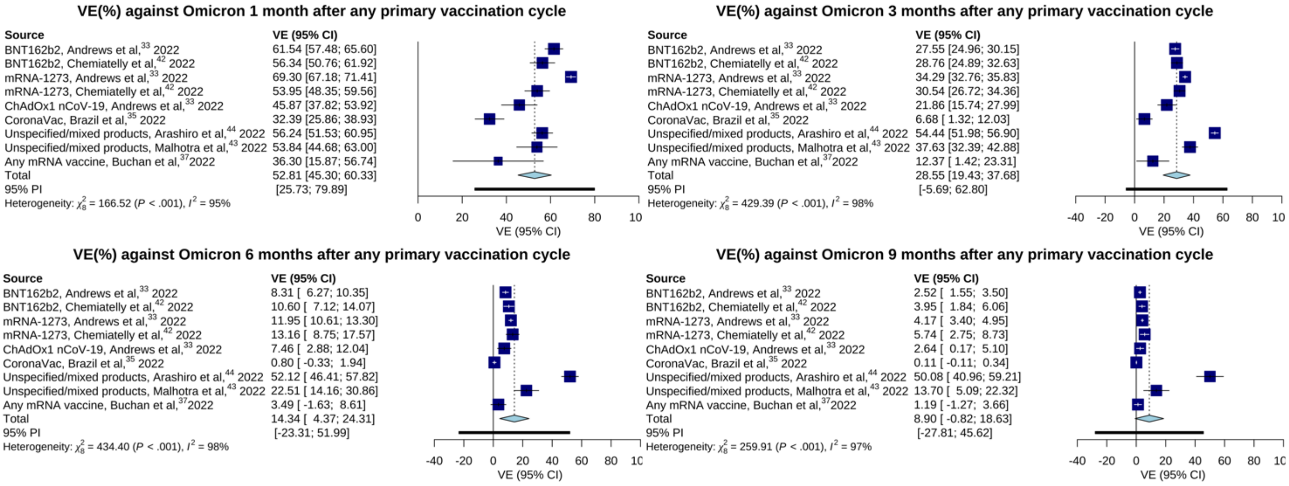

eFigure 23. Pooled Estimates of VE Against Symptomatic Disease With Omicron at 1, 3, 6, and 9 Months After Any Primary Vaccination Cycle.

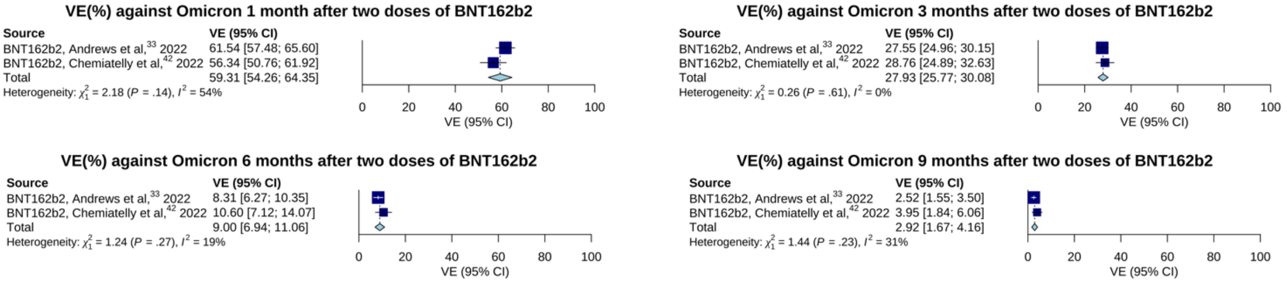

eFigure 24. Pooled Estimates of VE Against Symptomatic Disease With Omicron at 1, 3, 6, and 9 Months After Two Doses of BNT162b2.

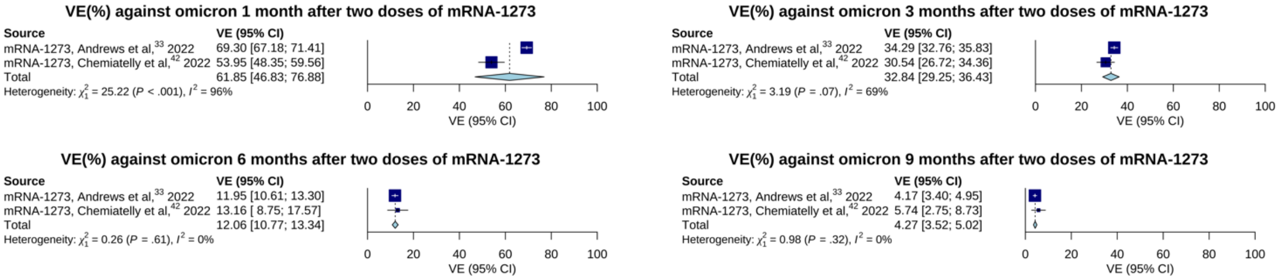

eFigure 25. Pooled Estimates of VE Against Symptomatic Disease With Omicron at 1, 3, 6, and 9 Months After Two Doses of mRNA-1273.

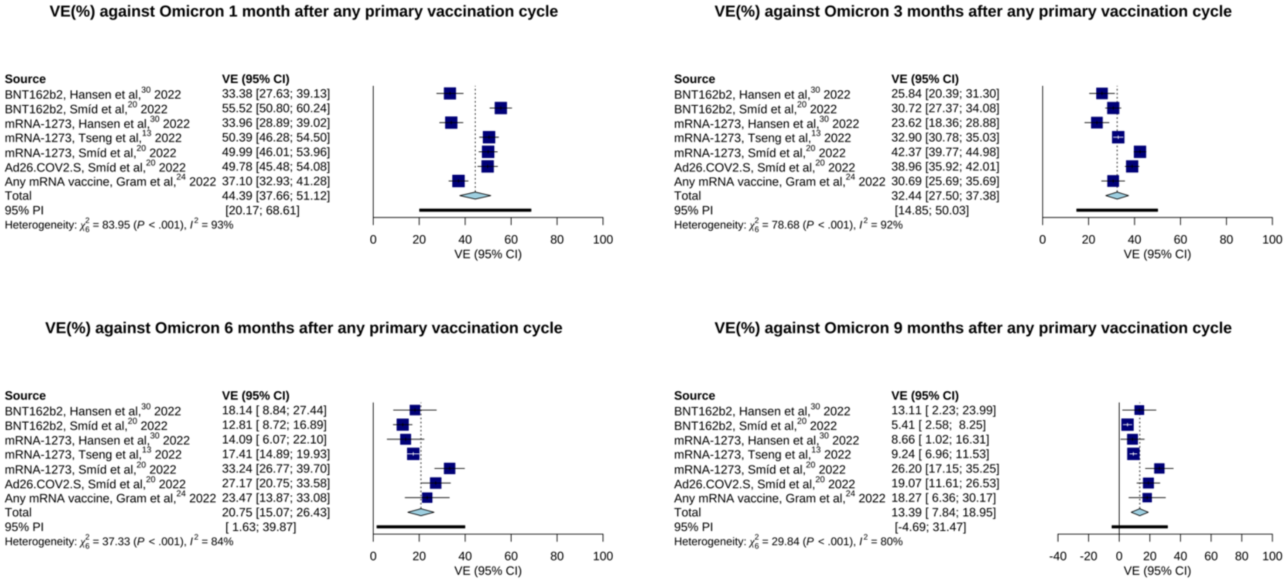

eFigure 26. Pooled Estimates of VE Against Any Laboratory-Confirmed Infection With Omicron at 1, 3, 6, and 9 Months After Any Primary Vaccination Cycle.

© 2023 Menegale F et al. *JAMA Network Open.*

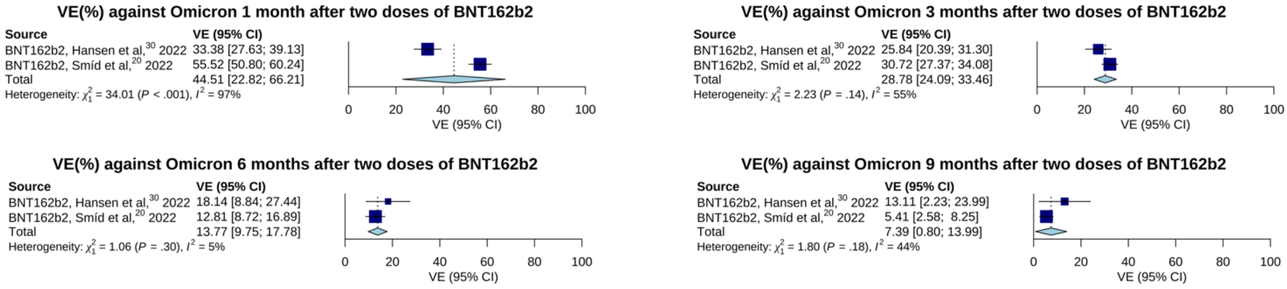

eFigure 27. Pooled Estimates of VE Against Any Laboratory-Confirmed Infection With Omicron at 1, 3, 6, and 9 Months After Two Doses of BNT162b2.

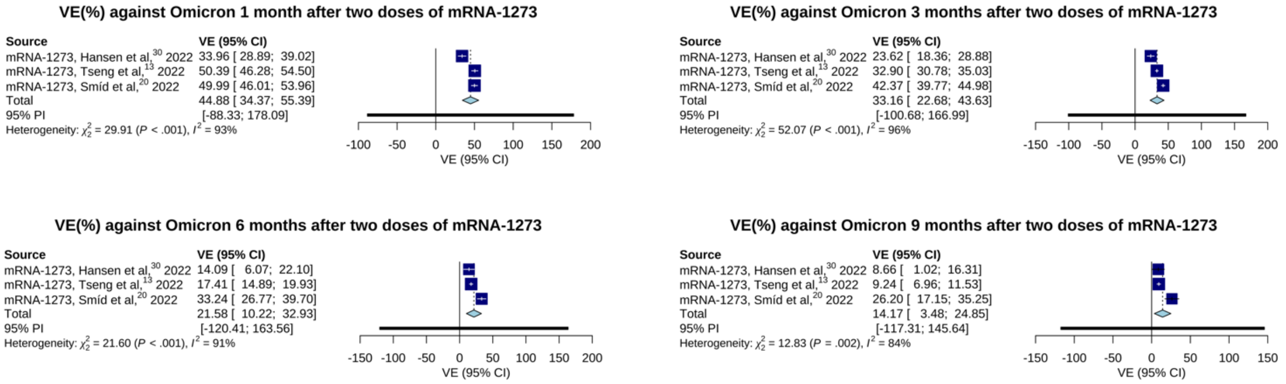

eFigure 28. Pooled Estimates of VE Against Any Laboratory-Confirmed Infection With Omicron at 1, 3, 6, and 9 Months After Two Doses of mRNA- 1273.

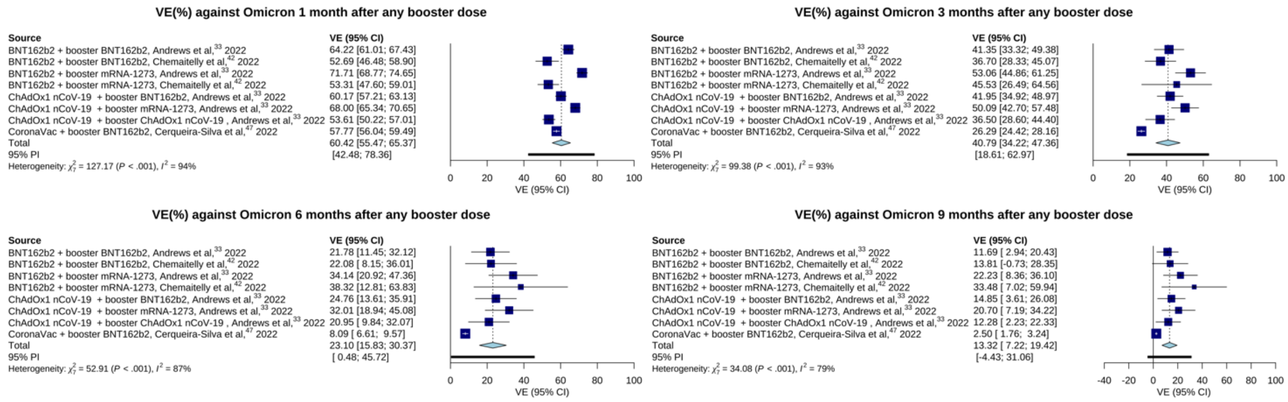

eFigure 29. Pooled Estimates of VE Against Symptomatic Disease With Omicron at 1, 3, 6, and 9 Months After Any Booster Dose.

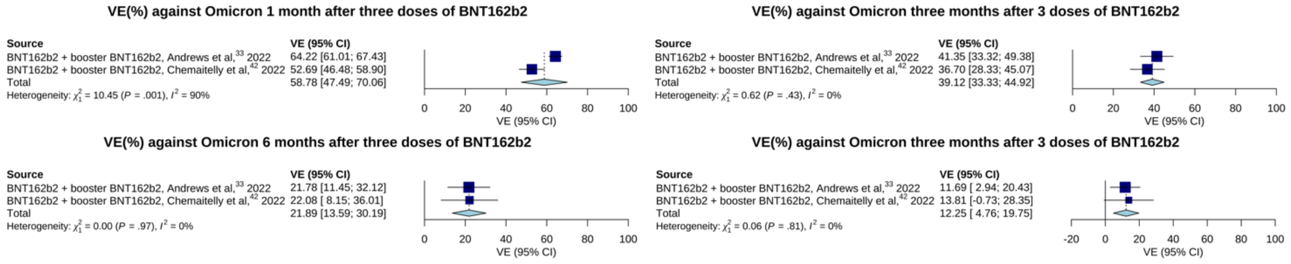

eFigure 30. Pooled Estimates of VE Against Symptomatic Disease With Omicron at 1, 3, 6, and 9 Months After Three Doses of BNT162b2.

© 2023 Menegale F et al. *JAMA Network Open.*

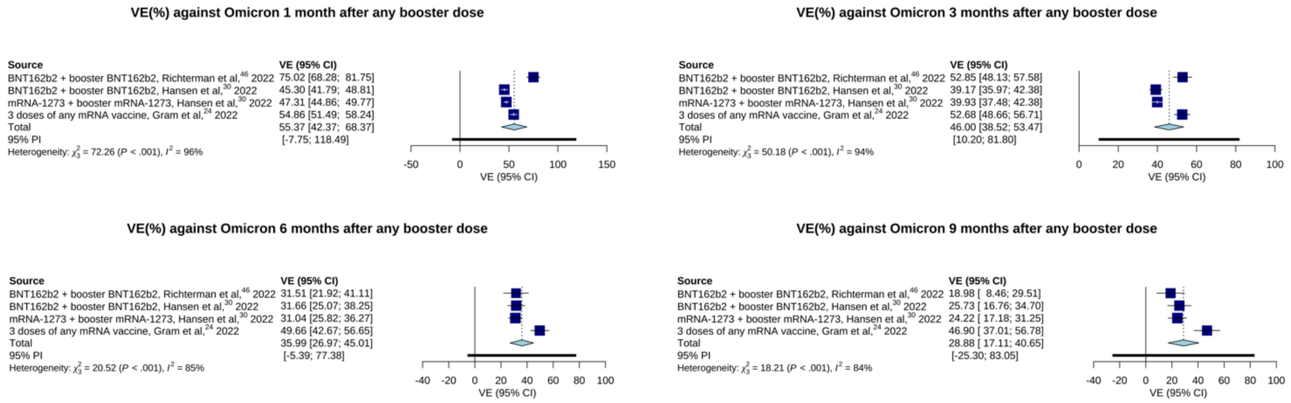

eFigure 31. Pooled Estimates of VE Against Any Laboratory-Confirmed Infection With Omicron at 1, 3, 6, and 9 Months After Any Booster Dose.

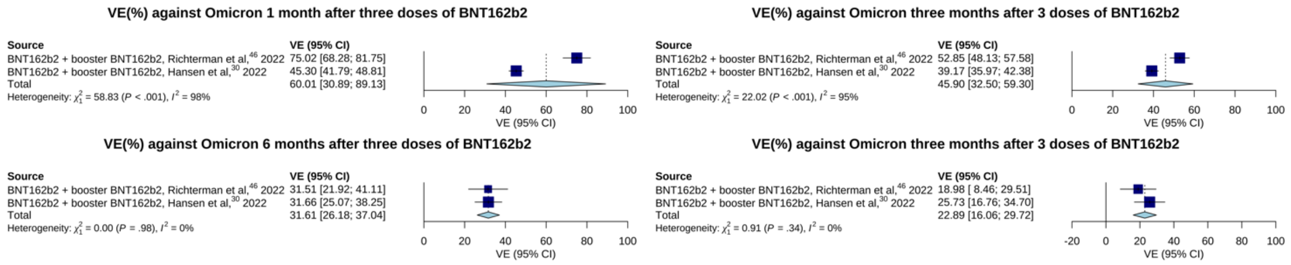

eFigure 32. Pooled Estimates of VE Against Any Laboratory-Confirmed Infection With Omicron at 1, 3, 6, and 9 Months After Three Doses of BNT162b2.

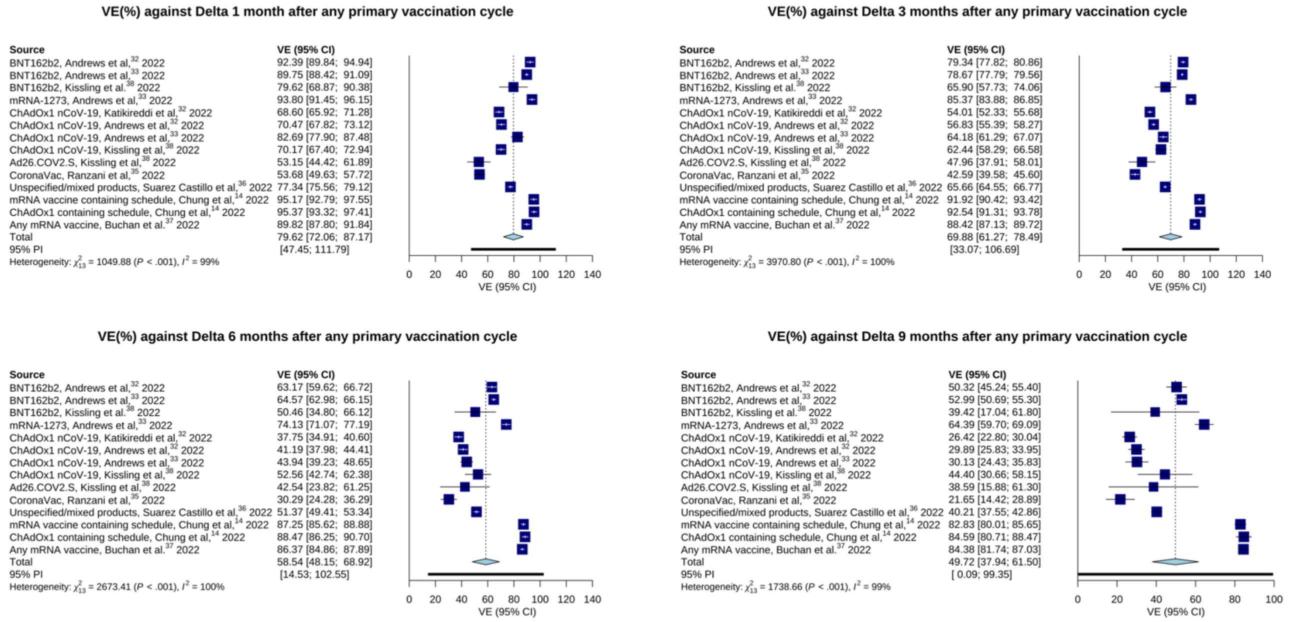

eFigure 33. Pooled Estimates of VE Against Symptomatic Disease With Delta at 1, 3, 6, and 9 Months After Any Primary Vaccination Cycle.

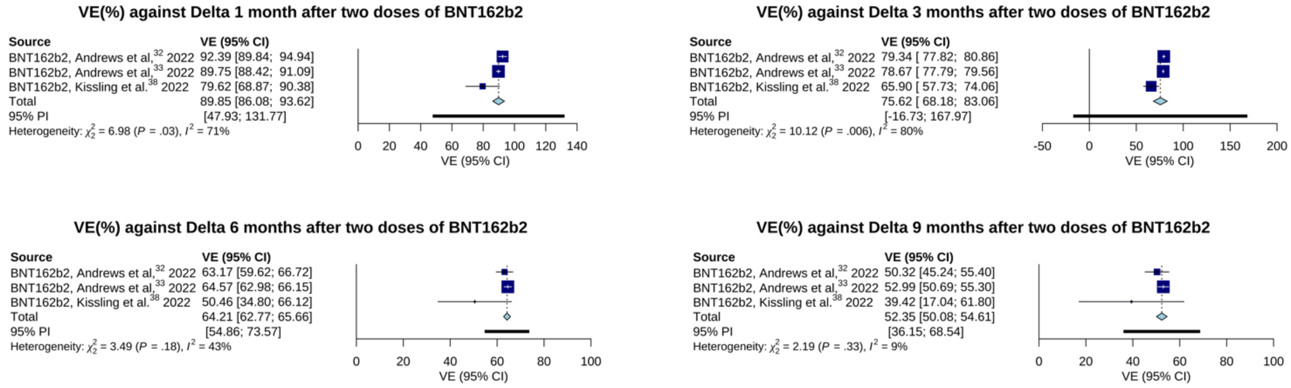

eFigure 34. Pooled Estimates of VE Against Symptomatic Disease With Delta at 1, 3, 6, and 9 Months After Two Doses of BNT162b2.

© 2023 Menegale F et al. *JAMA Network Open.*

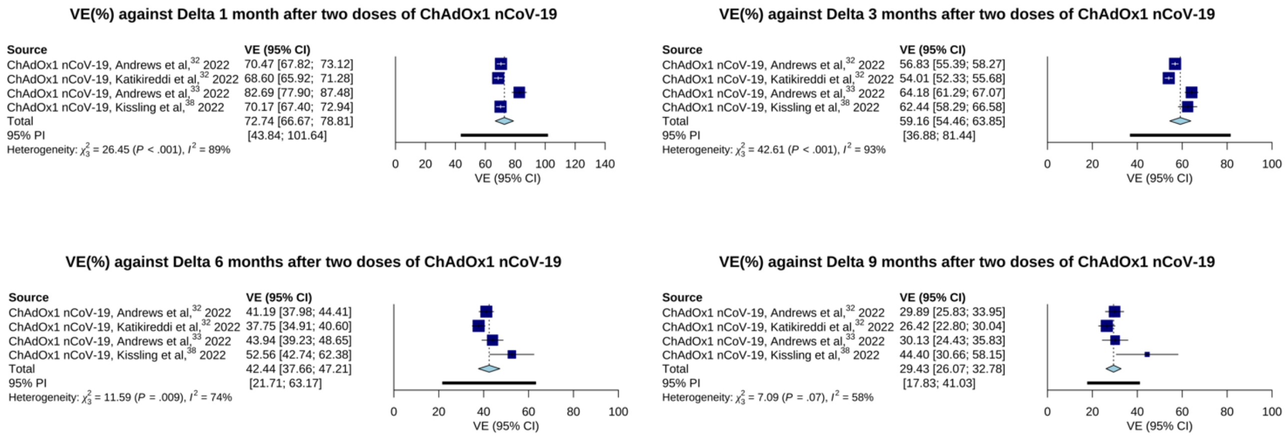

eFigure 35. Pooled Estimates of VE Against Symptomatic Disease With Delta at 1, 3, 6, and 9 Months After Two Doses of ChAdOx1 nCoV-19.

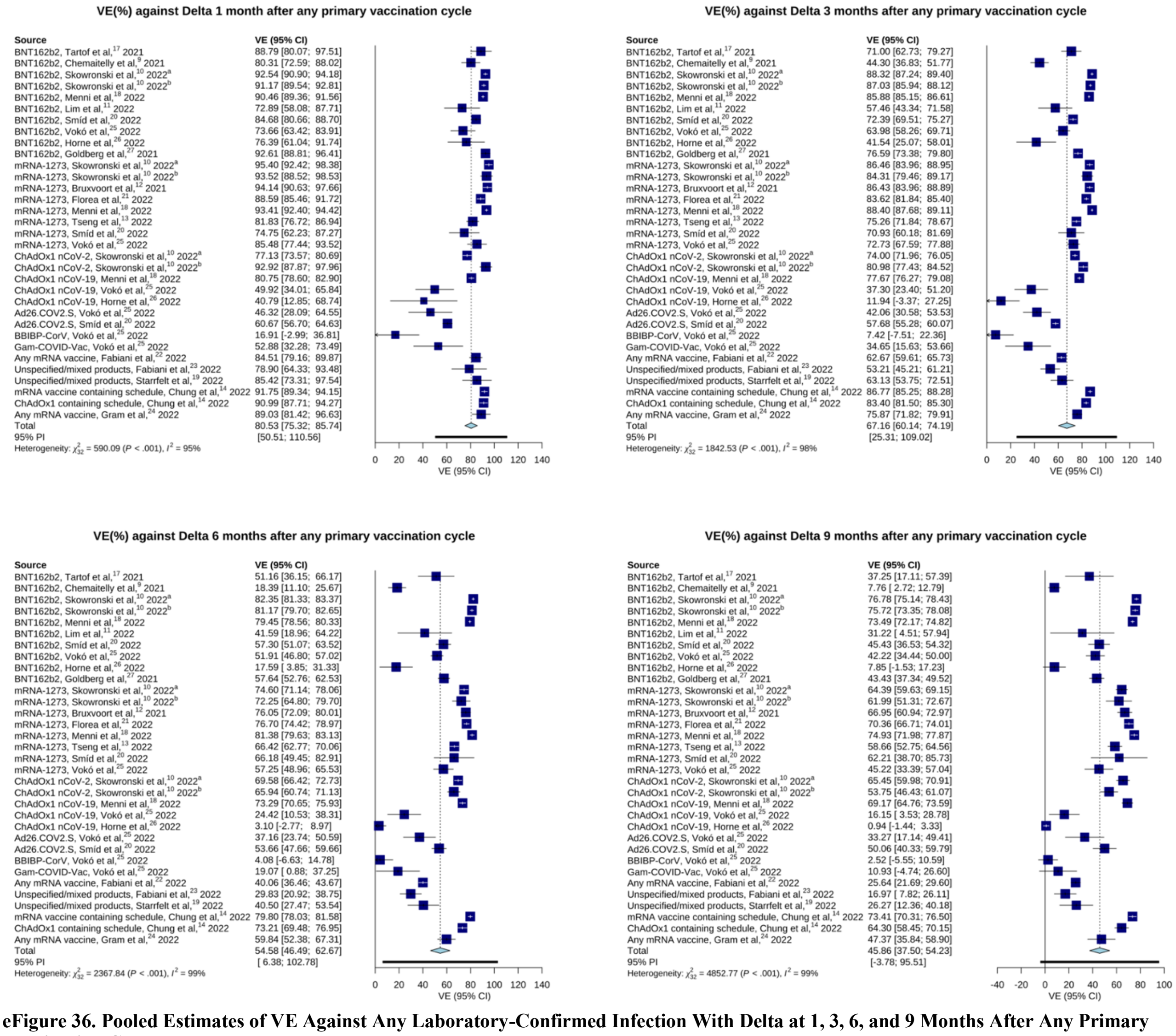

eFigure 36. Pooled Estimates of VE Against Any Laboratory-Confirmed Infection With Delta at 1, 3, 6, and 9 Months After Any Primary Vaccination Cycle.

[a] Data from British Columbia; [b] Data from Quebec.

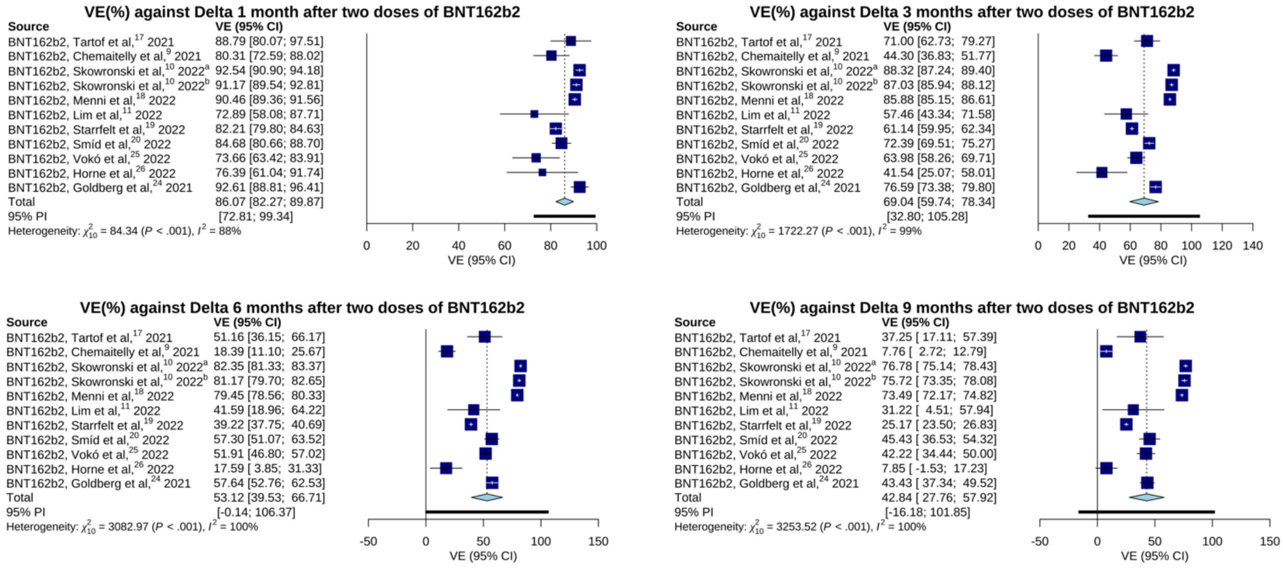

eFigure 37. Pooled Estimates of VE Against Any Laboratory-Confirmed Infection With Delta at 1, 3, 6, and 9 Months After Two Doses of BNT162b2.

[a] Data from British Columbia; [b] Data from Quebec.

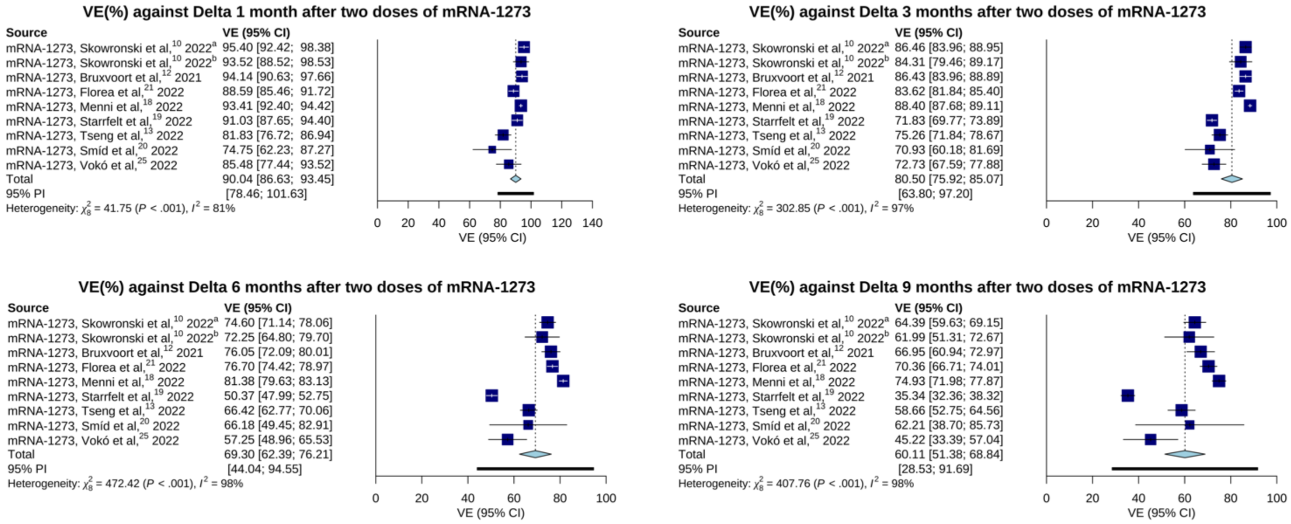

eFigure 38. Pooled Estimates of VE Against Any Laboratory-Confirmed Infection With Delta at 1, 3, 6, and 9 Months After Two Doses of mRNA-1273.

[a] Data from British Columbia; [b] Data from Quebec.

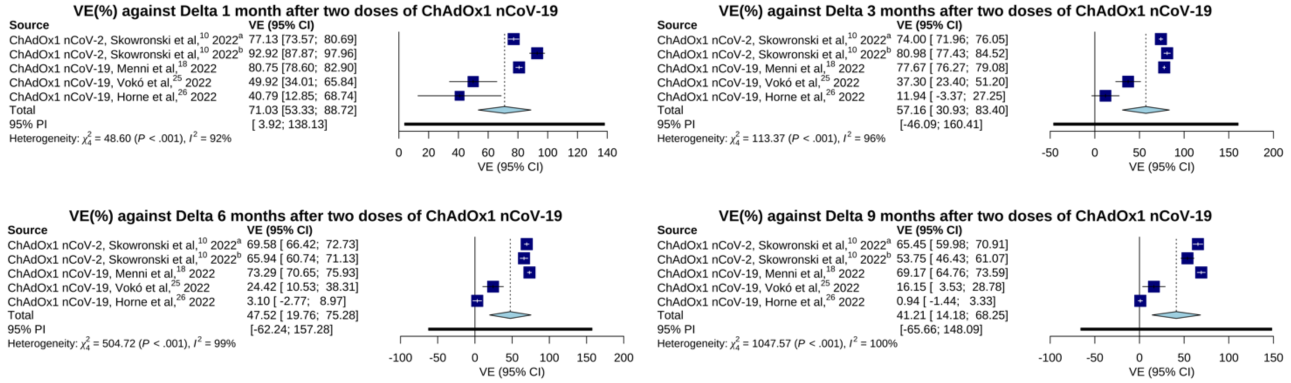

eFigure 39. Pooled Estimates of VE Against Any Laboratory-Confirmed Infection With Delta at 1, 3, 6, and 9 Months After Two Doses of ChAdOx1 nCoV-19

[a] Data from British Columbia; [b] Data from Quebec.

© 2023 Menegale F et al. *JAMA Network Open.*

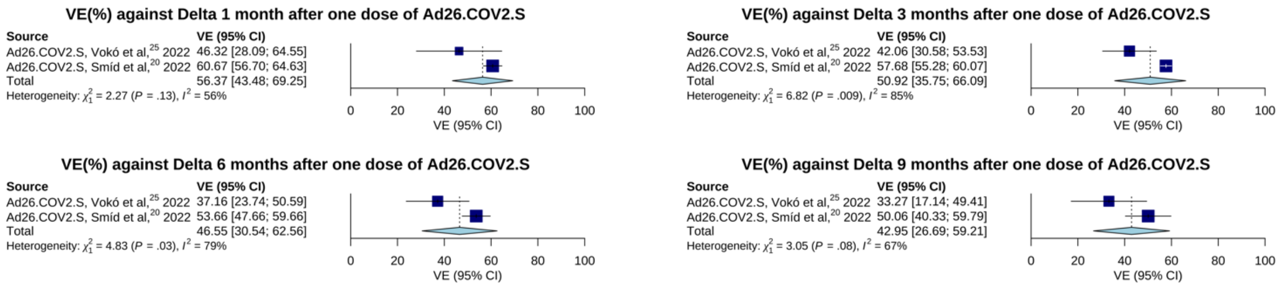

eFigure 40. Pooled Estimates of VE Against Any Laboratory-Confirmed Infection With Delta at 1, 3, 6, and 9 Months After One Dose of Ad26.COV2.S.

© 2023 Menegale F et al. *JAMA Network Open.*
