# Supplementary material for: Evaluation of Waning of SARS-CoV-2 Vaccine–Induced Immunity: A Systematic Review and Meta-analysis
Source: JAMA Netw Open. 2023 May 3;6(5):e2310650. doi: 10.1001/jamanetworkopen.2023.10650 (PMC10157431; doi:10.1001/jamanetworkopen.2023.10650)
Supplement: Supplement 2. — Data Sharing Statement [file jamanetwopen-e2310650-s002.pdf]

Data Sharing Statement

Menegale. Evaluation of Waning of SARS-CoV-2 Vaccine-Induced Immunity. *JAMA Netw Open*. Published May 03, 2023. doi:10.1001/jamanetworkopen.2023.10650

Data
Data available: Yes
Data types: Data (not involving human participants)
How to access data: Only secondary data are analysed. All estimates used in this study were retrieved from selected publications.
When available: With publication

Supporting Documents
Document types: None

Additional Information
Who can access the data: anyone would have access to published data
Types of analyses: for any purpose
Mechanisms of data availability: Only secondary data are analysed. All estimates used in this study were retrieved from selected publications and will be available without investigator support.
